# Risk factors for progression of age‐related macular degeneration

**DOI:** 10.1111/opo.12675

**Published:** 2020-02-25

**Authors:** Thomas J Heesterbeek, Laura Lorés‐Motta, Carel B Hoyng, Yara T E Lechanteur, Anneke I den Hollander

**Affiliations:** ^1^ Department of Ophthalmology Donders Institute for Brain, Cognition and Behaviour Radboud University Medical Center Nijmegen The Netherlands; ^2^ Department of Human Genetics Donders Institute for Brain, Cognition and Behaviour Radboud University Medical Center Nijmegen The Netherlands

**Keywords:** age‐related macular degeneration, epidemiology, genetics

## Abstract

**Purpose:**

Age‐related macular degeneration (AMD) is a degenerative disease of the macula, often leading to progressive vision loss. The rate of disease progression can vary among individuals and has been associated with multiple risk factors. In this review, we provide an overview of the current literature investigating phenotypic, demographic, environmental, genetic, and molecular risk factors, and propose the most consistently identified risk factors for disease progression in AMD based on these studies. Finally, we describe the potential use of these risk factors for personalised healthcare.

**Recent findings:**

While phenotypic risk factors such as drusen and pigment abnormalities become more important to predict disease progression during the course of the disease, demographic, environmental, genetic and molecular risk factors are more valuable at earlier disease stages. Demographic and environmental risk factors such as age and smoking are consistently reported to be related to disease progression, while other factors such as sex, body mass index (BMI) and education are less often associated. Of all known AMD variants, variants that are most consistently reported with disease progression are rs10922109 and rs570618 in *CFH*, rs116503776 in *C2/CFB/SKIV2L*, rs3750846 in *ARMS2/HTRA1* and rs2230199 in *C3*. However, it seems likely that other AMD variants also contribute to disease progression but to a lesser extent. Rare variants have probably a large effect on disease progression in highly affected families. Furthermore, current prediction models do not include molecular risk factors, while these factors can be measured accurately in the blood. Possible promising molecular risk factors are High‐Density Lipoprotein Cholesterol (HDL‐C), Docosahexaenoic acid (DHA), eicosapentaenoic acid (EPA), zeaxanthin and lutein.

**Summary:**

Phenotypic, demographic, environmental, genetic and molecular risk factors can be combined in prediction models to predict disease progression, but the selection of the proper risk factors for personalised risk prediction will differ among individuals and is dependent on their current disease stage. Future prediction models should include a wider set of genetic variants to determine the genetic risk more accurately, and rare variants should be taken into account in highly affected families. In addition, adding molecular factors in prediction models may lead to preventive strategies and personalised advice.

## Introduction

Age‐related macular degeneration (AMD) is a degenerative disease of the macula, often leading to progressive vision loss. AMD is the most prevalent retinal disease in the Western world with approximately 1–3% of the total population suffering from an advanced stage of the disease.^1^, ^2^, ^3^ The rate of disease progression can vary among individuals and studies have identified several risk factors for a faster disease progression. The potential benefit of identifying these risk factors is the ability to predict disease progression in individual patients but also to improve the design of clinical trials. With the development of new imaging techniques and the progress in genetic and molecular technologies, new risk factors for disease progression have been identified but not all have been assessed in comprehensive prediction models.

### Aim of this review

The aim of this review is to provide an overview of the current literature investigating phenotypic, demographic, environmental, genetic, and molecular risk factors for disease progression in age‐related macular degeneration. In addition, we describe the most promising risk factors that could improve current prediction models for personalised healthcare.

### Methods of literature search

A comprehensive review of literature was performed through a PubMed search in July 2019. We used the following keywords and their synonyms in various combinations: age‐related macular degeneration, prospective cohort study, follow‐up, progression, risk factors and prediction. When a specific risk factor was identified, the specific risk factor was also used as a keyword in a second PubMed search to identify additional publications with prospective data on the specific risk factor. Articles cited in the reference list of articles obtained through this search were also reviewed whenever relevant. After article selection, all specific risk factors were grouped based on their corresponding risk category and results were discussed accordingly. A complete overview of the studies and references is provided in Tables S1–S6.

### Definition of progression in age‐related macular degeneration

Disease progression in AMD has been defined using various approaches. Although deteriorating vision seems a logical outcome measure for defining AMD progression in natural history studies or clinical trials, it is often unrealistic to use visual acuity as an endpoint since vision loss may take years to develop. For this reason, studies on AMD have used anatomical endpoints to measure disease progression over a relatively short time span.^4^ The most often used anatomical endpoint in AMD is the development of late AMD, which can be divided into two subtypes: geographic atrophy (GA) and choroidal neovascularisation (CNV).

#### Progression to geographic atrophy

GA, also referred to as dry AMD, is characterised by the loss of photoreceptors, retinal pigment epithelium (RPE) and choriocapillaris, causing a gradual loss of vision over time.^5^, ^6^ AMD progression can be defined as the conversion of an early stage of AMD to GA.^4^ Three major imaging strategies have been used to document conversion to GA: colour fundus photography (CFP), fundus autofluorescence (FAF) and optical coherence tomography (OCT). On CFP it can be challenging to identify early signs of GA development and to reliably establish the margins of GA, whereas FAF and OCT imaging are more suitable for this purpose. On FAF, GA is identified by the absence of a fluorescence signal, which is assumed to correlate with the absence of RPE, delineating the boundaries of the GA lesion.^7^ However, FAF imaging does not always reveal a GA lesion when OCT imaging detects GA.^8^ The Classification of Atrophy Meetings (CAM) program proposed the OCT as the standard reference or base imaging method to diagnose and stage GA.^9^ GA can develop into different stages, depending on the disappearance of the photoreceptor layer and RPE layer. According to the CAM classification, GA can be subdivided into: incomplete outer retina atrophy (iORA, demonstrating thinning of the outer retina with an intact RPE band and no hypertransmission of light into the choroid below Bruch's membrane (BM)), complete outer retina atrophy (cORA, showing severe thinning of the outer retina, in the setting of an intact RPE band with intermittent hypertransmission of light), incomplete RPE and outer retinal atrophy (iRORA, showing the degeneration of photoreceptors, an irregular or interrupted RPE band and discontinued hypertransmission of light) and finally complete RPE and outer retinal atrophy (cRORA, showing the degeneration of photoreceptors and a zone of complete disrupted RPE band of at least 250 µm in diameter with the hypertransmission of light).

#### Geographic atrophy growth

When GA has already developed, the growth rate of GA may be used as a quantifiable measure for disease progression.^4^ Clinical trials have been using the GA growth rate as an outcome measurement to investigate the effects of new developed drugs. These studies use mainly CFP or FAF imaging to measure the growth of the GA lesions. Yet, OCT is recommended as the standard reference to define the borders of the GA lesion.^9^

#### Progression to neovascular age‐related macular degeneration

CNV, also referred to as wet AMD or neovascular AMD (nAMD) is characterised by the formation of new fragile vessels that originate from the choriocapillaris. These new vessels grow into the retina with subsequent leakage and/or hemorrhage, which can result in serous RPE detachment accompanied by a rapid loss of vision and eventually provoking a vision‐threatening scar in the macula.^10^ Imaging modalities that can identify the conversion of an early stage of AMD to nAMD are CFP, OCT, fluorescein angiography (FAG), indocyanine green angiography (ICGA) and OCT‐angiography (OCTA). Whereas CFP, OCT, and FAG can identify exudative nAMD by fluid leakage and haemorrhaging, in some cases a CNV can already be visualised before exudation occurs using ICGA and OCTA imaging.^11^

#### Progression at early disease stages

Central GA and nAMD are strongly associated with the loss of vision, but both advanced stages can take years to develop. Therefore, conversion to GA or nAMD is not always the best outcome measure for disease progression in natural history studies or clinical trials. In addition, advanced stage AMD may be too late in the disease process to demonstrate the effectiveness of newly developed treatments, which may be more effective when administered earlier in the disease process.^4^ Therefore, several AMD severity scales have been developed in an effort to provide endpoints to monitor AMD progression at earlier disease stages.^12^, ^13^ The most commonly used anatomical endpoints in these severity scales are the appearance and progression of drusen, but also other phenotypic features that are associated with the development of advanced stage AMD, can be used to investigate disease progression at earlier disease stages. In this review, we included prospective studies that evaluated the incidence of early AMD (based on the appearance of drusen and/or pigment abnormalities), as well as the progression to late AMD, including GA and nAMD, and GA growth.

## Phenotypic risk factors

### Drusen

Drusen are deposits of extracellular debris between the retinal pigment epithelium (RPE) and Bruch's membrane (BM). Drusen may be a manifestation of the normal ageing process or can represent an early sign of AMD, depending on the number, size, shape, distribution, and morphology of the drusen.^14^ Studies have shown that drusen are dynamic structures that demonstrate repeated cycles of increased and decreased volume, but, overall, drusen are more likely to grow.^15^, ^16^ When individual drusen decrease in size, they may result in no residual anatomic defects or they may evolve into GA or nAMD.^17^, ^18^, ^19^, ^20^ The total number of drusen or measured drusen area or volume, are risk factors for the progression to GA and nAMD.^16^, ^17^, ^21^, ^22^, ^23^, ^24^, ^25^, ^26^ Also, the location of the drusen is of predictive value for disease progression, since eyes with drusen near the fovea have a higher probability of developing late AMD compared to eyes with drusen outside the fovea.^21^, ^23^, ^27^ Although CFP is useful for assessing the appearance of drusen, multimodal imaging can be used to distinguish subtypes of drusen.^28^

#### Small drusen

**Figure 1:**
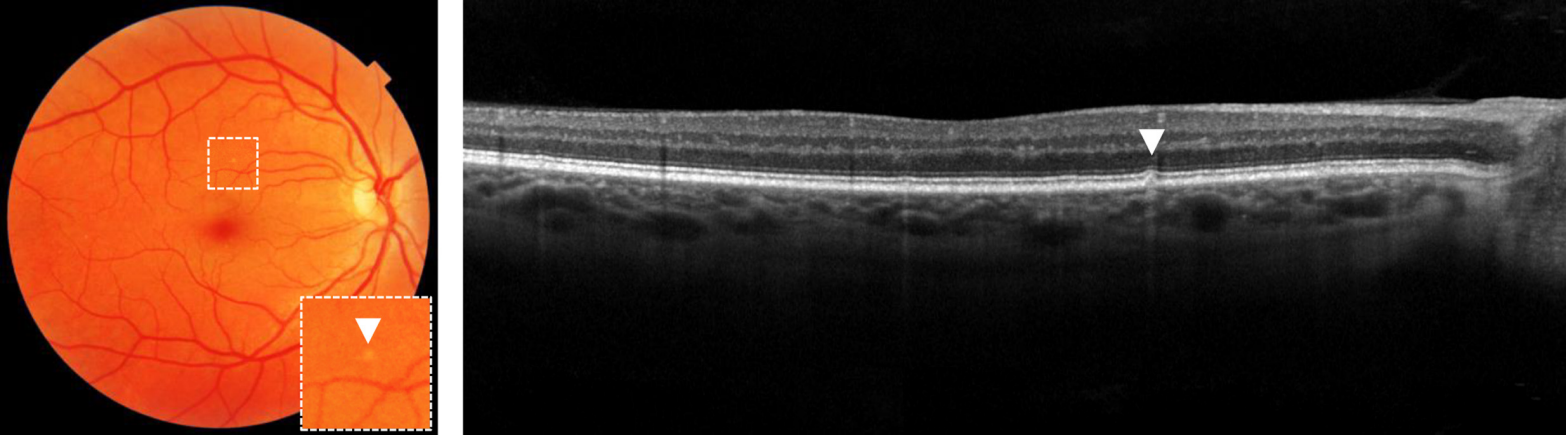
Hard druse on colour fundus photograph, as indicated with an arrow (left) and optical coherence tomography, as indicated with an arrow (right).

Small drusen are classified as discrete yellow‐white deposits (≤63 µm in diameter) with clear defined margins and are located between the RPE and BM (*Figure* 1).^14^ Individuals over 55 years of age with a few small drusen, called “drupelets”, which are considered to be a normal ageing process of the eye, have a low probability (0.4%) of developing late AMD within five years.^29^ Nevertheless, AMD frequently begins with small drusen, which might accumulate in number over time.

#### Medium and large drusen

**Figure 2:**
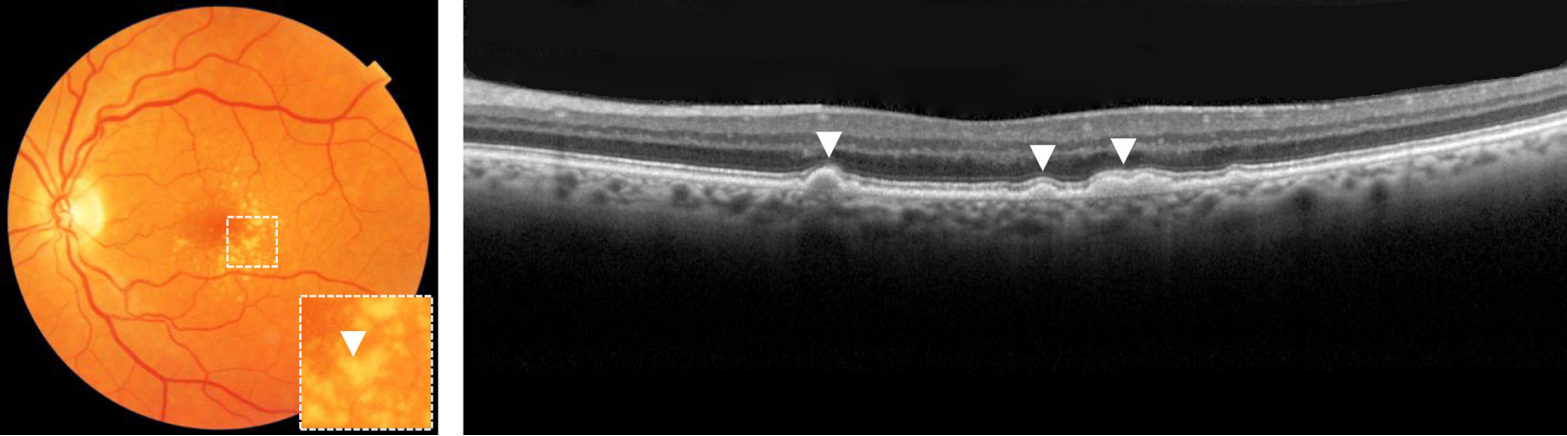
Large drusen on colour fundus photograph, as indicated with an arrow (left) and optical coherence tomography, as indicated with arrows (right).

Medium drusen (>63 µm and ≤125 µm in diameter) and large drusen (>125 µm in diameter) are large focal yellow‐white deposits with unclearly defined margins and are located between the BM and RPE, most often in the central macula (*Figure* 2).^14^ In the most grading protocols, patients with medium drusen without pigmentary abnormalities are considered to have early stage AMD. Patients with medium drusen and pigmentary abnormalities, or with at least large drusen have intermediate stage AMD.^29^ Especially large drusen are associated with progression towards late stage AMD, in particular GA.^18^, ^20^, ^23^, ^30^, ^31^, ^32^ Depending on the absence or presence of pigment abnormalities, patients with medium drusen have a probability between 2% and 20% of developing late AMD within five years, while patients with large drusen have a probability between 13% and 47% of developing late AMD within five years.^29^ Large drusen that increase rapidly in size over time are more at risk for RPE cell migration upon the druse with eventually a drusen collapse and the formation of GA.^33^, ^34^

#### Calcified drusen

**Figure 3:**
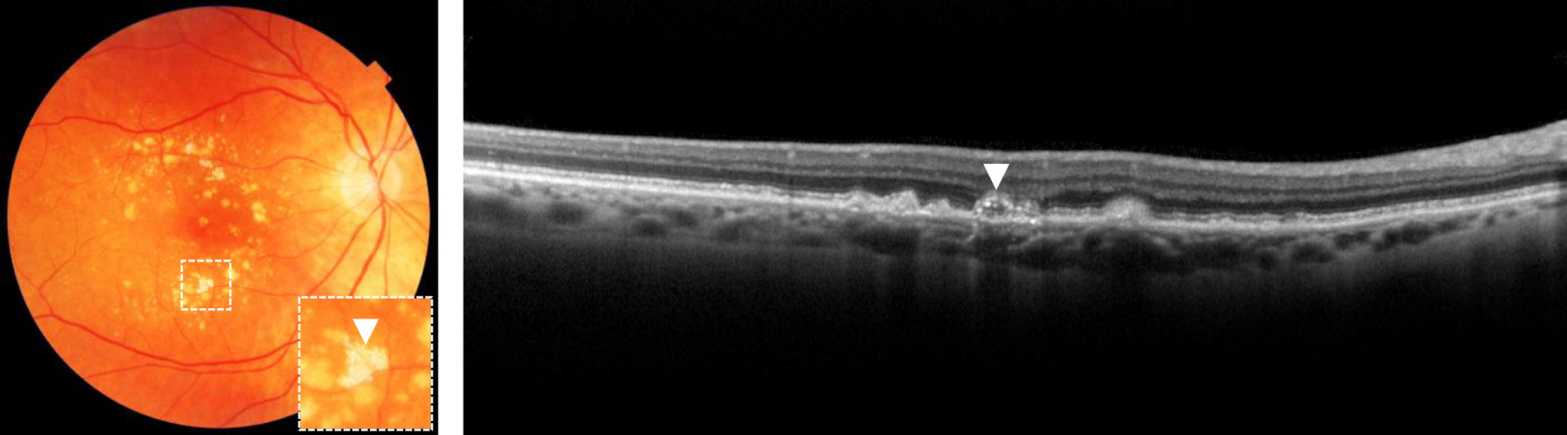
Calcified druse on colour fundus photograph, as indicated with an arrow (left) and optical coherence tomography, as indicated with an arrow (right).

When drusen exist for a longer duration, they can undergo calcification which gives the druse a glistening appearance on CFP. On OCT, calcified drusen can be identified as a druse with heterogeneous hyperreflective structures. (*Figure* 3).^14^ Calcification of drusen is usually a sign of drusen regression, with eventually the development of RPE atrophy above the druse leading to GA.^18^, ^35^, ^36^. Patients with calcified drusen have a probability of 26% of developing GA within five years.^35^

#### Reticular pseudodrusen

**Figure 4:**
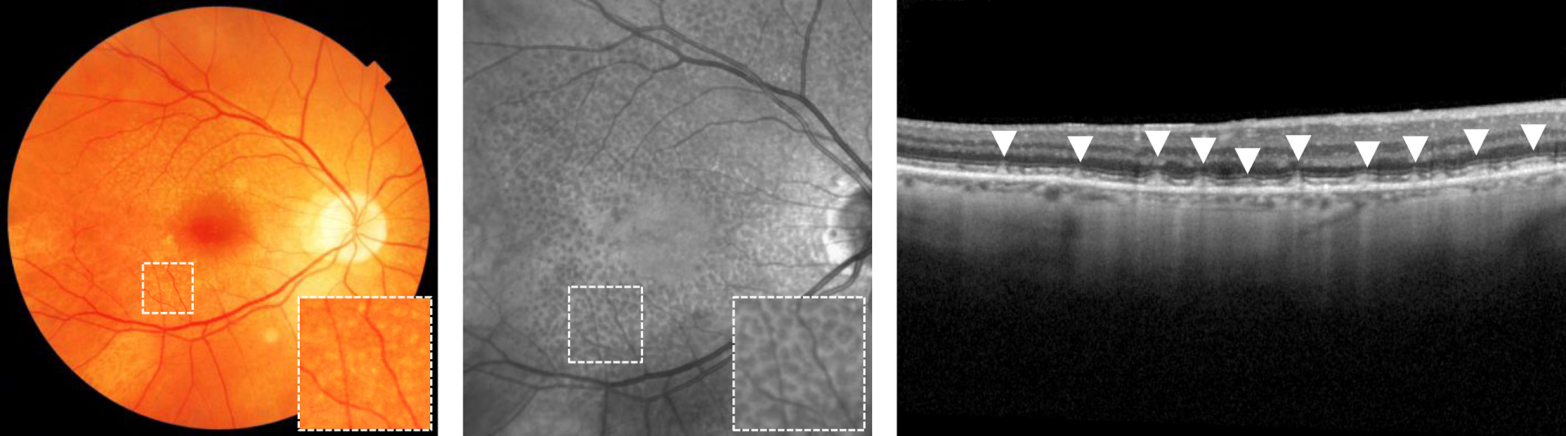
Reticular pseudodrusen on colour fundus photograph (left), near‐infrared photography (middle) and optical coherence tomography, as indicated with arrows (right).

Reticular pseudodrusen (RPD) are dot‐like subretinal deposits between the RPE and photoreceptor layer which most frequently first appear in the upper part of the retina and then extend towards the macula over time, forming interlacing networks.^14^ Although visible on CFP, RPD are best visualised using near‐infrared (NIR) photography.^37^ Using CFP and NIR photography, RPD can still easily be misdiagnosed for small drusen. OCT is needed to confirm the RPD by their position between the RPE and photoreceptor layer (*Figure* 4).^28^ RPD are highly prevalent in fellow‐eyes of patients with unilateral nAMD. Although these patients with unilateral nAMD are at high risk of progression, observing the presence of RPD in fellow‐eyes renders an additional risk of progression.^30^, ^32^, ^38^, ^39^ RPD are of prognostic significance in the progression of AMD and are independently associated with progression towards GA (progression rate of 15.3% in two years) and nAMD (progression rate of 30.7% in two years) regardless of the disease stage based on other features.^23^, ^30^, ^32^, ^38^, ^39^, ^40^, ^41^ In addition, dot pseudodrusen are more often associated with the development of nAMD (relative risk [RR] 2.53), while confluent pseudodrusen are associated with the development of GA (RR 4.35).^41^ Eyes with RPD have also an increased probability of GA growth within three years follow‐up (74–77%) compared to eyes without RPD (42–53%).^42^, ^43^

#### Cuticular drusen

**Figure 5:**
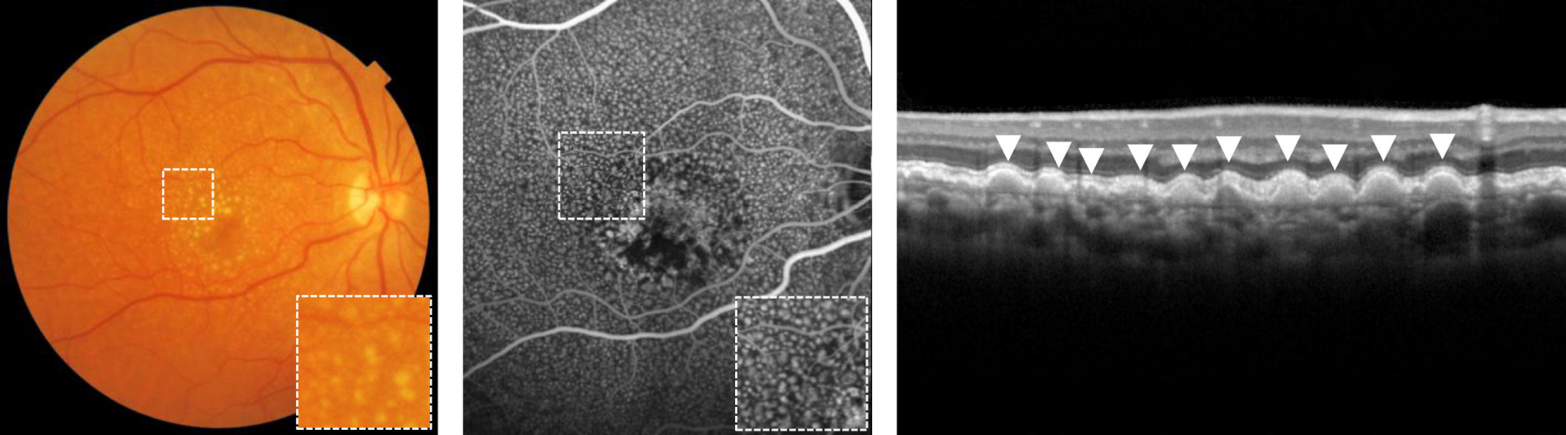
Cuticular drusen on colour fundus photograph (left), fluorescein angiography (middle) and optical coherence tomography, as indicated with arrows (right).

Cuticular drusen are multiple small drusen that may cluster between the BM and RPE layer, often first visible in the peripheral retina and later in the macula.^14^ Cuticular drusen have a characteristic hyperfluorescent stars‐in‐the‐sky appearance, which is best identified using FAG. Individually they may be mistaken for small drusen or dot RPD, but because of their saw‐tooth appearance and spheroid or triangular shape on OCT, they can still be identified as cuticular drusen (*Figure* 5).^28^ Genetic studies show, cuticular drusen are associated with variants in the *CFH* gene.^44^ They are predictive for the development of nAMD (progression probability of 8.7%‐12.5% in five years) and GA (progression probability of 25.0%‐28.4% in five years).^45^, ^46^

### Changes of the retinal pigment epithelium

#### Pigmentary changes

**Figure 6:**
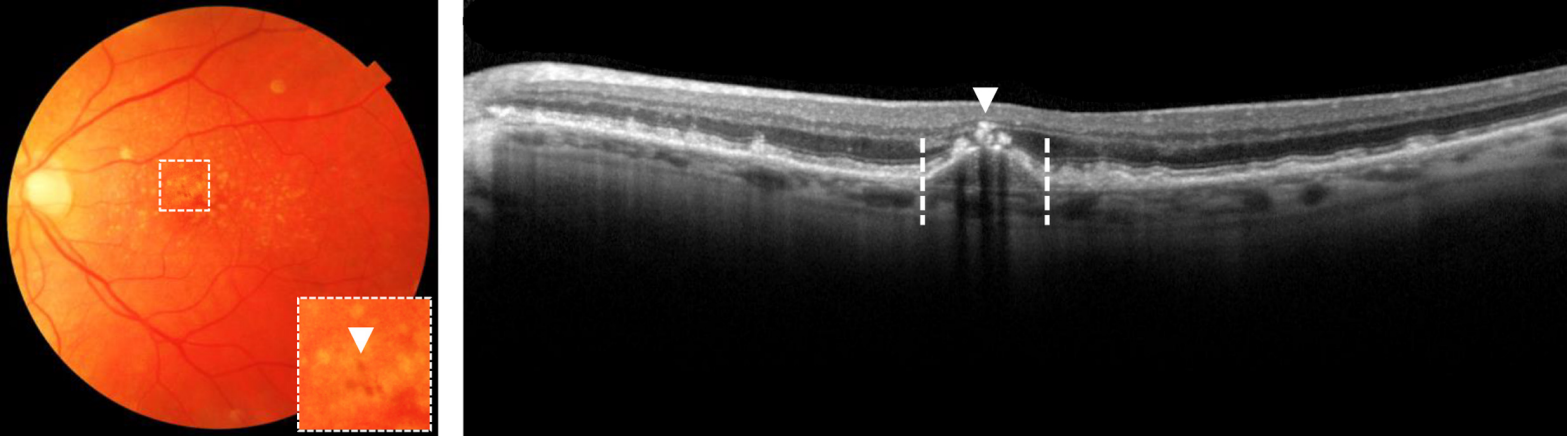
Hyperpigmentation on top of a large druse on colour fundus photograph, as indicated with an arrow (left), hyperreflective foci (indicated with arrow) upon a drusenoid pigment epithelial detachment (indicated between dotted lines) on optical coherence tomography (right).

Pigmentary changes in the macula are a feature of early AMD. Pigmentary changes appear on CFP either as hypopigmentation, which is seen as depigmented areas of the RPE not meeting criteria for GA, or as hyperpigmentation, which are deposits of pigment from the RPE within the retina (*Figure* 6). The involvement of pigmentary changes in addition to drusen increases the risk of developing late AMD drastically.^20^, ^21^, ^23^, ^30^, ^31^, ^47^ Patients with large drusen and pigmentary changes have a probability of 47% of developing late AMD within five years.^29^

#### Hyperreflective foci

Hyperreflective foci (HRF) on OCT are lesions of equal or higher reflectivity than the RPE band, located in the neuroretina, often above drusen, and are associated with the hyperpigmentation on CFP (*Figure* 6).^48^ Studies suggest that HRF may represent the migration of RPE cells caused by cytokines and inflammatory mediators in response to oxidative stress and complement activation.^49^ HRF are associated with progression to nAMD and GA,^17^, ^19^, ^50^, ^51^ and the progression rates seems to be the same for both late AMD stages: 47% of eyes with HRF develop nAMD after 24 months,^52^ and 50% of eyes with HRF develop GA within 28 months.^53^

#### Pigment epithelial detachment

Pigment epithelial detachment (PED) is characterised by the separation of the RPE from the BM. PEDs can be classified into fibrovascular, serous and drusenoid types. Fibrovascular PEDs and serous PEDs are both associated with nAMD and are caused by the accumulation of fluid between the BM and RPE. On OCT, fibrovascular PEDs are RPE detachments which have a flattened or notched border and are frequently associated with a hidden CNV (see section on Quiescent choroidal neovascularisation NV/Subclinical choroidal neovascularisation). Serous PEDs appear as a large dome‐shaped detachment of the RPE and are thought to develop by an increased hydrostatic pressure from an underlying CNV.^54^ Drusenoid PEDs develop from the coalescence of drusen and are associated with the progression to GA (probability of 19% within five years),^18^, ^47^, ^55^, ^56^ progression to nAMD (probability of 23% within five years)^47^, ^50^, ^53^, ^55^, ^56^ and the development of calcified drusen and pigmentary changes (*Figure* 6).^47^

### Geographic atrophy features

#### Lesion size

Several studies have attempted to identify features of the GA lesions that are predictive for faster GA enlargement. Natural history studies show that the GA enlargement is dependent on the baseline GA area, showing an exponential increase in GA area over time. 57, 58, 59, 60, 61, 62 Of note, some studies suggest that this acceleration is dependent on the GA lesion at a specific time point, showing an acceleration of the GA growth in earlier stages of GA and deacceleration of GA growth at later stages of GA when there are no remaining RPE cells in the macula.^4^, ^63^ However, when the GA enlargement is adjusted for the baseline lesion size by using a square root transformation, the lesion itself seems to have less influence on the GA growth. These studies report a mean GA enlargement between 0.23–0.28 mm year^−1^, independent of the lesion size.^61^, ^62^, ^63^, ^64^

#### Lesion number and location

Multiple studies have reported increased rates of GA growth in eyes with multifocal lesions (0.32–0.42 mm year^−1^ or 0.9–1.97 mm^2^ year^−1^) as compared to eyes with unifocal lesions (0.19–0.26 mm year^−1^ or 0.3–1.05 mm^2^ year^−1^).^42^, ^43^, ^59^, ^61^, ^62^ Multifocal lesions may have increased GA growth since they have a larger border of the active disease process. The location of the lesion is also found to be associated with the progression of the GA. Extrafoveal lesions seem to have faster growth rates (0.31–0.33 mm year^−1^ or 2.05 mm^2^ year^−1^) when compared to foveal lesions (0.22–0.27 mm year^−1^ or 1.28 mm^2^ year^−1^).^57^, ^59^, ^62^, ^63^

#### Lesion shape

The shape of the GA lesion is also an indicator of disease progression. One study showed that the Geographic Atrophy Circularity Index (GACI), a measurement of the circularity of the GA lesion based on the GA area and its perimeter, is associated with the progression rate of GA. GA lesions with a circular shape (GACI > 0.75) have a slower growth rate (0.21 mm year^−1^) when compared to GA lesions with an irregular shape (GACI < 0.25, 0.40 mm year^−1^).^62^

#### Perilesional fundus autofluorescence pattern

**Figure 7:**
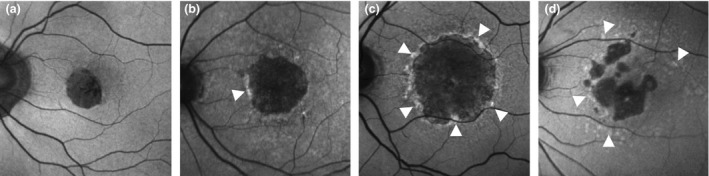
Overview of perilesional fundus autofluorescence patterns, from left to right: no pattern (a), focal pattern (b), banded pattern (c) and diffuse pattern (d).

The perilesional FAF pattern surrounding the GA lesions has been shown to correlate with the growth rates of GA. These FAF patterns can be subdivided into multiple categories, based on the position of the hyperfluorescence. (*Figure* 7). Progression rates of GA are lower in eyes with no FAF patterns (0.20–0.38 mm^2^ year^−1^) and focal FAF patterns (0.53–0.81 mm^2^ year^−1^), while progression rates of GA are higher in eyes with banded (1.69–1.81 mm^2^ year^−1^) and diffuse FAF patterns (1.74–1.77 mm^2^ year^−1^). These results have been replicated in multiple studies.^59^, ^65^, ^66^, ^67^ However, one prospective study reported that the effect of the perilesional FAF pattern on GA growth is mainly caused by the baseline lesion size, suggesting that the perilesional FAF pattern is a consequence of GA growth and not the cause.^57^

### Vascular features

#### Choroidal abnormalities

Several studies found evidence for the importance of the choroid in the pathogenesis of AMD established that the choroid vascular structures deplete in eyes with early AMD, GA and nAMD.^68^, ^69^ One prospective study identified that irregular choroidal vessels are predictive for both the development of GA and nAMD.^50^ In addition, a smaller choroidal thickness was associated with the development of GA in one prospective study,^19^ but these findings could not be replicated.^32^

#### Choriocapillaris flow impairments

OCT angiography (OCTA) can detect blood flow by analysing changes in tissue reflectivity between rapidly acquired images, visualising the blood flow of the retina and the choroidal vasculature. Although OCTA is still developing as an imaging modality, it has already demonstrated some potential features for predicting GA growth. Flow impairment in the choriocapillaris surrounding the atrophic lesions was associated with the increased GA growth rate over 1.3 years of follow‐up in one study.^70^ However, these findings have not yet been replicated.

#### Quiescent choroidal neovascularisation/Subclinical choroidal neovascularisation

**Figure 8:**
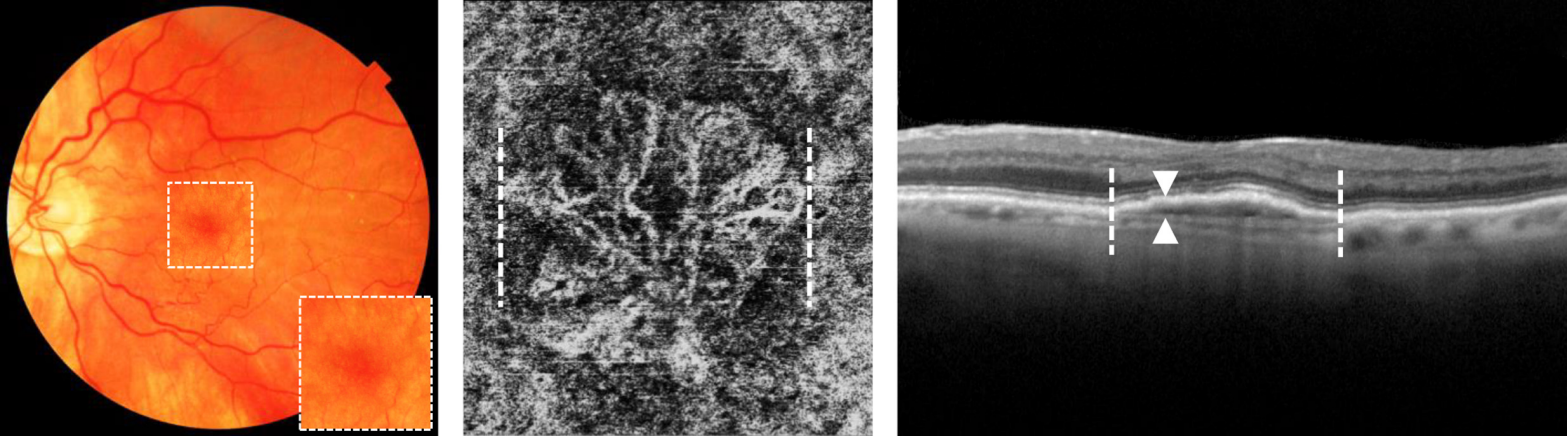
No haemorrhaging or oedema on colour fundus photograph (left) identified quiescent choroidal neovascularisation (CNV)/subclinical choroidal neovascularisation (CNV) just below the pigment epithelium as shown between the dotted lines on optical coherence tomography angiography (middle) and a double layer sign showing an irregularly slightly elevated pigment epithelium as indicated between the arrows, at the site of the quiescent CNV/subclinical CNV (indicated between the dotted lines) on optical coherence tomography (right),

Using ICGA or OCTA, it is also possible to visualise a CNV in nAMD before exudation occurs (*Figure* 8). When these treatment‐naïve CNVs present over at least 6 months without any signs of exudation, these CNVs are defined as quiescent CNVs or subclinical CNVs.^71^ Quiescent CNVs appear as a hyperfluorescent plaque on ICGA, and as a network of vessels between the BM and RPE on OCTA. On OCT, a double layer sign can be identified at the site of the quiescent CNV, showing a irregularly slightly elevated RPE, without hyporeflective intraretinal/subretinal fluid.^71^ Prospective studies show that eyes with a quiescent CNV have a 15.2–18.1 higher risk of becoming exudative, as compared to eyes lacking a precursor quiescent CNV.^72^, ^73^, ^74^ Whether eyes with quiescent CNV should be more often monitored or treated preventively with anti‐VEGF injections to prevent exudation is not yet clear.

### Other imaging features

#### Incomplete retinal pigment epithelium and outer retina atrophy (iRORA)

**Figure 9:**
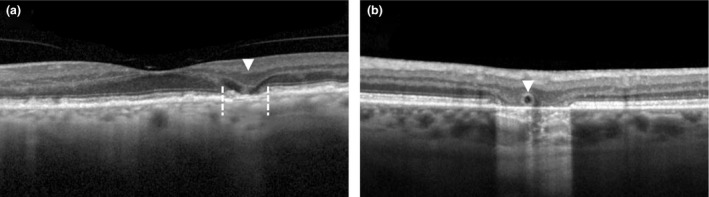
Incomplete retinal pigment epithelium atrophy and outer retina atrophy (iRORA) on optical coherence tomography as seen by irregular or interrupted RPE layer, indicated between dotted lines and a subsided outer plexiform layer and inner nuclear layer, indicated with an arrow (a), and an outer retinal tubulation on optical coherence tomography, as indicated with an arrow (b).

Incomplete RPE and outer retinal atrophy (iRORA) is a feature preceding the development of complete RPE and outer retina atrophy (cRORA).^8^, ^50^ iRORA includes a discontinued hypertransmission of light below the RPE, an irregular or interrupted RPE layer, and a subsided outer plexiform layer and inner nuclear layer (*Figure* 9 *a*).^9^ A total of 74% of the eyes that meet incomplete RORA develop complete RORA within 5 years.^50^

#### Outer retinal tubulations

Outer retinal tubulations (ORTs) can be identified on OCT as circular hyperreflective band structures in the outer nuclear layer (*Figure* 9 *b*). They develop in response to injury as Müller cells move between the functional and dead photoreceptors. ORTs can both be found in areas of fibrosis after the development of exudative nAMD, or at the border of GA. In nAMD, eyes with ORTs often have a larger decline in visual acuity over time than those without ORTs.^75^ However, in patients with GA, ORTs at the border of the GA lesion are associated with a slower rate of GA enlargement when compared to those without ORTs.^76^

## Demographic and environmental risk factors

### Age

There are many modifiable and non‐modifiable risk factors that have been linked to increased risk of the progression of AMD. As the name already suggests, age is the most important demographic risk factor for AMD.^20^, ^21^, ^23^, ^27^, ^31^, ^32^, ^40^, ^77^, ^78^, ^79^, ^80^, ^81^, ^82^, ^83^, ^84^, ^85^, ^86^, ^87^, ^88^, ^89^, ^90^, ^91^, ^92^, ^93^, ^94^, ^95^ A recent meta‐analysis, combining data of 14 population‐based studies, showed an increase in the prevalence of early AMD from 3.5% in people aged 55–59 years up to 17.6% in people 85 years and older. For late AMD, these prevalence rates increase from 0.1% to 9.8%, respectively.^96^ Prospective studies show that age is slightly stronger associated with the progression to GA (HR 1.14–1.18, per year increase)^23^, ^40^ than to nAMD (HR 1.08, per year increase).^94^ Higher age is the main risk factor for AMD since ageing is associated with structural and functional changes of the retina that predispose the development of AMD, and contributes to the additive effects of other pathological risk factors over time.^97^

### Sex

While AMD is common in males and females, some studies suggest that female sex is associated with a higher progression rate to early AMD (OR 2.2),^40^, ^85^ and late AMD (HR 1.6–2.6),^84^, ^87^, ^94^ especially nAMD (HR 1.6–2.1).^27^, ^77^, ^94^ However, other studies describe a lack of association between sex and disease progression.^20^, ^23^, ^32^, ^57^, ^78^, ^80^, ^81^, ^83^, ^86^, ^89^, ^91^, ^92^, ^93^, ^95^, ^98^ A possible explanation for these conflicting results between studies might be attributed to a difference in follow‐up and the greater life expectancy of females.^99^ However, some studies suggest that AMD progression might indeed follow different processes in females due to differences in sex hormones, such as estrogens. Estrogens may lead to favorable alterations in serum lipids and may exert antioxidant properties. Some cross‐sectional studies have found evidence of a protective effect of estrogen exposure in females. Females using hormone replacement therapy had a decreased risk of having AMD,^100^ and females who reached menopause at an earlier age had an increased risk of having AMD.^101^ However, other studies did not confirm these findings.^2^, ^102^, ^103^, ^104^, ^105^ Longitudinal analyses of prospective cohorts are required to enhance our understanding of the association between estrogen exposure and the risk of AMD.

Another hypothesis for sex‐associated differences in AMD progression may be caused by the number of X‐linked genes which may affect cellular functions in the onset and progression of AMD.^106^ No AMD‐associated variants have been identified in X‐linked genes, but epigenetic mechanisms might have a role in the pathogenesis of AMD by acting on X‐linked genes.

### Smoking

Smoking is the most consistently reported modifiable risk factor for AMD and is associated with a 2–4 fold increased risk for any form of AMD,^20^, ^21^, ^31^, ^63^, ^94^ early AMD^40^, ^85^, and late AMD^32^, ^85^, ^87^, ^91^, ^107^ including nAMD^92^ and GA,^2^, ^23^, ^92^ and is associated with a faster GA growth (0.33 mm year^−1^ in smokers compared to 0.27 mm year^−1^ in non‐smokers).^63^ Several studies have also compared pack‐years of smoking, and most of them confirmed an additive dose‐response effect.^21^, ^77^, ^84^, ^108^ Even after quitting smoking, ex‐smokers still have a modestly increased risk of disease progression compared to never‐smokers,^87^ although this association is not always found.^2^, ^57^, ^80^, ^81^, ^82^, ^86^, ^88^, ^93^, ^98^, ^109^, ^110^, ^111^ Quitting smoking reduces the risk of developing AMD and after 20 years cessation of smoking, risk probabilities of developing AMD seem to be comparable to that of non‐smokers.^112^ Cigarette smoke is known to contain toxic compounds that may have pathological effects through different biochemical pathways, including the formation of retinal oxidative stress (see section on oxidative stress factors) and inflammation in RPE cells and vascular changes in the choroidal vessels.

### Body composition

Having a higher body mass index (BMI) is found to be associated with increased probabilities of developing AMD in half of the studies,^77^, ^84^, ^86^, ^92^, ^94^, ^98^, ^110^, ^113^ while other studies found no association.^2^, ^21^, ^87^, ^88^, ^93^, ^108^, ^109^, ^111^, ^114^, ^115^, ^116^ In comparison with individuals with a normal weight (BMI 20–25), obese individuals (BMI > 30) have an increased risk for developing late AMD (OR 1.2–2.2).^77^, ^84^, ^86^, ^91^, ^92^ Also other measurements of body weight such as waist circumference (WC) and waist‐hip ratio (WHR) have been studied and confirmed the associated between body weight and disease progression.^110^, ^113^ For example, one longitudinal population cohort found that a decrease in WHR is associated with a lower incidence rate of any form of AMD (per 3% WHR decline, OR 0.71). This effect was even more pronounced in obese patients (per 3% WHR decline, OR 0.41).^117^

Studies have shown that pro‐inflammatory factors, such as complement components and cytokines are elevated in obese individuals. These proinflammatory factors regulate inflammation and could disturb the functions of the RPE, which contributes to the development of AMD. In addition, adipose tissue is a storage site of protective carotenoids (see section on carotenoids) and as the bodyweight increases, more carotenoids would be absorbed into adipocytes and as a consequence less of these carotenoids would be available in the macula.^118^

### Diet

Diet is found to have a potential role in preventing and/or delaying the progression of AMD. High adherence to the Mediterranean diet reduces the risk of developing late AMD, which has been confirmed in two prospective cohorts,^119^, ^120^ while another prospective cohort did not find any association.^85^ The Mediterranean diet is naturally rich in antioxidants and typically characterised by high consumption of fruits, vegetables, legumes, grains and nuts, moderate consumption of fish, poultry, and dairy, and limited consumption of red meat. Olive oil rich in unsaturated fatty acids is used instead of butter and low to moderate amounts of red wine may be consumed.

In addition, other prospective studies investigating nutrition have found that the consumption of a high‐glycemic‐index diet (diet with carbohydrates that have a large and fast effect on blood glucose levels) is a risk factor for the development and progression of AMD.^121^, ^122^

Furthermore, individual components of the diet, such as the number of specific vegetables and fruits are also thought to be protective for disease progression since they are rich in antioxidants, including vitamins and carotenoids (see section on anti‐oxidative factors). Yet, individual diet components are not often found to be associated with disease progression in prospective studies.^119^ This is partly because dietary assessment methods for these foods are sensitive to bias since they rely on the participant's memory and have a day‐to‐day variability. Despite these methodological problems, multiple prospective studies were still able to confirm that fish consumption reduces the risk of disease progression in AMD.^23^, ^107^, ^119^, ^123^, ^124^, ^125^, ^126^, ^127^ Consumption of 1–2 servings fish per week has an OR of 0.48–0.58 for developing late AMD over 10 years of follow‐up. Fish is known to be rich in DHA and EPA, which are protective fatty acids (see section, Fatty acids).

### Physical activity

Regular exercise is thought to increase antioxidant enzyme activity and therefore physical activity may influence the progression of AMD. A few studies support the positive impact of physical activity on the progression of AMD, and physical activity is associated with lower odds of both early and late AMD.^85^, ^114^, ^116^, ^125^, ^128^ However, precise quantification of physical activity is difficult and therefore this factor is not suitable to use as a variable to predict AMD progression.^93^, ^111^, ^129^ Nevertheless, a small‐to‐moderate amount of physical activity should be advised to patients to confer retinal health benefit.

### Education

Education levels seem to be inversely related with the development of AMD in some longitudinal population studies, in which having an education level of high school or higher is associated with a lower risk for early AMD (OR 0.86)^82^ and late AMD (OR 0.57–0.73).^77^, ^87^, ^98^ In smaller cohort studies, this association is not always found.^84^, ^86^, ^91^, ^92^, ^93^, ^111^ Education itself is unlikely to be a direct risk factor, but rather a surrogate for other risk factors that are associated with a lower education level, including smoking, poor nutrition and less use of eye care services.

### Sunlight exposure

Sunlight exposure is thought to result in more oxidative stress in the retina, causing the development of AMD. However, the quantification of the total amount of sunlight exposure is difficult to determine and therefore not often found to be associated with disease progression.^77^, ^130^, ^131^ Nevertheless, sunglass protection should be advised to reduce retinal damage caused by sunlight exposure.

### Ethnicity

Multi‐ethnic studies show difference prevalence rates of any form of AMD between ethnic groups (2.4% Africans, 4.2% Hispanics, 4.6% Chinese, 5.4% Caucasians).^77^, ^132^, ^133^ Some hypothesise that the increased melanin in RPE cells of Africans may act as free radical scavenger or as a filter for ultraviolet radiation, which protects the RPE cells and Bruch's membrane, reducing the risk of AMD development or disease progression.^134^ In addition, nAMD seems to be more common in the Chinese population compared to Caucasians (OR 4.3).^132^ One possibility for this difference is that in some of these individuals, polypoidal choroidal vasculopathy (PCV) or a CNV caused by myopic maculopathy (MM) may be diagnosed. These retinal diseases mimic nAMD but without signs of drusen or RPE changes. Both retinal diseases are more common in the Asian population.^135^ Finally, it is possible that the differences in phenotypic characteristics of AMD differ between ethnic groups, perhaps related to racial differences in susceptibility genes for AMD.^136^

### Comorbidity

#### Cataract

Several large epidemiological studies have found an increased association between the development of cataract, cataract surgery and late AMD (both GA and nAMD),^137^, ^138^, ^139^ whereas others have demonstrated no association.^57^, ^77^, ^140^, ^141^ Some hypothesise that cataract surgery may increase the incidence of AMD and accelerate disease progression, possibly due to the induction of inflammatory reactions during and after surgery, and from increased exposure to ultraviolet light on the retina afterward.^142^ Others believe that cataract and AMD share environmental risk factors such as age, smoking, obesity, hypertension and sunlight exposure, and consequently, individuals who have had cataract surgery are automatically at higher risk for developing AMD as well.^143^ Besides, cataract surgery may even be beneficial for AMD patients in both visual acuity and quality‐of‐life parameters.^144^ Nevertheless, the association of cataract with AMD and the effect of cataract surgery remains a topic of active debate that is often revisited.

#### Hypertension

The possible involvement of hypertension in AMD has been well covered in literature,^21^, ^88^, ^95^, ^116^, ^145^ but is not always found to be a risk factor for AMD.^2^, ^40^, ^57^, ^77^, ^93^, ^94^, ^98^, ^108^, ^111^, ^115^, ^146^ High blood pressure is shown to be associated with lower choroidal blood flow and disturbed vascular homeostasis.^147^ Nevertheless, antihypertensive medication is not proven to have a positive effect on AMD. Although hypertension seems to play a role, it is unlikely to be a major contributor to the incidence and progression of AMD.

#### Chronic kidney disease

The association between chronic kidney disease has been reported in some studies, but evidence have been conflicting.^95^, ^148^, ^149^ One prospective cohort showed that, only in individuals older than 65 years, a decrease in the estimated glomerular filtration rate (eGFR) is associated with an increased probability of developing early AMD over fiveyears (per eGFR decrease of 10 mL min^−1^ 1.73 m^−2^, OR 1.3).^150^ In addition, cystatin C, a serum biomarker for kidney function, has also been associated with the development of early AMD and nAMD.^149^ Whether the association between AMD and chronic kidney disease (CKD) reflects a common causal pathway or shared risk factors such as age, chronic inflammation, and variants in the *CFH* gene, requires additional investigation.^148^

#### Hyperthyroidism

Longitudinal population studies show that thyroid disease causing hyperthyroidism has been implicated as a risk factor for developing late AMD^151^, ^152^, ^153^ . Serum free thyroxine (FT4) levels are associated with increased risk of developing AMD (FT4 increase per pmol L^−1^, HR 1.04)^153^, while serum thyroid‐stimulating hormone (TSH) is not.^151^, ^153^ It is suggested that hyperthyroidism can accelerate the basal metabolic rate and oxidative metabolism by induction of mitochondrial enzymes, which may induce oxidative stress.^154^ Also, there is evidence that thyroid hormones may adversely influence RPE cells, causing degeneration of photoreceptors.^155^, ^156^

#### Diabetes

Diabetes is a major concern in ophthalmic care. Whether it also contributes to the development of AMD remains unclear. Several studies present positive correlations between diabetes and the development of AMD,^77^, ^95^ but most studies show no effect.^2^, ^94^, ^108^, ^111^, ^115^, ^146^ Hyperglycemia and dyslipidemia in diabetic patients is thought to disturb the homeostasis of the retina by inducing inflammatory responses in retinal tissue, including oxidative stress.^157^ However, diabetes itself is probably not a strong predictor for the development of AMD.

#### Alzheimer's disease (AD)

Studies have found several clinical and pathologic similarities between AD and AMD. First of all, ageing is a major risk factor for both multifactorial degenerative disorders.^158^ Secondly, extracellular amyloid β‐peptide deposition, the primary pathologic hallmark of AD, is also a component of drusen in AMD.^159^ Furthermore, both diseases involve several isoforms encoded by different alleles of the apolipoprotein E (*APOE*) gene. Interestingly, while the APOE Ԑ2 and Ԑ4 isoforms, encoded by two *APOE* alleles, are associated with a decreased and increased risk for AD respectively, opposite directions are reported for AMD (see section on lipid metabolism genes).^160^, ^161^ In literature, the association between AD and AMD remains inconclusive.^162^, ^163^ Even if AD and AMD share some similarities in immune and inflammatory degenerative mechanisms, these diseases are probably not directly associated.

#### Parkinson’s disease (PD)

Parkinson’s disease is a progressive neurodegenerative disorder that results in the loss of dopaminergic neurons in the substantia nigra and causes motor abnormalities including bradykinesia, resting tremor and imbalance. A recent epidemiologic study showed that patients with PD treated with levodopa less often developed AMD (OR 0.78) and at a later age (mean age of onset 79 years) compared to untreated patients (mean age of onset 71 years).^164^ Levodopa is shown to stimulate the GRP143 receptor which decreases the release of VEGF and exosomes with inflammatory factors from RPE cells.^165^ Currently, clinical trials are investigating whether treatment with levodopa may indeed reduce the inflammatory reaction of the retina and slow down disease progression in AMD. Although it is not likely that PD and AMD are directly associated, both neurodegenerative diseases share probably some underlying pathophysiological features.

## Genetic risk factors

Genetic factors are known to play an important role in the development of AMD and genetic studies have contributed significantly to our knowledge on AMD pathology. To date, more than half of the genomic heritability can be explained by both common and rare genetic variants in 34 genetic loci, which are mostly involved in the complement system, extracellular matrix remodeling, and lipid metabolism.^166^ *Table* 1 and Table S3 show an overview of the top hits in the 34 AMD associated loci, as identified in the original Genome Wide Association Study (GWAS) of 2016. Variants of other studies were also included when they were in high linkage disequilibrium (LD) with the AMD‐associated variants of the original discovery GWAS, as referred in the subscript. Of all discovered AMD‐associated variants, a few have also been associated with disease progression in a recent GWAS on progression to late stage AMD,^167^ and in several prospective cohort studies.

**Table 1:** Summary overview of the top hits in the 34 age‐related macular degeneration (AMD) associated loci, as identified in the case‐control Genome Wide Association Study (GWAS) from the International AMD Genomics Consortium, and their association with disease progression as described in the GWAS of the Age‐Related Eye Disease Study (AREDS), and several prospective cohort studies

		Case‐control GWAS‡ ^,^ 166	GWAS on progression§ ^,^ 167	Prospective studies on progression¶			
		Late AMD	Late AMD	Early AMD	nAMD	GA	GA growth
Range of follow‐up for included studies (years)		–	10	3–15	2–10	5–20	4–5
Locus name	Index variant						
*CFH*	rs10922109†	↓	–	–	↓	–	–
*CFH*	rs570618†	↑	↑	↑	↑	↑	–
*COL4A3*	rs11884770	↓	–	–	–	–	–
*ADAMTS9‐AS2*	rs62247658†	↑	–	–	–	–	–
*COL8A1*	rs140647181	↑	–	–	–	–	–
*CFI*	rs10033900	↑	–	–	–	↑	–
*C9*	rs62358361	↑	–	–	–	–	–
*PRLR/SPEF2*	rs114092250	↓	–	–	–	–	–
*C2/CFB/SKIV2L*	rs116503776†	↓	↓	–	↓	–	–
*VEGFA*	rs943080	↓	–	–	–	–	–
*KMT2E/SRPK2*	rs1142	↑	–	–	–	–	–
*PILRB/PILRA*	rs7803454	↑	–	–	–	–	–
*TNFRSF10A*	rs79037040	↓	–	–	–	–	–
*MIR6130/RORB*	rs10781182	↑	–	–	–	–	–
*TRPM3*	rs71507014	↑	–	–	–	–	–
*TGFBR1*	rs1626340†	↓	–	–	–	–	–
*ABCA1*	rs2740488†	↓	–	↓	–	–	–
*ARHGAP21*	rs12357257	↑	–	–	–	–	–
*ARMS2/HTRA1*	rs3750846†	↑	↑	↑	↑	↑	↑
*RDH5/CD63*	rs3138141	↑	–	–	–	–	–
*ACAD10*	rs61941274	↑	–	–	↑	–	–
*B3GALTL*	rs9564692	↓	–	–	–	–	–
*RAD51*	rs61985136†	↓	–	–	↓	–	–
*LIPC*	rs2043085	↓	–	–	↓	↓	–
*CETP*	rs5817082†	↓	–	↓	–	–	–
*CTRB2/CTRB1*	rs72802342	↓	–	–	↓	↓	–
*TMEM97/VTN*	rs11080055	↓	–	–	–	–	–
*NPLOC4/TSPAN10*	rs6565597	↑	–	–	–	–	–
*C3*	rs2230199	↑	↑	–	↑	↑	↓
*CNN2*	rs67538026	↓	–	–	–	–	–
*APOE*	rs429358	↓	–	–	–	–	–
*MMP9*	rs142450006	↓	–	–	↓	–	–
*C20orf85*	rs201459901	↓	–	–	–	–	–
*SYN3/TIMP3*	rs5754227	↓	–	–	–	–	–
*SLC16A8*	rs8135665	↑	–	–	–	–	–

↑, minor allele is a risk factor; ↓, minor allele is a protective factor; AMD, age‐related macular degeneration; AREDS, Age‐Related Eye Disease Study; GA, Geographic atrophy; GWAS, Genome Wide Association Study; nAMD, neovascular age‐related macular degeneration.

^†^ The following variants were also included since they are in high LD (*R* ^2^ > 0.80) with the AMD‐associated variants of the original discovery GWAS of 2016: *ADAMTS9‐AS2:* rs6795735 for rs62247658 (*R* ^2^ = 0.984), *ARMS2/HTRA1:* rs10490924 for rs3750846 (*R* ^2^ = 1.0), *C2/CFB/SKIV2L:* rs429608 for rs116503776 (*R* ^2^ = 1.0), *CFH*: rs1410996 for rs10922109 (*R* ^2^ = 1.0), *CFH*: rs1061170 for rs570618 (*R* ^2^ = 1.0), *RAD51B*: rs8017304 for rs61985136 (*R* ^2^ = 1.0), *TGFBR1*: rs334353 for rs1626340 (*R* ^2^ = 0.855), *CETP*: rs1864163 for rs5817082 (*R* ^2^ = 0.975), *ABCA1*: rs1883025 for rs2740488 (*R* ^2^ = 0.941).

^‡^ Original discovery GWAS, based on case‐control study from the International AMD Genomics Consortium using 33 976 participants (no follow‐up).

^§^ GWAS on AMD progression, based on a prospective data from the AREDS study using 2721 participants (10 years follow‐up).

^¶^ Table S3 provides detailed information on the prospective studies included in this overview.

### Complement genes

In 2005, several research groups identified a common variant (rs1061170) in the complement factor H (*CFH*) gene which had a strong effect on the risk of AMD. This variant is in perfect LD (*R* ^2^ = 1.0) with the rs570618 variant in the original discovery GWAS of 2016. Multiple prospective studies have confirmed that the minor allele of this variant is a risk allele for the incidence of early AMD (HR 1.2–2.3),^40^, ^92^, ^107^, ^168^, ^169^ as well as for the progression to nAMD (HR 1.48–2.5)^92^, ^170^ and GA (HR 1.38–3.65).^23^, ^86^, ^92^, ^171^ Another common variant in the *CFH* gene is the rs10922109 variant, of which the minor allele is confirmed to be a protective allele for the progression to late stage AMD (HR 0.43).^167^, ^170^ However, both variants in the *CFH* gene have not yet been confirmed to be also associated with a more rapid GA enlargement in two prospective studies.^63^, ^172^

Variants in other complement loci, including *C2/CFB*, *CFI,* and *C3* have also found to be associated with disease progression in several studies. The minor allele of the rs116503776 variant in *C2/CFB* is a protective allele for disease progression to late stage AMD (HR 0.56)^167^, ^173^; the minor allele of the rs10033900 variant in *CFI* is a risk allele for AMD, but has only been confirmed to be associated with disease progression to GA (HR 1.19)^92^; and the minor allele of the rs2230199 variant in *C3* is a risk allele for AMD and confirmed to be a risk factor for the incidence of early AMD (HR 1.22),^92^ and progression to nAMD (HR 1.25)^92^ and GA (HR 1.26).^86^, ^92^ Interestingly, when GA had developed, the minor allele of the rs2230199 variant in *C3* was a protective factor for GA growth (the wildtype, heterozygous and homozygous combination had progression rates of 0.30, 0.27 and 0.15 mm year^−1^, respectively), which was later confirmed in another cohort.^63^, ^172^ The authors hypothesised that AMD‐associated variants may have differential effects at different disease stages.

### The *ARMS2/HTRA1* genes

The common rs10490924 variant near the *Age‐related maculopathy susceptibility 2* and *high‐temperature requirement A serine peptidase 1* (*ARMS2*/*HTRA1*) genes has also a strong effect on the risk of AMD and was also identified in the first genetic AMD studies. This common variant is in perfect LD (*R* ^2^ = 1.0) with the rs3750846 variant in the original discovery GWAS of 2016. Several prospective studies have confirmed that the minor allele of this variant is a risk factor for the incidence of early AMD (OR 1.36), 40, 92 and progression to nAMD (HR 1.4–2.8) 92, 170, 171, 173 and GA (HR 1.4–3.3), 23, 79, 86, 92, 170, 171 and increased GA growth (the wildtype, heterozygous and homozygous combination had progression rates of 0.23, 0.30 and 0.32 mm year^−1^, respectively).^63^, ^172^ Functional studies suggest that the *ARMS2* gene may encode a protein which has a function in the mitochondrial outer membrane of the retina, while other reports suggest that it encodes an extracellular protein.^174^, ^175^ The *HTRA1* gene encodes a heat shock serine protease and regulates TGF‐β signalling, which is involved in regulating extracellular matrix deposition and angiogenesis.^176^, ^177^ However, the exact function of the *ARMS2/HTRA1* genes and how either or both genes are related to the pathophysiology of AMD is still not fully understood.

### Lipid metabolism genes

Genes encoding proteins of the lipid metabolism are also involved in the pathogenesis of AMD, and are probably involved in the formation of drusen.^178^ Some of the AMD‐associated variants in lipid metabolism genes found in the case‐control GWAS of 2016 are also confirmed with disease progression. For example, the minor allele of the rs2740488 variant in *ABCA1* and minor allele of the rs5817082 variant in *CETP* are both confirmed to be protective alleles for the incidence of early AMD (OR 0.77–0.82, and OR 0.87 respectively). 92, 168, 179 In the *LIPC* gene, the minor allele of the rs2043085 variant, is a protective allele for the progression towards late AMD (HR 0.83).^92^, ^167^

Another gene involved in lipid metabolism is the *APOE* gene, which is known to be polymorphic. Variants in the *APOE* gene at amino acid positions 112 (rs429358) and 158 (rs7412) determine three major isoforms: the Ԑ2 isoform (112C, 158C), the most common Ԑ3 isoform (112C, 158R) and the Ԑ4 isoform (112R, 158R).

Studies show that the *APOE* Ԑ4 isoform is associated with an increased risk of Alzheimer’s disease and the *APOE* Ԑ2 isoform is associated with a decreased risk of Alzheimer’s disease. Interestingly these two isoforms have an opposite effect in AMD: the *APOE* Ԑ4 isoform is associated with a decreased risk of AMD, and there is a trend for the *APOE* Ԑ2 isoform to be associated with an increased risk of developing AMD. 160, 161 However, these *APOE* isoforms and other AMD‐associated variants in the *APOE* gene have not yet been investigated with AMD disease progression in prospective cohort studies.

### Other genes

Other variants that were identified in the original discovery GWAS of 2016, and confirmed to be associated with disease progression in AMD include rs61985136 in *RAD541B* and rs72802342 in *CTRB2/CTRB1*.^167^, ^170^ Interestingly, a recent GWAS on disease progression in AMD showed that the minor allele of the rs142450006 variant near *MMP9* was associated with progression to nAMD (HR 0.66) but not with GA.^167^ This finding was also found previously in the original discovery GWAS.^166^ In addition, the rs58978565 variant near *TNR* and the rs28368872 variant near *ATF7IP2* are associated with disease progression to nAMD but not GA.^167^ These variants were not previously found in the original discovery GWAS. Larger genome‐wide association studies with prospective data are needed to investigate whether some variants are mainly involved in a specific AMD disease stage (early AMD, nAMD or GA) and whether variants can influence the progression speed of the disease.

### Rare variants

Several studies support a role for rare variants in the pathogenesis of AMD.^166^ It has been suggested that AMD in densely affected families are likely to be caused by rare, highly penetrant variants in genes of the complement system as *C3*, *C9*, *CFH,* and *CFI*.^180^ Since some of these rare variants have been associated with an earlier age of onset, patients carrying such variants may develop late AMD at an earlier age, causing many more years of substantial vision loss.^181^, ^182^ For an accurate risk assessment, it is therefore important that genetic tests for AMD are designed to also detect rare coding variants in these genes. However, there are currently no prospective cohort studies investigating the association between rare variants and disease progression in AMD.

## Molecular risk factors

### Angiogenic factors

Angiogenic factors, including vascular endothelial growth factor (VEGF), pigment epithelium derived factor (PEDF) and transforming growth factor beta (TGF‐β) have been extensively investigated in case‐control studies.^183^ VEGF is a hypoxia‐driven signal which induces the formation of new blood vessels and is currently the most important target in the treatment of nAMD to restore and/or maintain vision. PEDF is produced by RPE cells and has anti‐angiogenic properties, opposing the effects of VEGF, and supplementing with PEDF has been proposed for the treatment of nAMD.^184^ TGF‐β is found to increase the expression of VEGF and is therefore also implicated as an angiogenic factor.^185^ Cross‐sectional studies found conflicting results whether measurements of systemic and local angiogenic factors differ between AMD patients and controls.^183^ Currently, there are no prospective cohorts that have measured angiogenic factors to predict disease progression in AMD.

### Oxidative stress factors

Increased oxidative stress is thought to be one of the underlying factors in the occurrence of AMD.

Oxidative stress reflects an imbalance between the manifestation of reactive oxygen species (ROS) and reactive nitrogen species (RNS) that damage components of cells, and the biological ability to detoxify these reactive intermediates to maintain oxidant homeostasis.^186^ The eye, especially the macula, is susceptible to oxidative stress because of its high metabolic activity and its high content of polyunsaturated fatty acids (PUFAs) in photoreceptors, which are prone to oxidation. Several case‐control studies have found evidence of elevated products of oxidative stress in AMD patients,^183^ however prospective studies investigating a correlation with AMD progression are limited.

Malondialdehyde (MDA) is a reactive carbonyl compound that originates from the oxidation of PUFAs. Case‐control studies have consistently observed increased systemic levels of MDA in AMD patients compared to controls.^183^ However, there are currently no prospective studies that have investigated MDA levels and disease progression in AMD.

Another product of oxidative stress is the oxidation of low‐density lipoproteins (LDL) forming oxidized LDL (ox‐LDL). LDL are lipoproteins (see section Lipoproteins) that are an easy target for oxidation by free radicals. While some cross‐sectional studies show increased levels of systemic ox‐LDL in AMD patients,^183^ only two prospective cohorts have investigated the relationship between systemic ox‐LDL and disease progression and found no correlation.^83^, ^146^

Several case‐control studies show a relation between AMD and toxic trace elements, such as cadmium, lead, and mercury.^183^ These toxic trace‐elements are thought to induce oxidative stress and the production of inflammatory cytokines. In addition, some trace‐elements share numerous binding sites with other anti‐oxidative trace‐elements such as zinc (see section Zinc). Of all toxic trace‐elements, cadmium has drawn considerable attention since some studies showed that cadmium levels may be related to AMD pathogenesis, especially in the smoking population.^187^ However, there are currently no prospective studies investigating the potential usage of measuring toxic trace‐elements to predict disease progression in AMD.

### Anti‐oxidative factors

Antioxidants, including vitamins, carotenoids and essential trace elements such as zinc, both enhance the clearance and prevent the formation of ROS and RNS, reducing the damage in retinal cells caused by oxidative stress.^188^ Several studies hypothesise that the antioxidant capacity in AMD patients might be impaired, and a decreased antioxidant capacity might be associated with both disease onset and progression. Table S4 shows an overview of all prospective studies analysing systemic anti‐oxidative factors and their association with the development of early and late AMD. Of note, most prospective studies investigating vitamins and carotenoids have estimated the systemic levels based on food frequency questionnaires and only a minority of the studies have measured systemic blood levels.^189^, ^190^

#### Vitamins

Vitamin C protects against oxidative stress‐induced cellular damage by scavenging ROS and mediating reactivation of vitamin E. The idea that vitamin C may be protective was primarily based on a series of animal studies, which showed an association between vitamin C and a reduction of light‐induced damage in rat retinas.^191^ However, most prospective studies investigating the relationship between dietary intake of vitamin C and the onset and progression of AMD have shown no association.^109^, ^192^, ^193^, ^194^ Surprisingly, one longitudinal study even suggested that higher dietary vitamin C intake and supplementation could increase the risk for developing early AMD.^195^ The investigators could not explain this finding on biological grounds, nor attribute it to a measurement error or bias. Furthermore, one longitudinal population cohort measured systemic vitamin C levels and found no association with the incidence of AMD over two year follow‐up.^189^ Thus, although vitamin C is a potent antioxidant, results of prospective studies are not conclusive whether vitamin C status alone is also associated with the onset or progression of AMD.

Vitamin E is a lipophilic antioxidant in the plasma membrane of RPE cells and photoreceptors, and prevents lipid peroxidation. Prospective cohorts investigating the relationship between dietary intake of vitamin E and the risk of AMD are inconsistent. Two longitudinal population studies show a reduced risk of developing AMD when consuming a higher dietary intake of vitamin E,^192^, ^194^ while two other studies found no association.^109^, ^193^ One study even found an increased risk for GA in those consuming higher amounts of dietary vitamin E.^196^ Currently, there is only one longitudinal population study which measured systemic levels of vitamin E. This study showed that higher systemic levels of vitamin E reduce the risk of developing AMD over two years.^189^ Further studies are needed to elucidate the role of vitamin E in AMD progression.

Vitamin A is a cofactor in photoreceptors to form rhodopsin, which is a protein involved in the visual phototransduction. Dietary intake of vitamin A has not been associated with the onset of AMD in four longitudinal population studies.^192^, ^194^, ^195^, ^196^ Also systemic levels of vitamin A showed no relation with the development of AMD over two years in one longitudinal population study.^189^ However, precursors of vitamin A, such as carotenes (see section Carotenoids) are more often investigated.

B vitamins, such as vitamin B9 (folate) and B12 (cobalamin) are essential cofactors to convert homocysteine into methionine. Homocysteine is an amino acid that is susceptible to oxidation and thought to induce vascular endothelial dysfunction in nAMD.^197^ There is only one longitudinal population study that investigated the association of dietary vitamin B9 and vitamin B12 intake with the development of GA, and found no association.^86^

Vitamin D can be produced in the dermis upon sunlight exposure or obtained through diet. There has been only one longitudinal population study regarding the role of vitamin D in AMD. This study found a decreased risk of developing nAMD when consuming a diet rich in vitamin D.^87^ However since the two most common dietary sources of vitamin D are milk and fish, it is possible that the association was more driven by the presence of omega‐3 fatty acids in the fish, rather than vitamin D.

#### Carotenoids

Carotenoids are organic pigments that are synthesised in plants and can be subdivided into carotenes and xanthophylls. Because humans are unable to synthesise carotenoids, they must be obtained through dietary consumption. The proposed protective mechanism of carotenoids for AMD is their ability to have an antioxidative effect through the absorptions of free electrons from ROS. Six carotenoids are commonly found in the human diet: α‐carotene, β‐carotene, β‐cryptoxanthin, lycopene, lutein and zeaxanthin.

α‐carotene and β‐carotene are both vitamin A precursors and have antioxidative properties. Both carotenes can be found in dark leafy vegetables such as spinach and kale, and yellow/orange vegetables such as carrots and bell peppers.

β‐carotene was added in the first supplement formula of the Age‐Related Eye Disease Study 1 (AREDS1) study (together with zinc, copper, vitamin C, and vitamin E) to investigate its properties to alter disease progression to central GA or neovascular AMD.^198^

After five years follow‐up, 20% of patients with supplementation progressed to late stage AMD, while 28% of the patients with placebo progressed to late stage AMD. This risk reduction was only found in patients with at least intermediate stage AMD.

Other clinical trials with high‐dose β‐carotene supplementation reported an increased risk of lung cancer in smokers, which raised concerns regarding the prescription of the supplements according to the formula of the AREDS1 study in AMD patients, since a large proportion of the AMD population are smokers.^199^, ^200^ Therefore, supplementation according to the formula of the AREDS1 study became only a recommendation in non‐smokers with intermediate stage AMD.

Longitudinal population studies reported conflicting results between dietary α‐carotene and β‐carotene levels and AMD onset and disease progression. Higher levels of α‐carotene and β‐carotene were associated with decreased risk of early and late AMD in some studies,^194^, ^201^, ^202^ but most studies did not find any association.^189^, ^192^, ^194^, ^195^ Two studies reported even an increased risk of developing late AMD in those consuming higher levels of dietary β‐carotene.^196^, ^203^ Thus, the protective effect of β‐carotene (and α‐carotene) levels in AMD progression is not conclusive.

β‐cryptoxanthin is also a vitamin A precursor, closely related to β‐carotene and commonly found in the rinds of oranges, but also inside papaya and apples. Only one longitudinal population study found a protective effect of adhering to a β‐cryptoxanthin diet for developing late AMD,^201^ while three other studies did not find any association.^192^, ^194^, ^195^

Lycopene is a red carotenoid found in tomatoes and bell peppers and has an antioxidative effect but no vitamin A activity. While some case‐control studies show lycopene levels are lower in patients with AMD compared to controls,^183^ longitudinal studies have not yet confirmed the protective effect of both systemic or dietary lycopene intake for the development and progression of AMD.^192^, ^194^, ^195^, ^201^

Lutein and zeaxanthin are two carotenoids that are found in the macular pigment located in the ganglion cells, cone axons and Müller cells of the macula. Both carotenoids have antioxidative properties and a protective effect by absorbing hazardous blue and ultraviolet light before it can reach the photoreceptors.^204^ Foods rich in lutein and zeaxanthin are mainly dark‐leafy vegetables (spinach, kale) and yellow/orange vegetables (carrots, bell peppers).

The main aim of the AREDS2 study was to improve the supplement formula of the AREDS1 study by exploring the additional effect of lutein, zeaxanthin, docosahexaenoic acid (DHA) and eicosapentaenoic acid (EPA) to the formula, and to evaluate the effect of eliminating β‐carotene and lowering zinc doses.^205^, ^206^

There was no difference in progression to late AMD in patients who used the AREDS supplements with lutein and zeaxanthin, compared with patients who only used the AREDS supplements. A subgroup analysis, however, did reveal a significant reduction in progression to late stage AMD in patients using the AREDS supplements with lutein and zeaxanthin and no β‐carotene, compared with patients using the AREDS supplements with β‐carotene (HR 0.82).^205^

Several studies have shown a link between supplementation of lutein and zeaxanthin and higher systemic levels.^207^, ^208^ However, inconsistent findings have been reported on the relation between the systemic lutein and zeaxanthin concentrations and the macular pigment optical density (MPOD), which is a measurement of the amount of local macular pigment.^207^, ^208^, ^209^

Longitudinal population studies are also not conclusive whether dietary lutein and zeaxanthin levels decrease disease progression. Half of these studies confirmed the protective effect of consuming higher dietary levels of lutein and zeaxanthin for the incidence of early AMD and progression to late AMD.^40^, ^196^, ^201^, ^202^ Others found no association for either lutein or zeaxanthin,^109^, ^190^, ^192^, ^194^, ^195^ and one study even reported an opposite effect.^210^ These conflicting results may partly be caused by the complex bioavailability of lutein and zeaxanthin caused by underlying pharmacogenomics, which makes the effect of lutein and zeaxanthin on disease progression more complex. Further prospective studies are needed to investigate whether the measurement of systemic lutein and zeaxanthin levels is useful to predict disease progression.

#### Zinc

Zinc is an essential trace element and plays a key role in many cellular processes such as DNA synthesis, RNA transcription, cell division, and prevention of cell apoptosis. In addition, zinc is essential for the development and maintenance of the immune system, including complement activation.^211^ In cells, the correct amount of zinc is regulated tightly by zinc transporters and storage proteins such as metallothioneins (MTs), which have antioxidative properties. There is evidence that due to ageing and oxidative stress the amount of MTs in the macula, and especially in the fovea, declines which triggers the release of zinc from MTs into the extracellular space where drusen formation occurs.^212^ Intracellular zinc depletion may also impair cellular metal homeostasis, induce apoptosis of RPE and retinal cells, and enhance oxidative stress and cell damage, causing the development of AMD.^213^ It is thought that zinc supplementation might trigger re‐uptake of zinc into the RPE‐choroid complex and increase the synthesis of MT, which helps to maintain retinal functions.^212^

The AREDS1 trial showed that patients using zinc supplements (80 mg zinc oxide) have a lower progression rate to late AMD after six years when compared to controls (HR 0.71).^198^ In the AREDS2 trial, both the original zinc doses (80 mg) and lower‐dose zinc (25 mg) were associated with the same progression rate to late stage AMD, which suggested that lower‐dose zinc can also be used to alter disease progression.^206^

Many longitudinal population studies show a protective effect of high dietary zinc intake for early and late AMD.^192^, ^194^, ^196^, ^202^ Interestingly, in an additional interaction analysis of the AREDS trial, the effectiveness of zinc appeared to differ by genotype.^171^ Currently, there are no studies on systemic zinc levels to predict disease progression. This is partly because systemic zinc levels in serum and plasma do not reflect zinc concentrations in cells, but also because systemic levels are influenced by many factors including age, sex and especially fasting.^214^

### Immune factors

#### Complement pathway

The complement system is a key component of innate immunity. It protects the host from invading microorganisms (e.g. bacteria, viruses, and fungi) by providing inflammatory signals and eliminating pathogens and infected cells, but it also contributes to the homeostasis of the host by the clearance of apoptotic cells and cellular debris.^215^ Activation of the complement system can be initiated through three pathways: the classical pathway, alternative pathway, and the lectin pathway. In the eye, there is especially a continuous low‐grade activation of the alternative pathway.^216^ Via this pathway, complement activity is initiated by conversion of complement component 3, which leads to a cascade of conversions of inactive proteins to their active forms. Subsequently, convertases (C3‐ and C5‐ convertase) and anaphylatoxins are produced, which can initiate a feedback loop resulting in an exponential increase of activated complement components. Finally, the alternative pathway cascade results in the formation of the membrane attack complex, which causes cell lysis. To avoid tissue damage, complement activation is closely regulated by several complement inhibitory proteins, such as complement factor I (CFI) and complement factor H (CFH).^217^

As mentioned in the section on complement genes, AMD is associated with many variants in genes encoding components and regulators of the complement system. Although the complement system acts locally in AMD, its components can also be measured systemically. Many case‐control studies have found differences in expression levels of systemic complement regulators and components between AMD patients and controls.^183^ However, it is not clear if systemic activation levels influence and/or reflect local complement activation in AMD. Currently, there are no prospective data on complement activation levels to predict disease progression, but, some studies have studied other systemic immune factors with disease progression (Table S5).

#### Cytokines

Cytokines are a broad category of small proteins that are important in cell signalling and can be subdivided into interleukins, tumor necrosis factor, chemokines, and interferons. There are currently only a few studies that have investigated the predictive value of systemic interleukin and tumor necrosis factor levels for disease progression in AMD, possibly because previous case‐control studies did not find differences in systemic chemokine and interferon levels between AMD patients and controls.^183^

Interleukins are a group of cytokines that are expressed by leukocytes and influence the function of the immune system. Interleukin 6 (IL‐6) is a cytokine produced by macrophages and T‐cells. Three prospective studies reported that systemic IL‐6 levels are positively correlated with the development of early AMD,^218^ progression to late AMD,^219^ and faster GA growth.^220^ Other interleukins, including IL‐1 β, IL‐8, and IL‐10, have also been studied in AMD, though to a lesser extent, and no relation has been found between these other interleukins and disease progression in AMD.^220^

Tumor necrosis factor–α (TNF‐α) is a cytokine involved in cell activation, differentiation, and apoptosis and its receptor, TNF‐αR2, is expressed on the choroidal vascular cells, RPE, and on Müller cells in the retina. One longitudinal population study found a positive relation between TNF‐αR2 levels and early AMD development.^218^ However, other studies found no association with the development of late AMD or GA growth.^219^, ^220^

#### C‐reactive protein

C‐reactive protein (CRP) is a protein of hepatic origin that increases following IL‐6 secretion. CRP activates the complement system through promoting phagocytosis by macrophages. Evidence of studies using CRP values to predict disease progression in AMD are inconclusive. Five prospective studies found no relation between systemic CRP levels and AMD progression.^88^, ^93^, ^94^, ^219^, ^220^ There is one study which used a more precise measurement of CRP (high‐sensitivity CRP [hsCRP]) and found a positive relationship with the development of early AMD.^218^

#### Intercellular‐ and Vascular Cell Adhesion Molecule 1

Intercellular adhesion molecule (ICAM) and vascular cell adhesion molecule (VCAM) regulate inflammation by attracting white blood cells and controlling their migration into the blood vessel wall. Complement‐mediated activation of choroidal endothelial secretion of ICAM and VCAM has been hypothesised to play a role in the pathogenesis of AMD. Two longitudinal population studies showed no association between systemic ICAM levels and disease progression to early AMD and late AMD.^218^, ^219^ However, one of these studies did find a positive relation between systemic VCAM levels and the development of early AMD.^218^

#### White blood cell count

As discussed in the section on complement genes**,** genetic variants in complement genes are related to AMD, thus the immune system is linked to the pathogenesis of AMD. The increase of several immune components also causes cellular proliferation of immune cells increasing the white blood cell (WBC) count. A relatively large number of studies have investigated the WBC count and the progression of AMD but did not detect any association.^2^, ^40^, ^88^, ^218^ Nevertheless, WBCs may still be considered a potential risk factor for AMD progression since it is conceivable that it is not the total number of cells that change, but rather the ratio between different cell types.^183^

### Lipid factors

Both case‐control studies and prospective studies show associations between several lipid factors and AMD.^183^ Table S6 shows an overview of different kinds of systemic lipid factors and their association with the development of early and late AMD, as identified in prospective cohort studies. Of note, most prospective studies investigating fatty acids have estimated the systemic levels based on food frequency questionnaires and only a minority of the studies have measured systemic blood levels.^127^, ^221^, ^222^, ^223^, ^224^

#### Lipids

Cholesterol is an organic molecule biosynthesised by cells or ingested with the diet, and is an essential structural component of cell membranes, involved in several cell signalling processes, and a precursor for the biosynthesis of steroid hormones, bile acid, and vitamin D. The vast majority of studies did not find any relationship between systemic cholesterol levels and progression to early AMD, GA or nAMD.^40^, ^88^, ^93^, ^94^, ^108^, ^114^, ^115^, ^116^, ^146^, ^179^, ^225^ Only two studies found an association between serum cholesterol en the development of late stage AMD.^2^, ^111^ One of these studies reported that serum cholesterol levels have a protective effect on the development of nAMD (per 10 mg dL^−1^ cholesterol, OR 0.94), while they are a risk factor for the development of GA (per 10 mg dL^−1^ cholesterol, OR 1.08) over five years follow‐up.^2^

Triglycerides are molecules consisting of glycerol bound to three fatty acids of variable length. Triglycerides are the main constituents of body fat and are also present in the blood to enable the transference of adipose fat and blood glucose. Most studies do not find a relation between systemic triglyceride levels and disease progression in AMD.^40^, ^93^, ^108^, ^111^, ^115^, ^146^ There is one prospective study that found a decreased risk of developing any AMD after 18 years follow‐up in patients having higher baseline systemic triglyceride levels.^88^

#### Lipoproteins

Lipoproteins are biochemical proteins whose primary purpose is to transport hydrophobic lipids, such as cholesterol and triglycerides, through the circulation. Lipoproteins can be classified into five different lipoproteins, as based on their density and size: chylomicrons; very‐low‐density lipoprotein (VLDL); intermediate‐density lipoprotein (IDL); low‐density lipoprotein (LDL); and high‐density lipoprotein (HDL). Both LDL and HDL transport cholesterol between the liver and periphery and have been extensively studied for their association with the development of AMD and disease progression.

LDL‐cholesterol (LDL‐C) is the main carrier of cholesterol in the circulation. Five large longitudinal population studies have investigated the association between systemic LDL‐C levels and disease progression in AMD over different lengths of follow‐up ranging from two to 18 years. Nonetheless, none of these studies found an association with the development of early and late AMD.^88^, ^93^, ^108^, ^115^, ^146^

Longitudinal population studies evaluating systemic HDL‐C levels in AMD show mixed results. The majority of the studies report that patients with increased levels of HDL‐C have an increased risk of developing early and late AMD,^93^, ^94^, ^108^, ^116^, ^146^, ^179^, ^225^ the minority of the studies show no relation,^2^, ^21^, ^88^, ^111^ and one prospective study reported an opposite effect.^115^ Some studies suggest that the changes in HDL composition and functionality might be more important for the development of AMD, rather than the total systemic circulation levels itself.^178^ Furthermore, there is growing evidence that HDL is directly involved in complement regulation and is able to transport specific complement components and regulators such as C3, CFB, C5 and C2.^178^

#### Apolipoproteins

Apolipoproteins (apo) are structural proteins that bind triglycerides and cholesterol to form lipoproteins. During the development of AMD, it has been suggested that RPE cells secret Apo into the Bruch’s membrane, forming a lipid barrier that retards the transportation of oxygen, nutrition, and waste products, which stimulates drusen formation. There are two longitudinal studies investigating systemic apolipoproteins and the progression of AMD.^111^, ^219^ Only one of these studies found that systemic ApoA1 levels, the major structural protein component of HDL, are associated with an increased risk of developing late AMD, while systemic ApoB levels, the primary apolipoproteins of the other four lipoproteins, are associated with a decreased risk for developing early AMD over 14 years follow‐up.^111^

#### Fatty acids

Fatty acids consist of a carboxyl group with a hydrocarbon chain, which is either saturated or unsaturated. Fatty acids have different functions, including storage as triglycerides to be used as an energy substrate, the formation of cholesterol esters, and usage as structural components for cell membranes. Fatty acids are characterised by their number of carbon and double bonds. Saturated fatty acids (SFAs) have no double bond, monounsaturated fatty acids (MUFAs) have one double bond, and polyunsaturated fatty acids (PUFAs) have two or more double bonds. The latter can be subdivided into omega‐3 fatty acids and omega‐6 fatty acids, depending on the location of the first double bond. Studies suggest that metabolites derived from omega‐3, especially eicosapentaenoic acid (EPA) and docosahexaenoic acid (DHA), are anti‐inflammatory and antiangiogenic, whereas those derived from omega‐6 are pro‐inflammatory and pro‐angiogenic.

All longitudinal population studies which investigated dietary intake of SFAs, including palmitic acid and stearic acids, found no relation with the development of early AMD or progression to late stage AMD.^89^, ^123^, ^124^, ^125^, ^126^, ^210^, ^226^, ^227^

There are currently no longitudinal prospective studies that investigated systemic plasma or serum SFA levels with AMD progression.

Dietary intake of MUFAs, including oleic acids (OAs) and palmitoleic acids (PAs), have been extensively investigated for the development and progression of AMD. However, none of the longitudinal population studies found an association of dietary MUFA levels with the development of early AMD or progression to late stage AMD,^89^, ^123^, ^124^, ^125^, ^126^, ^210^, ^226^, ^227^ and there are currently no prospective studies that have evaluated plasma or serum MUFA levels on AMD progression.

Several dietary omega‐3 fatty acid levels have been investigated in AMD, including a‐linolenic acid (ALA), eicosapentaenoic acid (EPA) and docosahexaenoic acid (DHA). Omega‐3 fatty acids are in high quantities found in fatty fish, which is a diet component that has been confirmed to reduce the risk of AMD development in multiple studies (see section Diet).

Whether increased dietary intake or higher systemic levels of ALA are associated AMD progression is not clear. Three longitudinal population studies showed no association between a higher dietary intake of ALA and AMD progression,^124^, ^126^, ^226^ and three prospective studies on systemic ALA levels reported conflicting results: one study showed that higher plasma ALA levels were associated with a decreased risk of developing GA and nAMD,^221^ while two other studies showed an increased risk of developing early and late AMD.^123^, ^224^

Several longitudinal studies have investigated dietary intake and systemic levels of EPA and DHA in AMD. Half of these studies found a decreased risk of developing early or late AMD in patients consuming higher amounts of EPA,^202^, ^203^, ^228^ and the majority of longitudinal studies reported a decreased risk of developing early or late AMD when consuming higher amounts of DHA. 89, 123, 202, 203, 228 Only a few prospective studies reported no relation between dietary intake of EPA^89^, ^123^, ^226^ or DHA^226^ and AMD. Based on the results of these epidemiological studies, two clinical trials have been conducted to investigate the value of omega‐3 supplementation to delay disease progression.

The Age‐Related Eye Disease Study 2 (AREDS2), a double‐blind trial in AMD patients, found no decreased risk of AMD progression after five years follow‐up in patients using EPA and DHA supplementation in addition to the original supplement formulation of the AREDS1 study (zinc, copper, vitamin C, vitamin E, and β‐carotene) when compared to patients using the original supplement formulation of the AREDS1 study only.^205^ However, some investigators suggested that the design of the AREDS2 trial might not have permitted the prophylactic potential of omega‐3 to be demonstrated.^178^

Furthermore, the nutritional AMD Treatment 2 (NAT‐2) study, a double‐blind trial which investigated the effect of EPA and DHA supplementation in patients with early AMD in the study eye and neovascular AMD in the fellow eye, did also not find a decreased risk of nAMD development in the fellow‐eye after three years follow‐up.^223^ However, the investigators observed poor treatment compliance in the treatment group, demonstrated by a high variance in the systemic EPA/DHA levels in the red blood cell membrane. Interestingly, patients who maintained a consistently high EPA/DHA levels in the RBCM, and thus therapy compliance, had a reduced risk of developing late AMD in the fellow‐eye (HR 0.32). This finding is somewhat analogous to that observed in the majority of longitudinal population studies, which showed a reduced risk of AMD progression in patients with higher plasma or serum EPA and DHA levels.^127^, ^221^, ^222^ Therefore, systemic omega‐3 fatty acids levels, especially EPA and DHA measured in red blood cell membranes, seem to be a good indicator of dietary compliance and a systemic predictor of AMD progression.

Two kinds of omega‐6 fatty acids have been studied for disease progression in AMD: linoleic acid (LA) and arachidonic acid (AA). The majority of longitudinal studies found no association between dietary intake or systemic levels of LA or AA and AMD development.^89^, ^124^, ^126^, ^226^ Only one longitudinal population study reported an increased risk of developing AMD over 12 year follow‐up in patients with a high dietary intake of LA.^123^

## Personalised prediction of disease progression

### Current prediction models

For the development of personalised prediction models, accurate and easy quantification of risk factors is important. As discussed in the previous sections, numerous phenotypic, demographic, environmental, genetic and molecular risk factors have been described to be associated with disease progression in AMD. These risk factors can be combined in prediction models to predict disease progression, but the selection of the proper risk factors for personalised risk prediction will differ among individuals and may be dependent on their current disease stage. While phenotypic risk factors are more important to predict disease progression during the course of the disease, demographic, environmental, genetic and molecular risk factors may be more valuable at earlier disease stages. The prediction models developed to date include similar risk factors: phenotypic disease classification based on drusen and pigment abnormalities identified on CFP; demographic factors such as age and sex; environmental risk factors such as smoking and BMI; and a subset of AMD variants, including rs570618 in *CFH* and rs860846 in *ARMS2/HTRA1*.

*Table* 2 provides an overview of 25 developed prediction models to predict late stage AMD, as developed in eight different population studies with follow‐up data ranging between 3–12 years. All these population studies used CFPs for identifying phenotypical characteristics. When only demographic risk factors such as age and gender are included, the prediction of late stage AMD is poor (AUC 0.60–0.64). Adding environmental risk factors such as BMI and smoking results in a better predicting performance (AUC 0.62–0.67). Next, adding genetic information or phenotypic information further improves the predictive performance of the models (AUC 0.75–0.86 and AUC 0.89–0.90, respectively). Finally, when phenotypic, demographic, environmental and genetic risk factors are combined, prediction models reach the highest discriminative performances (AUC of 0.87–0.96).^91^, ^92^, ^229^, ^230^, ^231^, ^232^ Of note, the highest AUC was found in a prediction model that only considered central GA as late stage AMD, meaning that patients having non‐central GA at baseline were also included to predict central GA.^231^ This might explain the high discriminative performances of the models from this study compared to the prediction models from other studies, since patients with non‐central GA have a higher risk for progression to central GA compared to patients with intermediate stage AMD.

**Table 2:** Overview of prediction models for late stage age‐related macular degeneration (AMD), using combinations of risk factor categories (phenotypic, demographic, environmental, genetic and molecular) as developed in prospective cohort studies

References	Follow‐up (years)	Imaging modality	Definition progression to GA	Definition progression to nAMD	Phenotypic (P)	Demographic (D)	Environmental (E)	Genetic (G)	Molecular (M)	AUC
Buitendijk (2013)^239^	10	CFP	Central GA, non‐central GA	Retinal PED, subretinal fibrous tissue, hemorrhage, CNV membrane	–	D	–	–	–	0.60
Sardell (2016)^90^	3	CFP	Central GA, non‐central GA	Serous PED, subretinal fibrous tissue, hemorrhage, CNV membrane	–	D	–	–	–	0.64
Ding (2017)^229^	10	CFP	Central GA, non‐central GA	Retinal PED, haemorrhage, subretinal fibrous tissue	–	D	E	–	–	0.62
Perlee (2013)^231^	12	CFP	Central GA	Retinal PED, haemorrhage, subretinal fibrous tissue	–	D	E	–	–	0.63
Seddon (2015)^91^	10	CFP	Central GA, non‐central GA	Serous PED, subretinal fibrous tissue, hemorrhage, CNV membrane	–	D	E	–	–	0.67
Sardell (2016)^90^	3	CFP	Central GA, non‐central GA	Serous PED, subretinal fibrous tissue, hemorrhage, CNV membrane	–	D	–	G	–	0.67
Buitendijk (2013) 239	10	CFP	Central GA, non‐central GA	Retinal PED, subretinal fibrous tissue, hemorrhage, CNV membrane	–	D	–	G	–	0.82
Ding (2017)^229^	10	CFP	Central GA, non‐central GA	Retinal PED, haemorrhage, subretinal fibrous tissue	–	D	E	G	–	0.75
Seddon (2015)^91^	10	CFP	Central GA, non‐central GA	Serous PED, subretinal fibrous tissue, hemorrhage, CNV membrane	–	D	E	G	–	0.80
Perlee (2013)^231^	12	CFP	Central GA	Retinal PED, haemorrhage, subretinal fibrous tissue	–	D	E	G	–	0.86
Seddon (2015)^91^	10	CFP	Central GA, non‐central GA	Serous PED, subretinal fibrous tissue, hemorrhage, CNV membrane	–	–	–	G	–	0.79
Perlee (2013)^231^	12	CFP	Central GA	Retinal PED, haemorrhage, subretinal fibrous tissue	–	–	–	G	–	0.84
Ding (2017)^229^	10	CFP	Central GA, non‐central GA	Retinal PED, haemorrhage, subretinal fibrous tissue	P	–	–	–	–	0.88
Perlee (2013)^231^	12	CFP	Central GA	Retinal PED, haemorrhage, subretinal fibrous tissue	P	–	–	–	–	0.89
Ding (2017)^229^	10	CFP	Central GA, non‐central GA	Retinal PED, haemorrhage, subretinal fibrous tissue	P	D	E	–	–	0.89
Seddon (2015)^91^	10	CFP	Central GA, non‐central GA	Serous PED, subretinal fibrous tissue, hemorrhage, CNV membrane	P	D	E	–	–	0.90
Perlee (2013)^231^	12	CFP	Central GA	Retinal PED, haemorrhage, subretinal fibrous tissue	P	D	E	–	–	0.90
Seddon (2015)^91^	10	CFP	Central GA, non‐central GA	Serous PED, subretinal fibrous tissue, hemorrhage, CNV membrane	P	–	–	G	–	0.91
Perlee (2013)^231^	12	CFP	Central GA	Retinal PED, haemorrhage, subretinal fibrous tissue	P	–	–	G	–	0.96
Klein (2011)^230^	5	CFP	Central GA, non‐central GA	Retinal PED, haemorrhage, subretinal fibrous tissue	P	D	E	G	–	0.87
Buitendijk (2013)^232^	10	CFP	Central GA, non‐central GA	Retinal PED, subretinal fibrous tissue, hemorrhage, CNV membrane	P	D	E	G	–	0.88
Ding (2017)^229^	10	CFP	Central GA, non‐central GA	Retinal PED, haemorrhage, subretinal fibrous tissue	P	D	E	G	–	0.89
Yu (2012)^92^	10	CFP	Central GA, non‐central GA	Serous PED, subretinal fibrous tissue, hemorrhage, CNV membrane	P	D	E	G	–	0.90
Seddon (2015)^91^	10	CFP	Central GA, non‐central GA	Serous PED, subretinal fibrous tissue, hemorrhage, CNV membrane	P	D	E	G	–	0.91
Perlee (2013)^231^	12	CFP	Central GA	Retinal PED, haemorrhage, subretinal fibrous tissue	P	D	E	G	–	0.96

Phenotypic: AMD classifications based on drusen and pigment abnormalities; Demographic: age and sex; Environmental: smoking, BMI and in some studies level of education; Genetic: rs570618 or rs1061170 in *CFH*, rs3750846 or rs10490924 in *ARMS2/HTRA1*, and in some studies additional AMD variants; Molecular: there are currently no prospective studies using molecular risk factors in their prediction models.

AMD, age‐related macular degeneration; AUC, area under curve; CFP, colour fundus photography; CNV, choroid neovascularisation; D, demographic predictors; E, environmental predictors; G, genetic predictors; GA, Geographic Atrophy; M, molecular predictors; nAMD, neovascular age‐related macular degeneration; P, phenotypic predictors; PED, pigment epithelium detachment.

## Future prediction models

Despite a relatively high discriminative performance of the current prediction models for late stage AMD, they could be further improved by including other promising risk factors. Phenotypic risk factors in the current prediction models are all identified on CFP, while it is recommended to use a multimodal imaging approach to identify phenotypic risk factors.^233^ Using both CFP and OCT, additional features could be identified that are highly correlated with disease progression such as drusen types, hyperreflective foci, and pigment epithelial detachment. Additionally, OCTA can detect quiescent CNV/subclinical CNV, which may have a prognostic value for predicting exudative nAMD.^234^ However, this is currently still under investigation.

Secondly, the current prediction models only use a restricted set of genetic variants. For future development of prediction models and personalised prediction, using a wider set of AMD‐associated variants as discovered in the most recent GWAS^166^ may provide a more accurate genetic risk of individuals, and genetic risk scores can be calculated to establish the combined effect of all AMD‐associated genetic variants.^235^ In addition, rare variants should especially be taken into account in families with several members affected with AMD.^236^

Thirdly, current prediction models do not include molecular risk factors, while these factors can be measured quantitatively.^237^ Because some molecular risk factors are potentially modifiable, this could help to implement personalised preventive strategies for AMD. Promising systemic molecular factors that might be predictive for disease progression are HDL‐C, DHA, EPA, zeaxanthin, and lutein. However, current studies on these molecular factors show conflicting results, possibly because of a variable bioavailability of these factors caused by fluctuating systemic levels and underlying pharmacogenomics effects.^238^ Thus, before introducing these molecular factors into prediction models, more prospective research is needed to explore which factors influence the bioavailability of these molecular factors. When these molecular risk factors are confirmed in additional studies, including such molecular risk factors in addition to phenotypic, demographic, environmental and genetic risk factors may further improve the accuracy of prediction models, help to design preventive strategies based on modifiable risk factors, and provide personalised advice.

## Conflict of interest

The author(s) have no proprietary or commercial interest in any materials discussed in this article.

## Supporting information


**Table S1.** Overview of the association between phenotypic factors and the prediction to late AMD in prospective cohort studies
**Table S2.** Overview of the association between demographic and environmental factors and the prediction to early and late AMD in prospective cohort studies
**Table S3.** Overview of the top hits in the 34 AMD associated loci, as identified in the case‐control GWAS (Fritsche et al. 2016) from the International AMD Genomics Consortium, and their association with disease progression as described in the GWAS of the AREDS study (Yan et al. 2018), and several prospective cohort studies
**Table S4.** Overview of the association between anti‐oxidative factors and the prediction of early and late AMD in prospective cohort studies
**Table S5.** Overview of the association between immune factors and the prediction of early and late AMD in prospective cohort studies
**Table S6.** Overview of the association between lipid factors and the prediction of early and late AMD in prospective cohort studies

## References

[1] Wong WL , Su X , Li X , et al. Global prevalence of age‐related macular degeneration and disease burden projection for 2020 and 2040: a systematic review and meta‐analysis. Lancet Glob Health 2014; 2: e106–116. 25104651 10.1016/S2214-109X(13)70145-1

[2] Tomany SC , Wang JJ , Van Leeuwen R , et al. Risk factors for incident age‐related macular degeneration: pooled findings from 3 continents. Ophthalmology 2004; 111: 1280–1287. 15234127 10.1016/j.ophtha.2003.11.010

[3] Chakravarthy U , Wong TY , Fletcher A , et al. Clinical risk factors for age‐related macular degeneration: a systematic review and meta‐analysis. BMC Ophthalmol 2010; 10: 31. 21144031 10.1186/1471-2415-10-31 PMC3009619

[4] Schaal KB , Rosenfeld PJ , Gregori G , Yehoshua Z & Feuer WJ . Anatomic clinical trial endpoints for nonexudative age‐related macular degeneration. Ophthalmology 2016; 123: 1060–1079. 26952592 10.1016/j.ophtha.2016.01.034

[5] Schmitz‐Valckenberg S , Sadda S , Staurenghi G , et al. Geographic atrophy: Semantic considerations and literature review. Retina 2016; 36: 2250–2264. 27552292 10.1097/IAE.0000000000001258 PMC5115977

[6] Fleckenstein M , Mitchell P , Freund KB , et al. The progression of geographic atrophy secondary to age‐related macular degeneration. Ophthalmology 2018; 125: 369–390. 29110945 10.1016/j.ophtha.2017.08.038

[7] Ly A , Nivison‐Smith L , Assaad N & Kalloniatis M . Fundus autofluorescence in age‐related macular degeneration. Optom Vis Sci 2017; 94: 246–259. 27668639 10.1097/OPX.0000000000000997 PMC5287441

[8] Wu Z , Luu CD , Ayton LN , et al. Fundus autofluorescence characteristics of nascent geographic atrophy in age‐related macular degeneration. Invest Ophthalmol Vis Sci 2015; 56: 1546–1552. 25678689 10.1167/iovs.14-16211 PMC4349109

[9] Sadda SR , Guymer R , Holz FG , et al. Consensus definition for atrophy associated with age‐related macular degeneration on OCT: Classification of trophy Report 3. Ophthalmology 2018; 125: 537–548. 29103793 10.1016/j.ophtha.2017.09.028 PMC11366072

[10] Campochiaro PA , Soloway P , Ryan SJ & Miller JW . The pathogenesis of choroidal neovascularization in patients with age‐related macular degeneration. Mol Vis 1999; 5: 34. 10562658

[11] Treister AD , Nesper PL , Fayed AE , Gill MK , Mirza RG & Fawzi AA . Prevalence of subclinical CNV and choriocapillaris nonperfusion in fellow eyes of unilateral exudative AMD on OCT angiography. Transl Vis Sci Technol 2018; 7: 19. 10.1167/tvst.7.5.19 PMC6166896 30280004

[12] Klein R , Meuer SM , Myers CE , et al. Harmonizing the classification of age‐related macular degeneration in the three‐continent AMD consortium. Ophthalmic Epidemiol 2014; 21: 14–23. 24467558 10.3109/09286586.2013.867512 PMC4029416

[13] Ferris FL , Davis MD , Clemons TE , et al. A simplified severity scale for age‐related macular degeneration: AREDS Report No. 18. Arch Ophthalmol 2005; 123: 1570–1574. 16286620 10.1001/archopht.123.11.1570 PMC1473206

[14] Khan KN , Mahroo OA , Khan RS , et al. Differentiating drusen: Drusen and drusen‐like appearances associated with ageing, age‐related macular degeneration, inherited eye disease and other pathological processes. Prog Retin Eye Res 2016; 53: 70–106. 27173377 10.1016/j.preteyeres.2016.04.008

[15] Yehoshua Z , Wang F , Rosenfeld PJ , Penha FM , Feuer WJ & Gregori G . Natural history of drusen morphology in age‐related macular degeneration using spectral domain optical coherence tomography. Ophthalmology 2011; 118: 2434–2441. 21724264 10.1016/j.ophtha.2011.05.008 PMC3189426

[16] Schlanitz FG , Baumann B , Kundi M , et al. Drusen volume development over time and its relevance to the course of age‐related macular degeneration. Br J Ophthalmol 2017; 101: 198–203. 27044341 10.1136/bjophthalmol-2016-308422 PMC5293844

[17] Lei J , Balasubramanian S , Abdelfattah NS , Nittala MG & Sadda SR . Proposal of a simple optical coherence tomography‐based scoring system for progression of age‐related macular degeneration. Graefes Arch Clin Exp Ophthalmol 2017; 255: 1551–1558. 28534244 10.1007/s00417-017-3693-y

[18] Brader HS , Ying GS , Martin ER & Maguire MG . Complications of Age‐Related Macular Degeneration Prevention Trial Research G. Characteristics of incident geographic atrophy in the complications of age‐related macular degeneration prevention trial. Ophthalmology 2013; 120: 1871–1879. 23622873 10.1016/j.ophtha.2013.01.049 PMC3728180

[19] Ouyang Y , Heussen FM , Hariri A , Keane PA & Sadda SR . Optical coherence tomography‐based observation of the natural history of drusenoid lesion in eyes with dry age‐related macular degeneration. Ophthalmology 2013; 120: 2656–2665. 23830761 10.1016/j.ophtha.2013.05.029 PMC5340146

[20] Tikellis G , Robman LD , Dimitrov P , Nicolas C , McCarty CA & Guymer RH . Characteristics of progression of early age‐related macular degeneration: the cardiovascular health and age‐related maculopathy study. Eye (Lond) 2007; 21: 169–176. 16732219 10.1038/sj.eye.6702151

[21] Shim SH , Kim SG , Bae JH , Yu HG & Song SJ . Risk factors for progression of early age‐related macular degeneration in Koreans. Ophthalmic Epidemiol 2016; 23: 80–87. 26950426 10.3109/09286586.2015.1129425

[22] Nathoo NA , Or C , Young M , et al. Optical coherence tomography‐based measurement of drusen load predicts development of advanced age‐related macular degeneration. Am J Ophthalmol 2014; 158: 757–761. 24983793 10.1016/j.ajo.2014.06.021

[23] Joachim N , Mitchell P , Kifley A , Rochtchina E , Hong T & Wang JJ . Incidence and progression of geographic atrophy: observations from a population‐based cohort. Ophthalmology 2013; 120: 2042–2050. 23706948 10.1016/j.ophtha.2013.03.029

[24] Heesterbeek TJ , de Jong EK , Acar IE , et al. Genetic risk score has added value over initial clinical grading stage in predicting disease progression in age‐related macular degeneration. Sci Rep 2019; 9: 6611. 31036867 10.1038/s41598-019-43144-3 PMC6488669

[25] Folgar FA , Yuan EL , Sevilla MB , et al. Drusen volume and retinal pigment epithelium abnormal thinning volume predict 2‐year progression of age‐related macular degeneration. Ophthalmology 2016; 123: 39–50. 26578448 10.1016/j.ophtha.2015.09.016

[26] Abdelfattah NS , Zhang H , Boyer DS , et al. Drusen volume as a predictor of disease progression in patients with late age‐related macular degeneration in the fellow eye. Invest Ophthalmol Vis Sci 2016; 57: 1839–1846. 27082298 10.1167/iovs.15-18572

[27] Hallak JA , de Sisternes L , Osborne A , Yaspan B , Rubin DL & Leng T . Imaging, genetic, and demographic factors associated with conversion to neovascular age‐related macular degeneration: Secondary analysis of a randomized clinical trial. JAMA Ophthalmol 2019; 137: 738–744. 31021381 10.1001/jamaophthalmol.2019.0868 PMC6487912

[28] Spaide RF & Curcio CA . Drusen characterization with multimodal imaging. Retina 2010; 30: 1441–1454. 20924263 10.1097/IAE.0b013e3181ee5ce8 PMC2952278

[29] Ferris FL 3rd , Wilkinson CP , Bird A , et al. Clinical classification of age‐related macular degeneration. Ophthalmology 2013; 120: 844–851. 23332590 10.1016/j.ophtha.2012.10.036 PMC11551519

[30] Finger RP , Wu Z , Luu CD , et al. Reticular pseudodrusen: a risk factor for geographic atrophy in fellow eyes of individuals with unilateral choroidal neovascularization. Ophthalmology 2014; 121: 1252–1256. 24518615 10.1016/j.ophtha.2013.12.034 PMC4047161

[31] Connolly E , Rhatigan M , O'Halloran AM , et al. Prevalence of age‐related macular degeneration associated genetic risk factors and 4‐year progression data in the Irish population. Br J Ophthalmol 2018; 102: 1691–1695. 29453225 10.1136/bjophthalmol-2017-311673

[32] Sakurada Y , Sugiyama A , Kikushima W , et al. Pseudodrusen pattern and development of late age‐related macular degeneration in the fellow eye of the unilateral case. Jpn J Ophthalmol 2019; 63: 374–381. 31267312 10.1007/s10384-019-00680-9

[33] Curcio CA . Soft drusen in age‐related macular degeneration: Biology and targeting via the oil spill strategies. Invest Ophthalmol Vis Sci 2018; 59: AMD160‐AMD181. 30357336 10.1167/iovs.18-24882 PMC6733535

[34] Sarks S , Cherepanoff S , Killingsworth M & Sarks J . Relationship of basal laminar deposit and membranous debris to the clinical presentation of early age‐related macular degeneration. Invest Ophthalmol Vis Sci 2007; 48: 968–977. 17325134 10.1167/iovs.06-0443

[35] Armstrong J , Hubbard LD , Davis MD , et al. Association of calcified drusen with progression of AMD in AREDS participants. Invest Ophthalmol Vis Sci 2006; 47: 2128 (ARVO abstract).

[36] Tan ACS , Pilgrim MG , Fearn S , et al. Calcified nodules in retinal drusen are associated with disease progression in age‐related macular degeneration. Sci Transl Med 2018; 10: pii:eaat4544. 10.1126/scitranslmed.aat4544 PMC10721335 30404862

[37] Zweifel SA , Spaide RF , Curcio CA , Malek G & Imamura Y . Reticular pseudodrusen are subretinal drusenoid deposits. Ophthalmology 2010; 117: 303–312. 19815280 10.1016/j.ophtha.2009.07.014

[38] Pumariega NM , Smith RT , Sohrab MA , LeTien V & Souied EH . A prospective study of reticular macular disease. Ophthalmology 2011; 118: 1619–1625. 21550118 10.1016/j.ophtha.2011.01.029 PMC3150615

[39] Kaszubski PA , Ben Ami T , Saade C , et al. Changes in reticular pseudodrusen area in eyes that progressed from early to late age‐related macular degeneration. Int Ophthalmol 2018; 38: 503–511. 28265823 10.1007/s10792-017-0485-7 PMC5589480

[40] Joachim N , Mitchell P , Rochtchina E , Tan AG & Wang JJ . Incidence and progression of reticular drusen in age‐related macular degeneration: findings from an older Australian cohort. Ophthalmology 2014; 121: 917–925. 24332537 10.1016/j.ophtha.2013.10.043

[41] Zhou Q , Daniel E , Maguire MG , et al. Pseudodrusen and incidence of late age‐related macular degeneration in fellow eyes in the comparison of age‐related macular degeneration treatments trials. Ophthalmology 2016; 123: 1530–1540. 27040149 10.1016/j.ophtha.2016.02.043 PMC5797653

[42] Marsiglia M , Boddu S , Bearelly S , et al. Association between geographic atrophy progression and reticular pseudodrusen in eyes with dry age‐related macular degeneration. Invest Ophthalmol Vis Sci 2013; 54: 7362–7369. 24114542 10.1167/iovs.12-11073 PMC3823546

[43] Xu LN , Blonska AM , Pumariega NM , et al. Reticular macular disease is associated with multilobular geographic atrophy in age‐related macular degeneration. Retina 2013; 33: 1850–1862. 23632954 10.1097/IAE.0b013e31828991b2 PMC3784629

[44] Boon CJE , Klevering BJ , Hoyng CB , et al. Basal laminar drusen caused by compound heterozygous variants in the CFH gene. Am J Hum Genet 2008; 82: 516–523. 18252232 10.1016/j.ajhg.2007.11.007 PMC2427272

[45] Balaratnasingam C , Cherepanoff S , Dolz‐Marco R , et al. Cuticular drusen clinical phenotypes and natural history defined using multimodal imaging. Ophthalmology 2018; 125: 100–118. 28964580 10.1016/j.ophtha.2017.08.033

[46] Sakurada Y , Parikh R , Gal‐Or O , et al. Cuticular drusen: Risk of geographic atrophy and macular neovascularization. Retina 2018; 40: 257–265. [Epub ahead of print] 10.1097/IAE.0000000000002399 31972795

[47] Cukras C , Agron E , Klein ML , et al. Natural history of drusenoid pigment epithelial detachment in age‐related macular degeneration: Age‐Related Eye Disease Study Report. No. 28. Ophthalmology 2010; 117: 489–499. 20079925 10.1016/j.ophtha.2009.12.002 PMC2947750

[48] Ho J , Witkin AJ , Liu J , et al. Documentation of intraretinal retinal pigment epithelium migration via high‐speed ultrahigh‐resolution optical coherence tomography. Ophthalmology 2011; 118: 687–693. 21093923 10.1016/j.ophtha.2010.08.010 PMC3070873

[49] Mitsuhiro MRKH , Eguchi S & Yamashita H . Regulation mechanisms of retinal pigment epithelial cell migration by the TGF‐beta superfamily. Acta Ophthalmol Scand 2003; 81: 630–638. 14641267 10.1111/j.1395-3907.2003.00170.x

[50] Ferrara D , Silver RE , Louzada RN , Novais EA , Collins GK & Seddon JM . Optical coherence tomography features preceding the onset of advanced age‐related macular degeneration. Invest Ophthalmol Vis Sci 2017; 58: 3519–3529. 28715590 10.1167/iovs.17-21696 PMC5512971

[51] Nassisi M , Fan W , Shi Y , et al. Quantity of intraretinal hyperreflective foci in patients with intermediate age‐related macular degeneration correlates with 1‐year progression. Invest Ophthalmol Vis Sci 2018; 59: 3431–3439. 30025092 10.1167/iovs.18-24143

[52] Christenbury JG , Folgar FA , O'Connell RV , et al. Progression of intermediate age‐related macular degeneration with proliferation and inner retinal migration of hyperreflective foci. Ophthalmology 2013; 120: 1038–1045. 23352193 10.1016/j.ophtha.2012.10.018 PMC3640702

[53] Fragiotta S , Rossi T , Cutini A , Grenga PL & Vingolo EM . Predictive factors for development of neovascular age-related macular degeneration: A spectral‐domain optical coherence tomography study. Retina 2018; 38: 245–252. 28166160 10.1097/IAE.0000000000001540

[54] Gass JD , Agarwal A , Lavina AM & Tawansy KA . Focal inner retinal hemorrhages in patients with drusen: an early sign of occult choroidal neovascularization and chorioretinal anastomosis. Retina 2003; 23: 741–751. 14707822 10.1097/00006982-200312000-00001

[55] Klein ML , Ferris FL , Gensler G , et al. Clinical features and natural course of drusenoid pigment epithelial detachments in age‐related macular degeneration. Invest Ophthalmol Vis Sci 2004; 45: U57 (ARVO abstract).

[56] Roquet W , Roudot‐Thoraval F , Coscas G & Soubrane G . Clinical features of drusenoid pigment epithelial detachment in age related macular degeneration. Br J Ophthalmol 2004; 88: 638–642. 15090415 10.1136/bjo.2003.017632 PMC1772148

[57] Biarnes M , Arias L , Alonso J , et al. Increased fundus autofluorescence and progression of geographic atrophy secondary to age‐related macular degeneration: The GAIN Study. Am J Ophthalmol 2015; 160: 345–353. 25982972 10.1016/j.ajo.2015.05.009

[58] Lindblad AS , Lloyd PC , Clemons TE , et al. Change in area of geographic atrophy in the Age‐Related Eye Disease Study: AREDS report number 26. Arch Ophthalmol 2009; 127: 1168–1174. 19752426 10.1001/archophthalmol.2009.198 PMC6500457

[59] Schmitz‐Valckenberg S , Sahel JA , Danis R , et al. Natural history of geographic atrophy progression secondary to age‐related macular degeneration (Geographic Atrophy Progression Study). Ophthalmology 2016; 123: 361–368. 26545317 10.1016/j.ophtha.2015.09.036

[60] Sunness JS , Margalit E , Srikumaran D , et al. The long‐term natural history of geographic atrophy from age‐related macular degeneration: enlargement of atrophy and implications for interventional clinical trials. Ophthalmology 2007; 114: 271–277. 17270676 10.1016/j.ophtha.2006.09.016 PMC2562326

[61] Yehoshua Z , Rosenfeld PJ , Gregori G , et al. Progression of geographic atrophy in age‐related macular degeneration imaged with spectral domain optical coherence tomography. Ophthalmology 2011; 118: 679–686. 21035861 10.1016/j.ophtha.2010.08.018 PMC3070862

[62] Domalpally A , Danis RP , White J , et al. Circularity index as a risk factor for progression of geographic atrophy. Ophthalmology 2013; 120: 2666–2671. 24206616 10.1016/j.ophtha.2013.07.047

[63] Keenan TD , Agron E , Domalpally A , et al. Progression of geographic atrophy in age‐related macular degeneration: AREDS2 Report Number 16. Ophthalmology 2018; 125: 1913–1928. 30060980 10.1016/j.ophtha.2018.05.028 PMC6246813

[64] Feuer WJ , Yehoshua Z , Gregori G , et al. Square root transformation of geographic atrophy area measurements to eliminate dependence of growth rates on baseline lesion measurements: a reanalysis of age‐related eye disease study report no. 26. JAMA Ophthalmol 2013; 131: 110–111. 23307222 10.1001/jamaophthalmol.2013.572 PMC11551521

[65] Batioglu F , Gedik Oguz Y , Demirel S & Ozmert E . Geographic atrophy progression in eyes with age‐related macular degeneration: role of fundus autofluorescence patterns, fellow eye and baseline atrophy area. Ophthalmic Res 2014; 52: 53–59. 24993093 10.1159/000361077

[66] Holz FG , Bindewald‐Wittich A , Fleckenstein M , et al. Progression of geographic atrophy and impact of fundus autofluorescence patterns in age‐related macular degeneration. Am J Ophthalmol 2007; 143: 463–472. 17239336 10.1016/j.ajo.2006.11.041

[67] Jeong YJ , Hong IH , Chung JK , Kim KL , Kim HK & Park SP . Predictors for the progression of geographic atrophy in patients with age‐related macular degeneration: fundus autofluorescence study with modified fundus camera. Eye (Lond) 2014; 28: 209–218. 24458203 10.1038/eye.2013.275 PMC3930277

[68] Farazdaghi MK & Ebrahimi KB . Role of the choroid in age‐related macular degeneration: A current review. J Ophthalmic Vis Res 2019; 14: 78–87. 30820291 10.4103/jovr.jovr_125_18 PMC6388521

[69] Seddon JM , McLeod DS , Bhutto IA , et al. Histopathological insights into choroidal vascular loss in clinically documented cases of age‐related macular degeneration. JAMA Ophthalmol 2016; 134: 1272–1280. 27657855 10.1001/jamaophthalmol.2016.3519 PMC6014730

[70] Nassisi M , Baghdasaryan E , Borrelli E , Ip M & Sadda SR . Choriocapillaris flow impairment surrounding geographic atrophy correlates with disease progression. PLoS One 2019; 14: e0212563. 30794627 10.1371/journal.pone.0212563 PMC6386298

[71] Querques G , Srour M , Massamba N , et al. Functional characterization and multimodal imaging of treatment‐naive "quiescent" choroidal neovascularization. Invest Ophthalmol Vis Sci 2013; 54: 6886–6892. 24084095 10.1167/iovs.13-11665

[72] Bailey ST , Thaware O , Wang J , et al. Detection of nonexudative choroidal neovascularization and progression to exudative choroidal neovascularization using OCT angiography. Ophthalmol Retina 2019; 3: 629–636. 31068262 10.1016/j.oret.2019.03.008 PMC6684834

[73] Dias JRD , Zhang Q , Garcia JMB , et al. Natural history of subclinical neovascularization in nonexudative age‐related macular degeneration using swept‐source OCT angiography. Ophthalmology 2018; 125: 255–266. 28964581 10.1016/j.ophtha.2017.08.030 PMC11402511

[74] Serra R , Coscas F , Boulet JF , et al. Predictive activation biomarkers of treatment‐naive asymptomatic choroidal neovascularization in age‐related macular degeneration. Retina 2019; 1–10. 10.1097/IAE.0000000000002604. 31259809

[75] Lee JY , Folgar FA , Maguire MG , et al. Outer retinal tubulation in the comparison of age‐related macular degeneration treatments trials (CATT). Ophthalmology 2014; 121: 2423–2431. 25064723 10.1016/j.ophtha.2014.06.013 PMC4254295

[76] Hariri A , Nittala MG & Sadda SR . Outer retinal tubulation as a predictor of the enlargement amount of geographic atrophy in age‐related macular degeneration. Ophthalmology 2015; 122: 407–413. 25315664 10.1016/j.ophtha.2014.08.035

[77] Clemons TE , Milton RC , Klein R , Seddon JM , Ferris FL 3rd & Age‐Related Eye Disease Study Research G . Risk factors for the incidence of Advanced Age‐Related Macular Degeneration in the Age‐Related Eye Disease Study (AREDS) AREDS report no. 19. Ophthalmology 2005; 112: 533–539. 15808240 10.1016/j.ophtha.2004.10.047 PMC1513667

[78] Farinha CVL , Cachulo ML , Alves D , et al. Incidence of age‐related macular degeneration in the central region of Portugal: The Coimbra Eye Study ‐ Report 5. Ophthalmic Res 2019; 61: 226–235. 30820012 10.1159/000496393

[79] Grunwald JE , Pistilli M , Daniel E , et al. Incidence and growth of geographic atrophy during 5 Years of comparison of age‐related macular degeneration treatments trials. Ophthalmology 2017; 124: 97–104. 28079023 10.1016/j.ophtha.2016.09.012 PMC5234734

[80] Hoffman JD , van Grinsven MJ , Li C , et al. Genetic association analysis of drusen progression. Invest Ophthalmol Vis Sci 2016; 57: 2225–2231. 27116550 10.1167/iovs.15-18571 PMC4849854

[81] Joachim ND , Mitchell P , Kifley A & Wang JJ . Incidence, progression, and associated risk factors of medium drusen in age‐related macular degeneration: Findings from the 15‐year follow‐up of an Australian Cohort. JAMA Ophthalmol 2015; 133: 698–705. 25838066 10.1001/jamaophthalmol.2015.0498

[82] Klein R , Knudtson MD , Lee KE , Gangnon RE & Klein BE . Age‐period‐cohort effect on the incidence of age‐related macular degeneration: the Beaver Dam Eye Study. Ophthalmology 2008; 115: 1460–1467. 18762073 10.1016/j.ophtha.2008.01.026 PMC2577776

[83] Klein R , Lee KE , Tsai MY , Cruickshanks KJ , Gangnon RE & Klein BEK . Oxidized low‐density lipoprotein and the incidence of age‐related macular degeneration. Ophthalmology 2019; 126: 752–758. 30572074 10.1016/j.ophtha.2018.12.026 PMC6475598

[84] Lechanteur YT , van de Ven JP , Smailhodzic D , et al. Genetic, behavioral, and sociodemographic risk factors for second eye progression in age‐related macular degeneration. Invest Ophthalmol Vis Sci 2012; 53: 5846–5852. 22815349 10.1167/iovs.11-7731

[85] McGuinness MB , Karahalios A , Simpson JA , et al. Past physical activity and age‐related macular degeneration: the Melbourne Collaborative Cohort Study. Br J Ophthalmol 2016; 100: 1353–1358. 26787681 10.1136/bjophthalmol-2015-307663

[86] Merle BM , Silver RE , Rosner B & Seddon JM . Dietary folate, B vitamins, genetic susceptibility and progression to advanced nonexudative age‐related macular degeneration with geographic atrophy: a prospective cohort study. Am J Clin Nutr 2016; 103: 1135–1144. 26961928 10.3945/ajcn.115.117606 PMC4807698

[87] Merle BMJ , Silver RE , Rosner B & Seddon JM . Associations between vitamin D intake and progression to incident advanced age‐related macular degeneration. Invest Ophthalmol Vis Sci 2017; 58: 4569–4578. 28892825 10.1167/iovs.17-21673 PMC5595226

[88] Ngai LY , Stocks N , Sparrow JM , et al. The prevalence and analysis of risk factors for age‐related macular degeneration: 18‐year follow‐up data from the Speedwell eye study, United Kingdom. Eye (Lond) 2011; 25: 784–793. 21436849 10.1038/eye.2011.56 PMC3178119

[89] Reynolds R , Rosner B & Seddon JM . Dietary omega‐3 fatty acids, other fat intake, genetic susceptibility, and progression to incident geographic atrophy. Ophthalmology 2013; 120: 1020–1028. 23481534 10.1016/j.ophtha.2012.10.020 PMC3758110

[90] Sardell RJ , Persad PJ , Pan SS , et al. Progression rate from intermediate to advanced age‐related macular degeneration is correlated with the number of risk alleles at the CFH locus. Invest Ophthalmol Vis Sci 2016; 57: 6107–6115. 27832277 10.1167/iovs.16-19519 PMC5104418

[91] Seddon JM , Silver RE , Kwong M & Rosner B . Risk prediction for progression of macular degeneration: 10 common and rare genetic variants, demographic, environmental, and macular covariates. Invest Ophthalmol Vis Sci 2015; 56: 2192–2202. 25655794 10.1167/iovs.14-15841 PMC4405097

[92] Yu Y , Reynolds R , Rosner B , Daly MJ & Seddon JM . Prospective assessment of genetic effects on progression to different stages of age‐related macular degeneration using multistate Markov models. Invest Ophthalmol Vis Sci 2012; 53: 1548–1556. 22247473 10.1167/iovs.11-8657 PMC3339916

[93] Yip JL , Khawaja AP , Chan MP , et al. Cross sectional and longitudinal associations between cardiovascular risk factors and age related macular degeneration in the EPIC‐Norfolk Eye Study. PLoS One 2015; 10: e0132565. 26176222 10.1371/journal.pone.0132565 PMC4503731

[94] Jonasson F , Fisher DE , Eiriksdottir G , et al. Five‐year incidence, progression, and risk factors for age‐related macular degeneration: the age, gene/environment susceptibility study. Ophthalmology 2014; 121: 1766–1772. 24768241 10.1016/j.ophtha.2014.03.013 PMC4145014

[95] Wang IK , Lin HJ , Wan L , Lin CL , Yen TH & Sung FC . Risk of age‐related macular degeneration in end‐stage renal disease patients receiving long‐term dialysis. Retina 2016; 36: 1866–1873. 26966867 10.1097/IAE.0000000000001011

[96] Colijn JM , Buitendijk GHS , Prokofyeva E , et al. Prevalence of age‐related macular degeneration in Europe: The past and the future. Ophthalmology 2017; 124: 1753–1763. 28712657 10.1016/j.ophtha.2017.05.035 PMC5755466

[97] Ehrlich R , Harris A , Kheradiya NS , Winston DM , Ciulla TA & Wirostko B . Age‐related macular degeneration and the aging eye. Clin Interv Aging 2008; 3: 473–482. 18982917 10.2147/cia.s2777 PMC2682379

[98] Klein R , Knudtson MD , Cruickshanks KJ & Klein BE . Further observations on the association between smoking and the long‐term incidence and progression of age‐related macular degeneration: the Beaver Dam Eye Study. Arch Ophthalmol 2008; 126: 115–121. 18195228 10.1001/archopht.126.1.115

[99] GBD 2017 Causes of Death Collaborators . Global, regional, and national age‐sex‐specific mortality for 282 causes of death in 195 countries and territories, 1980‐2017: a systematic analysis for the Global Burden of Disease Study 2017. Lancet 2018; 392: 1736–1788. 30496103 10.1016/S0140-6736(18)32203-7 PMC6227606

[100] Fraser‐Bell S , Wu J , Klein R , Azen SP & Varma R . Smoking, alcohol intake, estrogen use, and age‐related macular degeneration in latinos: The Los Angeles Latino Eye Study. Am J Ophthalmol 2006; 141: 79–87. 16386980 10.1016/j.ajo.2005.08.024

[101] Vingerling JR , Dielemans I , Witteman JC , Hofman A , Grobbee DE & de Jong PT . Macular degeneration and early menopause: a case‐control study. BMJ 1995; 310: 1570–1571. 7787646 10.1136/bmj.310.6994.1570 PMC2549930

[102] Klein BE , Klein R & Lee KE . Reproductive exposures, incident age‐related cataracts, and age‐related maculopathy in women: The Beaver Dam Eye Study. Am J Ophthalmol 2000; 130: 322–326. 11020411 10.1016/s0002-9394(00)00474-8

[103] Defay R , Pinchinat S , Lumbroso S , Sutan C , Delcourt C & POLA Study Group . Sex steroids and age‐related macular degeneration in older French women: The POLA study. Ann Epidemiol 2004; 14: 202–208. 15036224 10.1016/S1047-2797(03)00130-3

[104] Freeman EE , Munoz B , Bressler SB & West SK . Hormone replacement therapy, reproductive factors, and age‐related macular degeneration: The Salisbury Eye Evaluation Project. Ophthalmic Epidemiol 2005; 12: 37–45. 15848919 10.1080/09286580490907779

[105] Nirmalan PK , Katz J , Robin AL , et al. Female reproductive factors and eye disease in a rural south Indian population: The Aravind Comprehensive Eye Survey. Invest Ophthalmol Vis Sci 2004; 45: 4273–4276. 15557432 10.1167/iovs.04-0285

[106] Vladan B , Biljana SP , Mandusic V , Zorana M & Zivkovic L . Instability in X chromosome inactivation patterns in AMD: a new risk factor? Med Hypothesis Discov Innov Ophthalmol 2013; 2: 74–82. 24600647 PMC3939760

[107] Wang JJ , Rochtchina E , Smith W , et al. Combined effects of complement factor H genotypes, fish consumption, and inflammatory markers on long‐term risk for age‐related macular degeneration in a cohort. Am J Epidemiol 2009; 169: 633–641. 19074778 10.1093/aje/kwn358 PMC2732972

[108] Saunier V , Merle BMJ , Delyfer MN , et al. Incidence of and risk factors associated with age‐related macular degeneration: Four‐year follow‐up from the ALIENOR Study. JAMA Ophthalmol 2018; 136: 473–481. 29596588 10.1001/jamaophthalmol.2018.0504 PMC5876848

[109] Cho E , Hankinson SE , Rosner B , Willett WC & Colditz GA . Prospective study of lutein/zeaxanthin intake and risk of age‐related macular degeneration. Am J Clin Nutr 2008; 87: 1837–1843. 18541575 10.1093/ajcn/87.6.1837 PMC2504741

[110] Seddon JM , Cote J , Davis N & Rosner B . Progression of age‐related macular degeneration: association with body mass index, waist circumference, and waist‐hip ratio. Arch Ophthalmol 2003; 121: 785–792. 12796248 10.1001/archopht.121.6.785

[111] Buch H , Vinding T , la Cour M , Jensen GB , Prause JU & Nielsen NV . Risk factors for age‐related maculopathy in a 14‐year follow‐up study: the Copenhagen City Eye Study. Acta Ophthalmol Scand 2005; 83: 409–418. 16029262 10.1111/j.1600-0420.2005.00492.x

[112] Vingerling JR , Hofman A , Grobbee DE & de Jong PT . Age‐related macular degeneration and smoking. The Rotterdam Study. Arch Ophthalmol 1996; 114: 1193–1196. 8859077 10.1001/archopht.1996.01100140393005

[113] Adams MK , Simpson JA , Aung KZ , et al. Abdominal obesity and age‐related macular degeneration. Am J Epidemiol 2011; 173: 1246–1255. 21422060 10.1093/aje/kwr005

[114] Klein R , Klein BEK , & Jensen SC . The relation of cardiovascular disease and its risk factors to the 5-year incidence of age related maculopathy - The Beaver Dam Eye Study. Ophthalmology 1997; 104: 1804–1812. 9373110 10.1016/s0161-6420(97)30023-2

[115] Tan JS , Mitchell P , Smith W & Wang JJ . Cardiovascular risk factors and the long‐term incidence of age‐related macular degeneration: The Blue Mountains Eye Study. Ophthalmology 2007; 114: 1143–1150. 17275090 10.1016/j.ophtha.2006.09.033

[116] Klein R , Klein BE , Tomany SC & Cruickshanks KJ . The association of cardiovascular disease with the long‐term incidence of age‐related maculopathy: The Beaver Dam Eye Study. Ophthalmology 2003; 110: 1273–1280. 12799274 10.1016/S0161-6420(03)00599-2

[117] Peeters A , Magliano DJ , Stevens J , Duncan BB , Klein R & Wong TY . Changes in abdominal obesity and age‐related macular degeneration: the Atherosclerosis Risk in Communities Study. Arch Ophthalmol 2008; 126: 1554–1560. 19001224 10.1001/archopht.126.11.1554 PMC5774859

[118] Johnson EJ , Neuringer M , Russell RM , Schalch W & Snodderly DM . Nutritional manipulation of primate retinas, III: Effects of lutein or zeaxanthin supplementation on adipose tissue and retina of xanthophyll‐free monkeys. Invest Ophthalmol Vis Sci 2005; 46: 692–702. 15671301 10.1167/iovs.02-1192

[119] Merle BM , Silver RE , Rosner B & Seddon JM . Adherence to a Mediterranean diet, genetic susceptibility, and progression to advanced macular degeneration: a prospective cohort study. Am J Clin Nutr 2015; 102: 1196–1206. 26490493 10.3945/ajcn.115.111047 PMC4625588

[120] Merle BMJ , Colijn JM , Cougnard‐Gregoire A , et al. Mediterranean diet and incidence of advanced age‐related macular degeneration: The EYE‐RISK Consortium. Ophthalmology 2019; 126: 381–390. 30114418 10.1016/j.ophtha.2018.08.006

[121] Chiu CJ , Milton RC , Klein R , Gensler G & Taylor A . Dietary carbohydrate and the progression of age‐related macular degeneration: a prospective study from the Age‐Related Eye Disease Study. Am J Clin Nutr 2007; 86: 1210–1218. 17921404 10.1093/ajcn/86.4.1210

[122] Kaushik S , Wang JJ , Flood V , et al. Dietary glycemic index and the risk of age‐related macular degeneration. Am J Clin Nutr 2008; 88: 1104–1110. 18842800 10.1093/ajcn/88.4.1104

[123] Cho E , Hung S , Willett WC , et al. Prospective study of dietary fat and the risk of age‐related macular degeneration. Am J Clin Nutr 2001; 73: 209–218. 11157315 10.1093/ajcn/73.2.209

[124] Chua B , Flood V , Rochtchina E , Wang JJ , Smith W & Mitchell P . Dietary fatty acids and the 5‐year incidence of age‐related maculopathy. Arch Ophthalmol 2006; 124: 981–986. 16832021 10.1001/archopht.124.7.981

[125] Seddon JM , Cote J & Rosner B . Progression of age‐related macular degeneration: Association with dietary fat, transunsaturated fat, nuts, and fish intake. Arch Ophthalmol 2003; 121: 1728–1737. 14662593 10.1001/archopht.121.12.1728 PMC8443211

[126] Tan JS , Wang JJ , Flood V & Mitchell P . Dietary fatty acids and the 10‐year incidence of age‐related macular degeneration: The Blue Mountains Eye Study. Arch Ophthalmol 2009; 127: 656–665. 19433717 10.1001/archophthalmol.2009.76

[127] Wu J , Cho E , Giovannucci EL , et al. Dietary intakes of eicosapentaenoic acid and docosahexaenoic acid and risk of age‐related macular degeneration. Ophthalmology 2017; 124: 634–643. 28153441 10.1016/j.ophtha.2016.12.033 PMC5401792

[128] Knudtson MD , Klein R & Klein BE . Physical activity and the 15‐year cumulative incidence of age‐related macular degeneration: The Beaver Dam Eye Study. Br J Ophthalmol 2006; 90: 1461–1463. 17077116 10.1136/bjo.2006.103796 PMC1857544

[129] Gopinath B , Liew G , Burlutsky G & Mitchell P . Physical activity and the 15‐year incidence of age‐related macular degeneration. Invest Ophthalmol Vis Sci 2014; 55: 7799–7803. 25389200 10.1167/iovs.14-15575

[130] Tomany SC , Cruickshanks KJ , Klein R , Klein BE & Knudtson MD . Sunlight and the 10‐year incidence of age‐related maculopathy: The Beaver Dam Eye Study. Arch Ophthalmol 2004; 122: 750–757. 15136324 10.1001/archopht.122.5.750

[131] Klein BE , Howard KP , Iyengar SK , et al. Sunlight exposure, pigmentation, and incident age‐related macular degeneration. Invest Ophthalmol Vis Sci 2014; 55: 5855–5861. 25125603 10.1167/iovs.14-14602 PMC4165367

[132] Klein R , Klein BE , Knudtson MD , et al. Prevalence of age‐related macular degeneration in 4 racial/ethnic groups in the multi‐ethnic study of atherosclerosis. Ophthalmology 2006; 113: 373–380. 16513455 10.1016/j.ophtha.2005.12.013

[133] Chang MA , Bressler SB , Munoz B & West SK . Racial differences and other risk factors for incidence and progression of age‐related macular degeneration: Salisbury Eye Evaluation (SEE) Project. Invest Ophthalmol Vis Sci 2008; 49: 2395–2402. 18263809 10.1167/iovs.07-1584

[134] Age‐Related Eye Disease Study Research Group . Risk factors associated with age‐related macular degeneration. A case‐control study in the age‐related eye disease study: Age‐Related Eye Disease Study Report Number 3. Ophthalmology 2000; 107: 2224–2232. 11097601 10.1016/s0161-6420(00)00409-7 PMC1470467

[135] Ciardella AP , Donsoff IM , Huang SJ , Costa DL & Yannuzzi LA . Polypoidal choroidal vasculopathy. Surv Ophthalmol 2004; 49: 25–37. 14711438 10.1016/j.survophthal.2003.10.007

[136] Pang CP , Baum L , Chan WM , Lau TC , Poon PM & Lam DS . The apolipoprotein E epsilon4 allele is unlikely to be a major risk factor of age‐related macular degeneration in Chinese. Ophthalmologica 2000; 214: 289–291. 10859513 10.1159/000027506

[137] Cugati S , Mitchell P , Rochtchina E , Tan AG , Smith W & Wang JJ . Cataract surgery and the 10‐year incidence of age‐related maculopathy: The Blue Mountains Eye Study. Ophthalmology 2006; 113: 2020–2025. 16935334 10.1016/j.ophtha.2006.05.047

[138] Krishnaiah S , Das T , Nirmalan PK , et al. Risk factors for age‐related macular degeneration: findings from the Andhra Pradesh Eye Disease Study in South India. Invest Ophthalmol Vis Sci 2005; 46: 4442–4449. 16303932 10.1167/iovs.05-0853

[139] Ho L , Boekhoorn SS , Liana, , et al. Cataract surgery and the risk of aging macula disorder: The Rotterdam Study. Invest Ophthalmol Vis Sci 2008; 49: 4795–4800. 18599571 10.1167/iovs.08-2066

[140] Baatz H , Darawsha R , Ackermann H , et al. Phacoemulsification does not induce neovascular age‐related macular degeneration. Invest Ophthalmol Vis Sci 2008; 49: 1079–1083. 18326733 10.1167/iovs.07-0557

[141] Chew EY , Sperduto RD , Milton RC , et al. Risk of advanced age‐related macular degeneration after cataract surgery in the Age‐Related Eye Disease Study: AREDS report 25. Ophthalmology 2009; 116: 297–303. 19091420 10.1016/j.ophtha.2008.09.019 PMC3021282

[142] Rosen ES . Age‐related macular degeneration and cataract surgery. J Cataract Refract Surg 2014; 40: 173–174. 24461495 10.1016/j.jcrs.2013.11.022

[143] Casparis H , Lindsley K , Kuo IC , Sikder S & Bressler NM . Surgery for cataracts in people with age‐related macular degeneration. Cochrane Database Syst Rev 2017. 10.1002/14651858.CD006757.pub4 PMC5419431 28206671

[144] Lundstrom M , Brege KG , Floren I , Lundh B , Stenevi U & Thorburn W . Cataract surgery and quality of life in patients with age related macular degeneration. Br J Ophthalmol 2002; 86: 1330–1335. 12446358 10.1136/bjo.86.12.1330 PMC1771417

[145] Choudhury F , Varma R , McKean‐Cowdin R , Klein R & Azen SP . Risk factors for four‐year incidence and progression of age‐related macular degeneration: The Los Angeles Latino Eye Study. Am J Ophthalmol 2011; 152: 385–395. 21679916 10.1016/j.ajo.2011.02.025 PMC3159714

[146] Klein R , Klein BE , Knudtson MD , et al. Subclinical atherosclerotic cardiovascular disease and early age‐related macular degeneration in a multiracial cohort: The Multiethnic Study of Atherosclerosis. Arch Ophthalmol 2007; 125: 534–543. 17420374 10.1001/archopht.125.4.534

[147] Metelitsina TI , Grunwald JE , DuPont JC & Ying GS . Effect of systemic hypertension on foveolar choroidal blood flow in age related macular degeneration. Br J Ophthalmol 2006; 90: 342–346. 16488959 10.1136/bjo.2005.082974 PMC1856936

[148] Chen YJ , Yeung L , Sun CC , Huang CC , Chen KS & Lu YH . Age‐related macular degeneration in chronic kidney disease: A meta‐analysis of observational studies. Am J Nephrol 2018; 48: 278–291. 30336463 10.1159/000493924

[149] Klein R , Knudtson MD , Lee KE & Klein BE . Serum cystatin C level, kidney disease markers, and incidence of age‐related macular degeneration: The Beaver Dam Eye Study. Arch Ophthalmol 2009; 127: 193–199. 19204238 10.1001/archophthalmol.2008.551 PMC2737458

[150] Liew G , Mitchell P , Wong TY , Iyengar SK & Wang JJ . CKD increases the risk of age‐related macular degeneration. J Am Soc Nephrol 2008; 19: 806–811. 18216312 10.1681/ASN.2007080844 PMC2390960

[151] Gopinath B , Liew G , Kifley A & Mitchell P . Thyroid dysfunction and ten‐year incidence of age‐related macular degeneration. Invest Ophthalmol Vis Sci 2016; 57: 5273–5277. 27716857 10.1167/iovs.16-19735

[152] Lin SY , Hsu WH , Lin CL , et al. Evidence for an association between macular degeneration and thyroid cancer in the aged population. Int J Environ Res Public Health 2018; 15: 902. 10.3390/ijerph15050902 PMC5981941 29751509

[153] Chaker L , Buitendijk GH , Dehghan A , et al. Thyroid function and age‐related macular degeneration: a prospective population‐based cohort study–The Rotterdam Study. BMC Med 2015; 13: 94. 25903050 10.1186/s12916-015-0329-0 PMC4407352

[154] Tsai CC , Kao SC , Cheng CY , et al. Oxidative stress change by systemic corticosteroid treatment among patients having active graves ophthalmopathy. Arch Ophthalmol 2007; 125: 1652–1656. 18071117 10.1001/archopht.125.12.1652

[155] Ma HW , Thapa A , Morris L , Redmond TM , Baehr W & Ding XQ . Suppressing thyroid hormone signaling preserves cone photoreceptors in mouse models of retinal degeneration. Proc Natl Acad Sci USA 2014; 111: 3602–3607. 24550448 10.1073/pnas.1317041111 PMC3948228

[156] Duncan KG , Bailey KR , Baxter JD & Schwartz DM . The human fetal retinal pigment epithelium: A target tissue for thyroid hormones. Ophthalmic Res 1999; 31: 399–406. 10474068 10.1159/000055564

[157] Zhang W , Liu H , Al‐Shabrawey M , Caldwell RW & Caldwell RB . Inflammation and diabetic retinal microvascular complications. J Cardiovasc Dis Res 2011; 2: 96–103. 21814413 10.4103/0975-3583.83035 PMC3144626

[158] Biscetti L , Luchetti E , Vergaro A , Menduno P , Cagini C & Parnetti L . Associations of Alzheimer's disease with macular degeneration. Front Biosci (Elite Ed) 2017; 9: 174–191. 27814598 10.2741/e794

[159] Ratnayaka JA , Serpell LC & Lotery AJ . Dementia of the eye: the role of amyloid beta in retinal degeneration. Eye (Lond) 2015; 29: 1013–1026. 26088679 10.1038/eye.2015.100 PMC4541342

[160] Toops KA , Tan LX & Lakkaraju A . Apolipoprotein E Isoforms and AMD. Adv Exp Med Biol 2016; 854: 3–9. 26427386 10.1007/978-3-319-17121-0_1

[161] Bojanowski CM , Shen D , Chew EY , et al. An apolipoprotein E variant may protect against age‐related macular degeneration through cytokine regulation. Environ Mol Mutagen 2006; 47: 594–602. 16823865 10.1002/em.20233 PMC1899525

[162] Keenan TD , Goldacre R & Goldacre MJ . Associations between age‐related macular degeneration, Alzheimer disease, and dementia: record linkage study of hospital admissions. JAMA Ophthalmol 2014; 132: 63–68. 24232933 10.1001/jamaophthalmol.2013.5696

[163] Rong SS , Lee BY , Kuk AK , et al. Comorbidity of dementia and age‐related macular degeneration calls for clinical awareness: a meta‐analysis. Br J Ophthalmol 2019; 103: 1777–1783. 31000510 10.1136/bjophthalmol-2018-313277

[164] Brilliant MH , Vaziri K , Connor TB , et al. Mining retrospective data for virtual prospective drug repurposing: L‐DOPA and age‐related macular degeneration. Am J Med 2016; 129: 292–298. 26524704 10.1016/j.amjmed.2015.10.015 PMC4841631

[165] Locke CJ , Congrove NR , Stamer WD , Bowen TJ , Stamer WD & McKay BS . Controlled exosome release from the retinal pigment epithelium in situ. Exp Eye Res 2014; 129: 1–4. 25311167 10.1016/j.exer.2014.10.010

[166] Fritsche LG , Igl W , Bailey JN , et al. A large genome‐wide association study of age‐related macular degeneration highlights contributions of rare and common variants. Nat Genet 2016; 48: 134–143. 26691988 10.1038/ng.3448 PMC4745342

[167] Yan Q , Ding Y , Liu Y , et al. Genome‐wide analysis of disease progression in age‐related macular degeneration. Hum Mol Genet 2018; 27: 929–940. 29346644 10.1093/hmg/ddy002 PMC6059197

[168] Dietzel M , Pauleikhoff D , Arning A , et al. The contribution of genetic factors to phenotype and progression of drusen in early age‐related macular degeneration. Graefes Arch Clin Exp Ophthalmol 2014; 252: 1273–1281. 24970616 10.1007/s00417-014-2690-7

[169] Farwick A , Wellmann J , Stoll M , Pauleikhoff D & Hense HW . Susceptibility genes and progression in age‐related maculopathy: a study of single eyes. Invest Ophthalmol Vis Sci 2010; 51: 731–736. 19797206 10.1167/iovs.09-3953

[170] Seddon JM , Reynolds R , Yu Y & Rosner B . Three new genetic loci (R1210C in CFH, variants in COL8A1 and RAD51B) are independently related to progression to advanced macular degeneration. PLoS One 2014; 9: e87047. 24498017 10.1371/journal.pone.0087047 PMC3909074

[171] Seddon JM , Silver RE & Rosner B . Response to AREDS supplements according to genetic factors: survival analysis approach using the eye as the unit of analysis. Br J Ophthalmol 2016; 100: 1731–1737. 27471039 10.1136/bjophthalmol-2016-308624 PMC6570490

[172] Grassmann F , Fleckenstein M , Chew EY , et al. Clinical and genetic factors associated with progression of geographic atrophy lesions in age‐related macular degeneration. PLoS One 2015; 10: e0126636. 25962167 10.1371/journal.pone.0126636 PMC4427438

[173] Miyake M , Yamashiro K , Tamura H , et al. The contribution of genetic architecture to the 10‐year incidence of age‐related macular degeneration in the fellow eye. Invest Ophthalmol Vis Sci 2015; 56: 5353–5361. 26275133 10.1167/iovs.14-16020

[174] Kanda A , Chen W , Othman M , et al. A variant of mitochondrial protein LOC387715/ARMS2, not HTRA1, is strongly associated with age‐related macular degeneration. Proc Natl Acad Sci USA 2007; 104: 16227–16232. 17884985 10.1073/pnas.0703933104 PMC1987388

[175] Kortvely E , Hauck SM , Behler J , Ho N & Ueffing M . The unconventional secretion of ARMS2. Hum Mol Genet 2016; 25: 3143–3151. 27270414 10.1093/hmg/ddw162

[176] Yang Z , Camp NJ , Sun H , et al. A variant of the HTRA1 gene increases susceptibility to age‐related macular degeneration. Science 2006; 314: 992–993. 17053109 10.1126/science.1133811

[177] Canfield AE , Hadfield KD , Rock CF , Wylie EC & Wilkinson FL . HtrA1: a novel regulator of physiological and pathological matrix mineralization? Biochem Soc Trans 2007; 35: 669–671. 17635117 10.1042/BST0350669

[178] van Leeuwen EM , Emri E , Merle BMJ , et al. A new perspective on lipid research in age‐related macular degeneration. Prog Retin Eye Res 2018; 67: 56–86. 29729972 10.1016/j.preteyeres.2018.04.006

[179] Klein R , Myers CE , Buitendijk GH , et al. Lipids, lipid genes, and incident age‐related macular degeneration: the three continent age‐related macular degeneration consortium. Am J Ophthalmol 2014; 158(513–524): e513. 10.1016/j.ajo.2014.05.027 PMC4138281 24879949

[180] Geerlings MJ , de Jong EK & den Hollander AI . The complement system in age‐related macular degeneration: A review of rare genetic variants and implications for personalized treatment. Mol Immunol 2017; 84: 65–76. 27939104 10.1016/j.molimm.2016.11.016 PMC5380947

[181] Taylor RL , Poulter JA , Downes SM , et al. Loss‐of‐function mutations in the CFH gene affecting alternatively encoded factor H‐like 1 protein cause dominant early‐onset macular drusen. Ophthalmology 2019; 126: 1410–1421. 30905644 10.1016/j.ophtha.2019.03.013 PMC6856713

[182] Kersten E , Geerlings MJ , den Hollander AI , et al. Phenotype characteristics of patients with age‐related macular degeneration carrying a rare variant in the complement factor H gene. JAMA Ophthalmol 2017; 135: 1037–1044. 28859202 10.1001/jamaophthalmol.2017.3195 PMC5710490

[183] Kersten E , Paun CC , Schellevis RL , et al. Systemic and ocular fluid compounds as potential biomarkers in age‐related macular degeneration. Surv Ophthalmol 2018; 63: 9–39. 28522341 10.1016/j.survophthal.2017.05.003

[184] Hou HY , Liang HL , Wang YS , et al. A therapeutic strategy for choroidal neovascularization based on recruitment of mesenchymal stem cells to the sites of lesions. Mol Ther 2010; 18: 1837–1845. 20647999 10.1038/mt.2010.144 PMC2951561

[185] Bai Y , Liang S , Yu W , et al. Semaphorin 3A blocks the formation of pathologic choroidal neovascularization induced by transforming growth factor beta. Mol Vis 2014; 20: 1258–1270. 25352735 PMC4168834

[186] Cai J , Nelson KC , Wu M , Sternberg P Jr & Jones DP . Oxidative damage and protection of the RPE. Prog Retin Eye Res 2000; 19: 205–221. 10674708 10.1016/s1350-9462(99)00009-9

[187] Wu EW , Schaumberg DA , Park SK . Environmental cadmium and lead exposures and age‐related macular degeneration in U.S. adults: The National Health and Nutrition Examination Survey 2005 to 2008. Environ Res 2014; 133: 178–184. 24959985 10.1016/j.envres.2014.05.023 PMC4124906

[188] Tokarz P , Kaarniranta K & Blasiak J . Role of antioxidant enzymes and small molecular weight antioxidants in the pathogenesis of age‐related macular degeneration (AMD). Biogerontology 2013; 14: 461–482. 24057278 10.1007/s10522-013-9463-2 PMC3824279

[189] West S , Vitale S , Hallfrisch J , et al. Are antioxidants or supplements protective for age‐related macular degeneration? Arch Ophthalmol 1994; 112: 222–227. 8311777 10.1001/archopht.1994.01090140098031

[190] Moeller SM , Parekh N , Tinker L , et al. Associations between intermediate age‐related macular degeneration and lutein and zeaxanthin in the Carotenoids in Age‐related Eye Disease Study (CAREDS): Ancillary study of the Women's Health Initiative. Arch Ophthalmol 2006; 124: 1151–1162. 16908818 10.1001/archopht.124.8.1151

[191] Li ZY , Tso MO , Wang HM & Organisciak DT . Amelioration of photic injury in rat retina by ascorbic acid: a histopathologic study. Invest Ophthalmol Vis Sci 1985; 26: 1589–1598. 4055291

[192] van Leeuwen R , Boekhoorn S , Vingerling JR , et al. Dietary intake of antioxidants and risk of age‐related macular degeneration. JAMA 2005; 294: 3101–3107. 16380590 10.1001/jama.294.24.3101

[193] Christen WG , Ajani UA , Glynn RJ , et al. Prospective cohort study of antioxidant vitamin supplement use and the risk of age‐related maculopathy. Am J Epidemiol 1999; 149: 476–484. 10067908 10.1093/oxfordjournals.aje.a009836

[194] VandenLangenberg GM , Mares‐Perlman JA , Klein R , Klein BEK , Brady WE & Palta M . Associations between antioxidant and zinc intake and the 5‐year incidence of early age‐related maculopathy in The Beaver Dam Eye Study. Am J Epidemiol 1998; 148: 204–214. 9676703 10.1093/oxfordjournals.aje.a009625

[195] Flood V , Smith W , Wang JJ , Manzi F , Webb K & Mitchell P . Dietary antioxidant intake and incidence of early age‐related maculopathy: The Blue Mountains Eye Study. Ophthalmology 2002; 109: 2272–2278. 12466170 10.1016/s0161-6420(02)01263-0

[196] Tan JS , Wang JJ , Flood V , Rochtchina E , Smith W & Mitchell P . Dietary antioxidants and the long‐term incidence of age‐related macular degeneration: The Blue Mountains Eye Study. Ophthalmology 2008; 115: 334–341. 17664009 10.1016/j.ophtha.2007.03.083

[197] Coral K , Raman R , Rathi S , et al. Plasma homocysteine and total thiol content in patients with exudative age‐related macular degeneration. Eye (Lond) 2006; 20: 203–207. 15803172 10.1038/sj.eye.6701853

[198] Age‐Related Eye Disease Study Research Group . A randomized, placebo‐controlled, clinical trial of high‐dose supplementation with vitamins C and E, beta carotene, and zinc for age‐related macular degeneration and vision loss: AREDS report no. 8. Arch Ophthalmol 2001; 119: 1417–1436. 11594942 10.1001/archopht.119.10.1417 PMC1462955

[199] Albanes D , Heinonen OP , Taylor PR , et al. Alpha‐Tocopherol and beta‐carotene supplements and lung cancer incidence in the alpha‐tocopherol, beta‐carotene cancer prevention study: effects of base‐line characteristics and study compliance. J Natl Cancer Inst 1996; 88: 1560–1570. 8901854 10.1093/jnci/88.21.1560

[200] Omenn GS , Goodman GE , Thornquist MD , et al. Risk factors for lung cancer and for intervention effects in CARET, the Beta‐Carotene and Retinol Efficacy Trial. J Natl Cancer Inst 1996; 88: 1550–1559. 8901853 10.1093/jnci/88.21.1550

[201] Wu J , Cho E , Willett WC , Sastry SM & Schaumberg DA . Intakes of lutein, zeaxanthin, and other carotenoids and age‐related macular degeneration during 2 decades of prospective follow‐up. JAMA Ophthalmol 2015; 133: 1415–1424. 26447482 10.1001/jamaophthalmol.2015.3590 PMC5119484

[202] Ho L , van Leeuwen R , Witteman JC , et al. Reducing the genetic risk of age‐related macular degeneration with dietary antioxidants, zinc, and omega‐3 fatty acids: The Rotterdam Study. Arch Ophthalmol 2011; 129: 758–766. 21670343 10.1001/archophthalmol.2011.141

[203] Chiu CJ , Klein R , Milton RC , Gensler G & Taylor A . Does eating particular diets alter the risk of age‐related macular degeneration in users of the Age‐Related Eye Disease Study supplements? Br J Ophthalmol 2009; 93: 1241–1246. 19508997 10.1136/bjo.2008.143412 PMC3033729

[204] Krinsky NI , Landrum JT & Bone RA . Biologic mechanisms of the protective role of lutein and zeaxanthin in the eye. Annu Rev Nutr 2003; 23: 171–201. 12626691 10.1146/annurev.nutr.23.011702.073307

[205] Chew EY , Clemons TE , Sangiovanni JP , et al. Secondary analyses of the effects of lutein/zeaxanthin on age‐related macular degeneration progression: AREDS2 report No. 3. JAMA Ophthalmol 2014; 132: 142–149. 24310343 10.1001/jamaophthalmol.2013.7376 PMC4636082

[206] Chew EY , Clemons TE , SanGiovanni JP , et al. Lutein plus zeaxanthin and omega‐3 fatty acids for age‐related macular degeneration The Age‐Related Eye Disease Study 2 (AREDS2) randomized clinical trial. JAMA 2013; 309: 2005–2015. 23644932 10.1001/jama.2013.4997

[207] Korobelnik JF , Rougier MB , Delyfer MN , et al. Effect of dietary supplementation with lutein, zeaxanthin, and omega‐3 on macular pigment: A randomized clinical trial. JAMA Ophthalmol 2017; 135: 1259–1266. 28973076 10.1001/jamaophthalmol.2017.3398 PMC5710391

[208] Ma L , Liu R , Du JH , Liu T , Wu SS & Liu XH . Lutein, zeaxanthin and meso‐zeaxanthin supplementation associated with macular pigment optical density. Nutrients 2016; 8: pii: E426. 27420092 10.3390/nu8070426 PMC4963902

[209] Conrady CD , Bell JP , Besch BM , et al. Correlations between macular, skin, and serum carotenoids. Invest Ophthalmol Vis Sci 2017; 58: 3616–3627. 28728169 10.1167/iovs.17-21818 PMC5520678

[210] Robman L , Vu H , Hodge A , et al. Dietary lutein, zeaxanthin, and fats and the progression of age‐related macular degeneration. Can J Ophthalmol 2007; 42: 720–726. 17724493 10.3129/i07-116

[211] Smailhodzic D , van Asten F , Blom AM , et al. Zinc supplementation inhibits complement activation in age‐related macular degeneration. PLoS One 2014; 9: e112682. 25393287 10.1371/journal.pone.0112682 PMC4231060

[212] Gonzalez‐Iglesias H , Alvarez L , Garcia M , Petrash C , Sanz‐Medel A & Coca‐Prados M . Metallothioneins (MTs) in the human eye: A perspective article on the zinc‐MT redox cycle. Metallomics 2014; 6: 201–208. 24419560 10.1039/c3mt00298e

[213] Ugarte M & Osborne NN . Recent advances in the understanding of the role of zinc in ocular tissues. Metallomics 2014; 6: 189–200. 24253309 10.1039/c3mt00291h

[214] King JC , Brown KH , Gibson RS , et al. Biomarkers of Nutrition for Development (BOND)‐Zinc Review. J Nutr 2016; 146: 858S–885S. 10.3945/jn.115.220079 PMC4807640 26962190

[215] Liszewski MK , Java A , Schramm EC & Atkinson JP . Complement dysregulation and disease: insights from contemporary genetics. Annu Rev Pathol 2017; 12: 25–52. 27959629 10.1146/annurev-pathol-012615-044145 PMC6020056

[216] Sohn JH , Kaplan HJ , Suk HJ , Bora PS & Bora NS . Chronic low level complement activation within the eye is controlled by intraocular complement regulatory proteins. Invest Ophthalmol Vis Sci 2000; 41: 3492–3502. 11006244 PMC1851917

[217] Toomey CB , Johnson LV & Bowes Rickman C . Complement factor H in AMD: Bridging genetic associations and pathobiology. Prog Retin Eye Res 2018; 62: 38–57. 28928087 10.1016/j.preteyeres.2017.09.001 PMC5776047

[218] Klein R , Myers CE , Cruickshanks KJ , et al. Markers of inflammation, oxidative stress, and endothelial dysfunction and the 20‐year cumulative incidence of early age‐related macular degeneration: The Beaver Dam Eye Study. JAMA Ophthalmol 2014; 132: 446–455. 24481424 10.1001/jamaophthalmol.2013.7671 PMC4076038

[219] Seddon JM , George S , Rosner B & Rifai N . Progression of age‐related macular degeneration: prospective assessment of C‐reactive protein, interleukin 6, and other cardiovascular biomarkers. Arch Ophthalmol 2005; 123: 774–782. 15955978 10.1001/archopht.123.6.774

[220] Krogh Nielsen M , Subhi Y , Molbech CR , Falk MK , Nissen MH & Sorensen TL . Systemic levels of interleukin‐6 correlate with progression rate of geographic atrophy secondary to age‐related macular degeneration. Invest Ophthalmol Vis Sci 2019; 60: 202–208. 30644965 10.1167/iovs.18-25878

[221] Merle BMJ , Delyfer MN , Korobelnik JF , et al. High concentrations of plasma n3 fatty acids are associated with decreased risk for late age‐related macular degeneration. J Nutr 2013; 143: 505–511. 23406618 10.3945/jn.112.171033

[222] Merle BM , Benlian P , Puche N , et al. Circulating omega‐3 Fatty acids and neovascular age‐related macular degeneration. Invest Ophthalmol Vis Sci 2014; 55: 2010–2019. 24557349 10.1167/iovs.14-13916

[223] Souied E , Delcourt C , Querques G , et al. Nat2 Study: Omega‐3 levels in red blood cells membranes correlates the preventive effect. Invest Ophthalmol Vis Sci 2013; 54: 3277 (ARVO abstract).

[224] Wu J , Cho E , Giovannucci EL , et al. Dietary intake of alpha‐linolenic acid and risk of age‐related macular degeneration. Am J Clin Nutr 2017; 105: 1483–1492. 28468892 10.3945/ajcn.116.143453 PMC5445670

[225] van Leeuwen R , Klaver CC , Vingerling JR , et al. Cholesterol and age‐related macular degeneration: is there a link? Am J Ophthalmol 2004; 137: 750–752. 15059717 10.1016/j.ajo.2003.09.015

[226] Chong EW , Robman LD , Simpson JA , et al. Fat consumption and its association with age‐related macular degeneration. Arch Ophthalmol 2009; 127: 674–680. 19433719 10.1001/archophthalmol.2009.60

[227] Parekh N , Voland RP , Moeller SM , et al. Association between dietary fat intake and age‐related macular degeneration in the Carotenoids in Age‐Related Eye Disease Study (CAREDS): An ancillary study of the Women's Health Initiative. Arch Ophthalmol 2009; 127: 1483–1493. 19901214 10.1001/archophthalmol.2009.130 PMC3144752

[228] SanGiovanni JP , Chew EY , Agron E , et al. The relationship of dietary omega‐3 long‐chain polyunsaturated fatty acid intake with incident age‐related macular degeneration: AREDS report no. 23. Arch Ophthalmol 2008; 126: 1274–1279. 18779490 10.1001/archopht.126.9.1274 PMC2812063

[229] Ding Y , Liu Y , Yan Q , et al. Bivariate analysis of age‐related macular degeneration progression using genetic risk scores. Genetics 2017; 206: 119–133. 28341650 10.1534/genetics.116.196998 PMC5419464

[230] Klein ML , Francis PJ , Ferris FL 3rd , Hamon SC & Clemons TE . Risk assessment model for development of advanced age‐related macular degeneration. Arch Ophthalmol 2011; 129: 1543–1550. 21825180 10.1001/archophthalmol.2011.216

[231] Perlee LT , Bansal AT , Gehrs K , et al. Inclusion of genotype with fundus phenotype improves accuracy of predicting choroidal neovascularization and geographic atrophy. Ophthalmology 2013; 120: 1880–1892. 23523162 10.1016/j.ophtha.2013.02.007 PMC3695024

[232] Buitendijk G, Rochtchina E , Myers C , et al. Increasing the fidelity of AMD prediction models using population data from three continents. Invest Ophthalmol Vis Sci 2013; 54: 224 (ARVO abstract).

[233] Ly A , Nivison‐Smith L , Zangerl B , Assaad N & Kalloniatis M . Advanced imaging for the diagnosis of age‐related macular degeneration: a case vignettes study. Clin Exp Optom 2018; 101: 243–254. 28994139 10.1111/cxo.12607 PMC5873408

[234] de Oliveira Dias JR , Zhang Q , Garcia JMB , et al. Natural history of subclinical neovascularization in nonexudative age‐related macular degeneration using swept‐source OCT angiography. Ophthalmology 2018; 125: 255–266. 28964581 10.1016/j.ophtha.2017.08.030 PMC11402511

[235] Torkamani A , Wineinger NE & Topol EJ . The personal and clinical utility of polygenic risk scores. Nat Rev Genet 2018; 19: 581–590. 29789686 10.1038/s41576-018-0018-x

[236] den Hollander AI & de Jong EK . Highly penetrant alleles in age‐related macular degeneration. Cold Spring Harb Perspect Med 2014; 5: a017202. 25377141 10.1101/cshperspect.a017202 PMC4355254

[237] Brown CN , Green BD , Thompson RB , den Hollander AI & Lengyel I . Metabolomics and age‐related macular degeneration. Metabolites 2018; 9: pii: E4. 30591665 10.3390/metabo9010004 PMC6358913

[238] Hampton BM , Kovach JL & Schwartz SG . Pharmacogenetics and nutritional supplementation in age‐related macular degeneration. Clin Ophthalmol 2015; 9: 873–876. 26028959 10.2147/OPTH.S84155 PMC4440436

[239] Buitendijk GHS , Rochtchina E , Myers C , et al. Prediction of age‐related macular degeneration in the general population: The Three Continent AMD Consortium. Ophthalmology 2013; 120: 2644–2655. 24120328 10.1016/j.ophtha.2013.07.053 PMC3986722

