# Supplementary material for: Risk factors for progression of age‐related macular degeneration
Source: Ophthalmic Physiol Opt. 2020 Feb 25;40(2):140–70. doi: 10.1111/opo.12675 (PMC7155063; doi:10.1111/opo.12675)
Supplement: Supplementary file 1 — Table S1. Overview of the association between phenotypic factors and the prediction to late AMD in prospective cohort studies Table S2. Overview of the association between demographic and environmental factors and the prediction to early and late AMD in prospective cohort studies Table S3. Overview of the top hits in the 34 AMD associated loci, as identified in the case‐control GWAS (Fritsche et al. 2016) from the International AMD Genomics Consortium, and their association with disease progression as described in the GWAS of the AREDS study (Yan et al. 2018), and several prospective cohort studies Table S4. Overview of the association between anti‐oxidative factors and the prediction of early and late AMD in prospective cohort studies Table S5. Overview of the association between immune factors and the prediction of early and late AMD in prospective cohort studies Table S6. Overview of the association between lipid factors and the prediction of early and late AMD in prospective cohort studies [file OPO-40-140-s001.docx]

**Risk Factors for Progression of Age-related Macular Degeneration**

Thomas J. Heesterbeek, Laura Lorés-Motta, Carel B. Hoyng, Yara T.E. Lechanteur,
Anneke I. den Hollander

**Supplementary Tables**

**Supplementary Table 1.** Overview of the association between phenotypic factors and the prediction to late AMD in prospective cohort studies

|  |  |  | **Prediction to late AMD** | | | | | | | | | | | | | | | | | | | |
| --- | --- | --- | --- | --- | --- | --- | --- | --- | --- | --- | --- | --- | --- | --- | --- | --- | --- | --- | --- | --- | --- | --- |
| **Reference** | **Follow-up (years)** | **Imaging modalities** | **Drusen area/volume** | **Drusen location** | **Medium and large drusen** | **Calcified drusen** | **Reticular pseudo drusen** | **Cuticular drusen** | **Pigmentary changes** | **Hyperreflective foci** | **Pigment epithelial detachment** | **GA: Lesion size in mm2** | **GA: lesion size in mm** | **GA: lesion number** | **GA: lesion location** | **GA: lesion shape** | **GA: perilesional FAF pattern** | **Choroidal abnormalities** | **Choriocapillaris flow impairments** | **Quiescent CNV/Subclinical CNV** | **Incomplete RPE and outer retina atrophy** | **Outer retinal tubulations** |
| Joachim (2013)^23^ | 15 | CFP | ↑ | ↑ | ↑ | - | ↑ | - | ↑ | - | - | - | - | - | - | - | - | - | - | - | - | - |
| Shim (2016)^21^ | 4.4 | CFP | ↑ | ↑ | - | - | - | - | NA | - | - | - | - | - | - | - | - | - | - | - | - | - |
| Lei (2017)^17^ | 1.2 | OCT | ↑ | - | - | - | - | - | - | ↑ | - | - | - | - | - | - | - | - | - | - | - | - |
| Yehoshua (2011)^61^ | 1.2 | OCT | ↑ | - | - | - | - | - | - | - | - | ↑† | NA† | ↑† | - | - | - | - | - | - | - | - |
| Abdelfattah (2016)^26^ | 2 | OCT | ↑ | - | - | - | - | - | - | - | - | - | - | - | - | - | - | - | - | - | - | - |
| Folgar (2016)^25^ | 2 | CFP/OCT | ↑ | - | - | - | - | - | - | - | - | - | - | - | - | - | - | - | - | - | - | - |
| Heesterbeek (2019)^24^ | 6.5 | CFP | ↑ | - | - | - | - | - | - | - | - | - | - | - | - | - | - | - | - | - | - | - |
| Nathoo (2014)^22^ | 2.8 | OCT | ↑ | - | - | - | - | - | - | - | - | - | - | - | - | - | - | - | - | - | - | - |
| Schlanitz (2017)^16^ | 3 | OCT | ↑ | - | - | - | - | - | - | - | - | - | - | - | - | - | - | - | - | - | - | - |
| Hallak (2019)^27^ | 2 | OCT | - | ↑ | - | - | - | - | - | - | - | - | - | - | - | - | - | - | - | - | - | - |
| Brader (2013)^18^ | 5 | CFP/FAG | - | - | ↑ | ↑ | - | - | - | - | ↑ | - | - | - | - | - | - | - | - | - | - | - |
| Finger (2014)^30^ | 2.3 | NIR/OCT | - | - | ↑ | - | ↑ | - | ↑ | - | - | - | - | - | - | - | - | - | - | - | - | - |
| Sakurada (2019)^32^ | 33 | CFP/OCT | - | - | ↑ | - | ↑ | - | - | - | - | - | - | - | - | - | - | NA | - | - | - | - |
| Tikellis (2007)^20^ | 7.2 | CFP | - | - | ↑ | - | - | - | NA | - | - | - | - | - | - | - | - | - | - | - | - | - |
| Connolly (2018)^31^ | 4 | CFP | - | - | ↑ | - | - | - | ↑ | - | - | - | - | - | - | - | - | - | - | - | - | - |
| Ferris (2013)^29^ | 5 | CFP | - | - | ↑ | - | - | - | ↑ | - | - | - | - | - | - | - | - | - | - | - | - | - |
| Armstrong (2006)^35^ | 5 | CFP | - | - | - | ↑ | - | - | - | - | - | - | - | - | - | - | - | - | - | - | - | - |
| Tan (2018)^36^ | 1 | OCT | - | - | - | ↑ | - | - | - | - | - | - | - | - | - | - | - | - | - | - | - | - |
| Marsiglia (2013)^42^ | 0.9 | FAF/NIR | - | - | - | - | ↑† | - | - | - | - | - | - | ↑† | - | - | - | - | - | - | - | - |
| Xu (2013)^43^ | 2.3 | FAF/NIR | - | - | - | - | ↑ | - | - | - | - | - | - | ↑† | - | - | - | - | - | - | - | - |
| Joachim (2014)^40^ | 15 | CFP | - | - | - | - | ↑ | - | - | - | - | - | - | - | - | - | - | - | - | - | - | - |
| Kaszubski (2018)^39^ | 2 | FAF/NIR | - | - | - | - | ↑ | - | - | - | - | - | - | - | - | - | - | - | - | - | - | - |
| Pumariega (2011)^38^ | 3 | CFP/NIR | - | - | - | - | ↑ | - | - | - | - | - | - | - | - | - | - | - | - | - | - | - |
| Zhou (2016)^41^ | 2 | CFP/NIR | - | - | - | - | ↑ | - | - | - | - | - | - | - | - | - | - | - | - | - | - | - |
| Balaratnasingam (2018)^45^ | 3.7 | OCT/FAF | - | - | - | - | - | ↑ | - | - | - | - | - | - | - | - | - | - | - | - | - | - |
| Sakurada (2018)^46^ | 3.3 | CFP/OCT | - | - | - | - | - | ↑ | - | - | - | - | - | - | - | - | - | - | - | - | - | - |
| Cukras (2010)^47^ | 8 | CFP | - | - | - | - | - | - | ↑ | - | ↑ | - | - | - | - | - | - | - | - | - | - | - |
| Ferrara (2017)^50^ | 9.1 | OCT | - | - | - | - | - | - | - | ↑ | ↑ | - | - | - | - | - | - | ↑ | - | - | ↑ | - |
| Fragiotta (2018)^53^ | 2 | OCT | - | - | - | - | - | - | - | ↑ | ↑ | - | - | - | - | - | - | - | - | - | - | - |
| Ouyang (2013)^19^ | 1.8 | OCT | - | - | - | - | - | - | - | ↑ | - | - | - | - | - | - | - | ↑ | - | - | - | - |
| Christenbury (2013)^52^ | 2 | OCT | - | - | - | - | - | - | - | ↑ | - | - | - | - | - | - | - | - | - | - | - | - |
| Nassisi (2018)^51^ | 1 | OCT | - | - | - | - | - | - | - | ↑ | - | - | - | - | - | - | - | - | - | - | - | - |
| Klein (2004)^55^ | 8.5 | CFP | - | - | - | - | - | - | - | - | ↑ | - | - | - | - | - | - | - | - | - | - | - |
| Roquet (2004)^56^ | 4.6 | CFP/FAF | - | - | - | - | - | - | - | - | ↑ | - | - | - | - | - | - | - | - | - | - | - |
| Domalpally (2013)^62^ | 2 | CFP | - | - | - | - | - | - | - | - | - | ↑† | NA† | ↑† | ↑† | ↑† | - | - | - | - | - | - |
| Schmitz-Valckenberg(2016)^59^ | 1.5 | CFP/FAF | - | - | - | - | - | - | - | - | - | ↑† | - | ↑† | ↑† | - | ↑† | - | - | - | - | - |
| Biarnes (2015)^57^ | 1.5 | FAF | - | - | - | - | - | - | - | - | - | ↑† | - | - | ↑† | - | NA† | - | - | - | - | - |
| Lindblad (2009)^58^ | 4.8 | CFP | - | - | - | - | - | - | - | - | - | ↑† | - | - | - | - | - | - | - | - | - | - |
| Sunness (2007)^60^ | 4.3 | CFP | - | - | - | - | - | - | - | - | - | ↑† | - | - | - | - | - | - | - | - | - | - |
| Feuer (2013)^64^ | 4 | CFP | - | - | - | - | - | - | - | - | - | - | NA† | - | - | - | - | - | - | - | - | - |
| Keenan (2018)^63^ | 4.4 | CFP/FAF | - | - | - | - | - | - | - | - | - | - | ↑† | - | ↑† | - | - | - | - | - | - | - |
| Batioglu (2014)^65^ | 1.5 | FAF | - | - | - | - | - | - | - | - | - | - | - | - | - | - | ↑† | - | - | - | - | - |
| Holz (2007)^66^ | 1.8 | FAF | - | - | - | - | - | - | - | - | - | - | - | - | - | - | ↑† | - | - | - | - | - |
| Jeong (2014)^67^ | 1 | FAF | - | - | - | - | - | - | - | - | - | - | - | - | - | - | ↑† | - | - | - | - | - |
| Nassisi (2019)^70^ | 1.3 | OCTA | - | - | - | - | - | - | - | - | - | - | - | - | - | - | - | - | ↑† | - | - | - |
| Bailey (2019)^72^ | 2 | OCTA | - | - | - | - | - | - | - | - | - | - | - | - | - | - | - | - | - | ↑ | - | - |
| Dias (2018)^73^ | 4.2 | OCTA | - | - | - | - | - | - | - | - | - | - | - | - | - | - | - | - | - | ↑ | - | - |
| Serra (2019)^74^ | 3.3 | OCTA | - | - | - | - | - | - | - | - | - | - | - | - | - | - | - | - | - | ↑ | - | - |
| Wu (2015)^8^ | 1.7 | FAF/OCT | - | - | - | - | - | - | - | - | - | - | - | - | - | - | - | - | - | - | ↑ | - |
| Lee (2014)^75^ | 2 | OCT | - | - | - | - | - | - | - | - | - | - | - | - | - | - | - | - | - | - | - | ↑ |
| Hariri (2015)^76^ | 1.5 | OCT | - | - | - | - | - | - | - | - | - | - | - | - | - | - | - | - | - | - | - | ↓† |
| **Total ↑** |  |  | **9** | **3** | **7** | **3** | **9** | **2** | **5** | **6** | **6** | **6** | **1** | **5** | **4** | **1** | **4** | **2** | **1** | **3** | **2** | **1** |
| **Total ↓** |  |  | **-** | **-** | **-** | **-** | **-** | **-** | **-** | **-** | **-** | **-** | **-** | **-** | **-** | **-** | **-** | **-** | **-** | **-** | **-** | **1** |
| **Total NA** |  |  | **-** | **-** | **-** | **-** | **-** | **-** | **2** | **-** | **-** | **-** | **3** | **-** | **-** | **-** | **1** | **-** | **-** | **-** | **-** | **-** |

†progression of GA growth

Abbreviations: ↑: increased risk factor; ↓: decreased risk factor; AMD: age-related macular degeneration; CFP: color fundus photography; FAF: fundus autofluorescence; FAG; fluorescein angiography; GA: geographic atrophy; NA: no association found; NIR: near infra-red imaging; OCT: optical coherence tomography; OCTA: optical coherence tomography angiography

**Supplementary Table 2**. Overview of the association between demographic and environmental factors and the prediction to early and late AMD in prospective cohort studies

|  |  |  | **Prediction to early AMD** | | | | | | | | | | | | | | | | **Prediction to late AMD** | | | | | | | | | | | | | | | |
| --- | --- | --- | --- | --- | --- | --- | --- | --- | --- | --- | --- | --- | --- | --- | --- | --- | --- | --- | --- | --- | --- | --- | --- | --- | --- | --- | --- | --- | --- | --- | --- | --- | --- | --- |
| **Reference** | **Follow-up (years)** | **Imaging modalities** | **Age** | **Female sex** | **Smoking** | **Body Mass** | **Diet** | **Physical activity** | **Education level** | **Sunlight exposure** | **Ethnicity** | **Cataract** | **Hypertension** | **Chronic Kidney Disease** | **Hyperthyroidism** | **Diabetes** | **Alzheimer’s Disease** | **Parkinson’s Disease** | **Age** | **Female sex** | **Smoking** | **Body Mass** | **Diet** | **Physical activity** | **Education level** | **Sunlight exposure** | **Ethnicity** | **Cataract** | **Hypertension** | **Chronic Kidney Disease** | **Hyperthyroidism** | **Diabetes** | **Alzheimer’s Disease** | **Parkinson’s Disease** |
| Joachim (2014)^40^ | 15 | CFP | ↑ | ↑ | ↑ | - | NA | - | - | - | - | - | NA | - | - | - | - | - | - | - | - | - | - | - | - | - | - | - | - | - | - | - | - | - |
| Yip (2015)^93^ | 17 | CFP | ↑ | NA | NA | NA | - | NA | NA | - | - | - | NA | - | - | - | - | - | ↑ | NA | NA | NA | - | NA | NA | - | - | - | NA | - | - | - | - | - |
| Yu (2012)^92^ | 10.3 | CFP | ↑ | NA | NA | NA | - | - | NA | - | - | - | - | - | - | - | - | - | ↑ | NA | ↑ | ↑ | - | - | NA | - | - | - | - | - | - | - | - | - |
| Wang (2016)^95^ | 10 | CFP | ↑ | NA | - | - | - | - | - | - | - | - | ↑ | ↑ | - | ↑ | - | - | ↑ | NA | - | - | - | - | - | - | - | - | ↑ | ↑ | - | ↑ | - | - |
| Farinha (2019)^78^ | 6.4 | CFP | ↑ | NA | - | - | - | - | - | - | - | - | - | - | - | - | - | - | ↑ | NA | - | - | - | - | - | - | - | - | - | - | - | - | - | - |
| Klein (2019)^83^ | 25 | CFP | ↑ | NA | - | - | - | - | - | - | - | - | - | - | - | - | - | - | ↑ | NA | - | - | - | - | - | - | - | - | - | - | - | - | - | - |
| Reynolds (2013)^89^ | 10 | CFP | ↑ | NA | - | - | - | - | - | - | - | - | - | - | - | - | - | - | ↑ | NA | - | - | - | - | - | - | - | - | - | - | - | - | - | - |
| Connolly (2018)^31^ | 4 | CFP | ↑ | - | ↑ | - | - | - | - | - | - | - | - | - | - | - | - | - | ↑ | - | ↑ | - | - | - | - | - | - | - | - | - | - | - | - | - |
| Ngai (2011)^88^ | 17.9 | CFP | ↑ | - | NA | NA | - | - | - | - | - | - | ↑ | - | - | - | - | - | ↑ | - | NA | NA | - | - | - | - | - | - | ↑ | - | - | - | - | - |
| McGuinness (2016)^85^ | 11.3 | CFP | NA | ↑ | ↑ | - | NA | ↓ | - | - | - | - | - | - | - | - | - | - | ↑ | NA | ↑ | - | NA | NA | - | - | - | - | - | - | - | - | - | - |
| Jonasson (2014)^94^ | 5 | CFP | NA | NA | ↑ | ↑ | - | - | - | - | - | - | NA | - | - | NA | - | - | ↑ | ↑ | ↑ | ↑ | - | - | - | - | - | - | NA | - | - | NA | - | - |
| Klein (2008)^98^ | 10 | CFP | NA | NA | NA | NA | - | - | ↓ | - | - | - | NA | - | - | - | - | - | ↑ | NA | NA | ↑ | - | - | NA | - | - | - | NA | - | - | - | - | - |
| Keenan (2018)^63^ | 4.4 | CFP/FAF | - | - | ↑ | - | - | - | - | - | - | - | - | - | - | - | - | - | - | - | ↑ | - | - | - | - | - | - | - | - | - | - | - | - | - |
| Buch (2005)^111^ | 14.5 | CFP | - | - | NA | NA | - | NA | NA | - | - | - | NA | - | - | NA | - | - | - | - | NA | NA | - | NA | NA | - |  | - | NA | - | - | NA | - | - |
| Saunier (2018)^108^ | 3.8 | CFP/OCT | - | - | NA | NA | - | - | - | - | - | - | NA | - | - | NA | - | - | - | - | NA | NA | - | - | - | - | - | - | NA | - | - | NA | - | - |
| Cho (2008)^109^ | 18 | CFP | - | - | NA | NA | - | - | - | - | - | - | - | - | - | - | - | - | - | - | NA | NA | - | - | - | - | - | - | - | - | - | - | - | - |
| Hoffman (2016)^80^ | 6.5 | CFP | - | - | NA | - | - | - | - | - | - | - | - | - | - | - | - | - | - | - | - | - | - | - | - | - | - | - | - | - | - | - | - | - |
| Peeters (2008)^117^ | 6 | CFP | - | - | - | ↑ | - | - | - | - | - | - | - | - | - | - | - | - | - | - | - | ↑ | - | - | - | - | - | - | - | - | - | - | - | - |
| Tan (2007)^115^ | 10 | CFP | - | - | - | NA | - | - | - | - | - | - | NA | - | - | NA | - | - | - | - | - | NA | - | - | - | - | - | - | NA | - | - | NA | - | - |
| Adams (2011)^113^ | 11.5 | CFP | - | - | - | ↓ | - | - | - | - | - | - | - | - | - | - | - | - | - | - | - | ↑ | - | - | - | - | - | - | - | - | - | - | - | - |
| Chiu (2007)^121^ | 5.4 | CFP | - | - | - | - | ↑ | - | - | - | - | - | - | - | - | - | - | - | - | - | - | - | ↑ | - | - | - | - | - | - | - | - | - | - | - |
| Kaushik (2008)^122^ | 10 | CFP | - | - | - | - | ↑ | - | - | - | - | - | - | - | - | - | - | - | - | - | - | - | - | - | - | - | - | - | - | - | - | - | - | - |
| Cho (2001)^123^ | 12 | CFP | - | - | - | - | ↓ | - | - | - | - | - | - | - | - | - | - | - | - | - | - | - | ↓ | - | - | - | - | - | - | - | - | - | - | - |
| Chua (2006)^124^ | 5.1 | CFP | - | - | - | - | ↓ | - | - | - | - | - | - | - | - | - | - | - | - | - | - | - | ↓ | - | - | - | - | - | - | - | - | - | - | - |
| Tan (2009)^126^ | 10 | CFP | - | - | - | - | ↓ | - | - | - | - | - | - | - | - | - | - | - | - | - | - | - | ↓ | - | - | - | - | - | - | - | - | - | - | - |
| Wu (2017)^127^ | 28 | MR | - | - | - | - | ↓ | - | - | - | - | - | - | - | - | - | - | - | - | - | - | - | ↓ | - | - | - | - | - | - | - | - | - | - | - |
| Klein (2014)^131^ | 20 | CFP | - | - | - | - | - | - | - | NA | - | - | - | - | - | - | - | - | - | - | - | - | - | - | - | NA | - | - | - | - | - | - | - | - |
| Tomany (2004)^130^ | 10 | CFP | - | - | - | - | - | - | - | NA | - | - | - | - | - | - | - | - | - | - | - | - | - | - | - | - | - | - | - | - | - | - | - | - |
| Klein (2006)^132^ | 10 | CFP | - | - | - | - | - | - | - | - | ↑ | - | - | - | - | - | - | - | - | - | - | - | - | - | - | - | ↑ | - | - | - | - | - | - | - |
| Chang (2008)^133^ | 2 | CFP | - | - | - | - | - | - | - | - | ↑ | - | - | - | - | - | - | - | - | - | - | - | - | - | - | - | - | - | - | - | - | - | - | - |
| Krishnaiah (2005)^138^ | 1 | CFP | - | - | - | - | - | - | - | - | - | ↑ | - | - | - | - | - | - | - | - | - | - | - | - | - | - | - | ↑ | - | - | - | - | - | - |
| Chew (2009)^141^ | 9.5 | CFP | - | - | - | - | - | - | - | - | - | NA | - | - | - | - | - | - | - | - | - | - | - | - | - | - | - | NA | - | - | - | - | - | - |
| Choudhury (2011)^145^ | 4.3 | CFP | - | - | - | - | - | - | - | - | - | - | ↑ | - | - | - | - | - | - | - | - | - | - | - | - | - | - | - | ↑ | - | - | - | - | - |
| Klein (2007)^146^ | 2 | CFP | - | - | - | - | - | - | - | - | - | - | NA | - | - | NA | - | - | - | - | - | - | - | - | - | - | - | - | - | - | - | - | - | - |
| Klein (2009)^149^ | 15 | CFP | - | - | - | - | - | - | - | - | - | - | - | ↑ | - | - | - | - | - | - | - | - | - | - | - | - | - | - | - | NA | - | - | - | - |
| Liew (2008)^150^ | 5 | CFP | - | - | - | - | - | - | - | - | - | - | - | ↑ | - | - | - | - | - | - | - | - | - | - | - | - | - | - | - | - | - | - | - | - |
| Gopinath (2016)^151^ | 10 | CFP | - | - | - | - | - | - | - | - | - | - | - | - | ↑ | - | - | - | - | - | - | - | - | - | - | - | - | - | - | - | ↑ | - | - | - |
| Lin (2018)^152^ | 12 | MR | - | - | - | - | - | - | - | - | - | - | - | - | ↑ | - | - | - | - | - | - | - | - | - | - | - | - | - | - | - | ↑ | - | - | - |
| Keenan (2014)^162^ | 1 | MR | - | - | - | - | - | - | - | - | - | - | - | - | - | - | NA | - | - | - | - | - | - | - | - | - | - | - | - | - | - | - | NA | - |

|  |  |  | **Prediction to early AMD** | | | | | | | | | | | | | | | | **Prediction to late AMD** | | | | | | | | | | | | | | | |
| --- | --- | --- | --- | --- | --- | --- | --- | --- | --- | --- | --- | --- | --- | --- | --- | --- | --- | --- | --- | --- | --- | --- | --- | --- | --- | --- | --- | --- | --- | --- | --- | --- | --- | --- |
| **Reference** | **Follow-up (years)** | **Imaging modalities** | **Age** | **Female sex** | **Smoking** | **Body Mass** | **Diet** | **Physical activity** | **Education level** | **Sunlight exposure** | **Ethnicity** | **Cataract** | **Hypertension** | **Chronic Kidney Disease** | **Hyperthyroidism** | **Diabetes** | **Alzheimer’s Disease** | **Parkinson’s Disease** | **Age** | **Female sex** | **Smoking** | **Body Mass** | **Diet** | **Physical activity** | **Education level** | **Sunlight exposure** | **Ethnicity** | **Cataract** | **Hypertension** | **Chronic Kidney Disease** | **Hyperthyroidism** | **Diabetes** | **Alzheimer’s Disease** | **Parkinson’s Disease** |
| Lechanteur (2012)^84^ | - | MR | - | - | - | - | - | - | - | - | - | - | - | - | - | - | - | - | ↑ | ↑ | ↑ | ↑ | - | - | NA | - | - | - | - | - | - | - | - | - |
| Clemons (2005)^77^ | 6.3 | CFP | - | - | - | - | - | - | - | - | - | - | - | - | - | - | - | - | ↑ | ↑ | ↑ | ↑ | - | - | ↓ | NA | ↑ | NA | NA | - | - | ↑ | - | - |
| Merle (2017)^87^ | 9.4 | CFP/OCT | - | - | - | - | - | - | - | - | - | - | - | - | - | - | - | - | ↑ | ↑ | ↑ | NA | - | - | ↓ | - | - | - | - | - | - | - | - | - |
| Seddon (2015)^91^ | 8.8 | CFP | - | - | - | - | - | - | - | - | - | - | - | - | - | - | - | - | ↑ | NA | ↑ | NA | - | - | NA | - | - | - | - | - | - | - | - | - |
| Joachim (2013)^23^ | 15 | CFP | - | - | - | - | - | - | - | - | - | - | - | - | - | - | - | - | ↑ | NA | ↑ | - | ↓ | - | - | - | - | - | - | - | - | - | - | - |
| Sakurada (2019)^32^ | 3 | OCT/FAF | - | - | - | - | - | - | - | - | - | - | - | - | - | - | - | - | ↑ | NA | ↑ | - | - | - | - | - | - | - | - | - | - | - | - | - |
| Tikellis (2007)^20^ | 7.2 | CFP | - | - | - | - | - | - | - | - | - | - | - | - | - | - | - | - | ↑ | NA | ↑ | - | - | - | - | - | - | - | - | - | - | - | - | - |
| Merle (2016)^86^ | 8.7 | CFP | - | - | - | - | - | - | - | - | - | - | - | - | - | - | - | - | ↑ | NA | NA | ↑ | - | - | NA | - | - | - | - | - | - | - | - | - |
| Joachim (2015)^81^ | 15 | CFP | - | - | - | - | - | - | - | - | - | - | - | - | - | - | - | - | ↑ | NA | NA | - | - | - | - | - | - | - | - | - | - | - | - | - |
| Grunwald (2017)^79^ | 5 | OCT/FAF | - | - | - | - | - | - | - | - | - | - | - | - | - | - | - | - | ↑ | - | - | - | - | - | - | - | - | - | - | - | - | - | - | - |
| Sardell (2016)^90^ | 3 | CFP | - | - | - | - | - | - | - | - | - | - | - | - | - | - | - | - | ↑ | - | - | - | - | - | - | - | - | - | - | - | - | - | - | - |
| Hallak (2019)^27^ | 2 | OCT | - | - | - | - | - | - | - | - | - | - | - | - | - | - | - | - | NA | NA | - | - | - | - | - | - | - | - | - | - | - | - | - | - |
| Shim (2016)^21^ | 4.4 | CFP | - | - | - | - | - | - | - | - | - | - | - | - | - | - | - | - | NA | - | ↑ | NA | - | - | - | - | - | - | ↑ | - | - | - | - | - |
| Biarnes (2015)^57^ | 1.5 | FAF | - | - | - | - | - | - | - | - | - | - | - | - | - | - | - | - | - | NA | NA | - | - | - | - | - | - | NA | NA | - | - | - | - | - |
| Tomany (2004)^2^ | 5.5 | CFP | - | - | - | - | - | - | - | - | - | - | - | - | - | - | - | - | - | - | ↑ | NA | - | - | - | - | - | - | NA | - | - | NA | - | - |
| Wang (2009)^107^ | 10 | CFP | - | - | - | - | - | - | - | - | - | - | - | - | - | - | - | - | - | - | ↑ | - | NA | - | - | - | - | - | - | - | - | - | - | - |
| Seddon (2003)^110^ | 4.6 | CFP | - | - | - | - | - | - | - | - | - | - | - | - | - | - | - | - | - | - | NA | ↑ | - | - | - | - | - | - | - | - | - | - | - | - |
| Seddon (2003)^125^ | 4.6 | CFP | - | - | - | - | - | - | - | - | - | - | - | - | - | - | - | - | - | - | - | ↑ | ↓ | NA | - | - | - | - | - | - | - | - | - | - |
| Klein (2003)^116^ | 10.1 | CFP | - | - | - | - | - | - | - | - | - | - | - | - | - | - | - | - | - | - | - | NA | - | ↓ | - | - | - | - | ↑ | - | - | - | - | - |
| Merle (2015)^119^ | 8.7 | CFP | - | - | - | - | - | - | - | - | - | - | - | - | - | - | - | - | - | - | - | - | ↓ | - | - | - | - | - | - | - | - | - | - | - |
| Merle (2019)^120^ | 9.9 | CFP | - | - | - | - | - | - | - | - | - | - | - | - | - | - | - | - | - | - | - | - | ↓ | - | - | - | - | - | - | - | - | - | - | - |
| Gopinath (2014)^129^ | 15 | CFP | - | - | - | - | - | - | - | - | - | - | - | - | - | - | - | - | - | - | - | - | - | NA | - | - | - | - | - | - | - | - | - | - |
| Knudtson (2006)^128^ | 15 | CFP | - | - | - | - | - | - | - | - | - | - | - | - | - | - | - | - | - | - | - | - | - | ↓ | - | - | - | - | - | - | - | - | - | - |
| Cugati (2006)^137^ | 10 | CFP | - | - | - | - | - | - | - | - | - | - | - | - | - | - | - | - | - | - | - | - | - | - | - | - | - | ↑ | - | - | - | - | - | - |
| Ho (2008)^139^ | 5.7 | CFP | - | - | - | - | - | - | - | - | - | - | - | - | - | - | - | - | - | - | - | - | - | - | - | - | - | ↑ | - | - | - | - | - | - |
| Baatz (2008)^140^ | 1 | MR | - | - | - | - | - | - | - | - | - | - | - | - | - | - | - | - | - | - | - | - | - | - | - | - | - | NA | - | - | - | - | - | - |
| Chaker (2015)^153^ | 6.9 | CFP | - | - | - | - | - | - | - | - | - | - | - | - | - | - | - | - | - | - | - | - | - | - | - | - | - | - | - | - | ↑ | - | - | - |
| Brilliant (2016)^164^ | - | MR | - | - | - | - | - | - | - | - | - | - | - | - | - | - | - | - | - | - | - | - | - | - | - | - | - | - | - | - | - | - | - | ↓ |
| **Total ↑** |  |  | **9** | **2** | **5** | **2** | **2** | **-** | **-** | **-** | **2** | **1** | **3** | **3** | **2** | **1** | **-** | **-** | **22** | **4** | **15** | **10** | **1** | **-** | **-** | **-** | **2** | **3** | **5** | **1** | **3** | **2** | **-** | **-** |
| **Total ↓** |  |  | **-** | **-** | **-** | **1** | **4** | **1** | **1** | **-** | **-** | **-** | **-** | **-** | **-** | **-** | **-** | **-** | **-** | **-** | **-** | **-** | **8** | **2** | **2** | **-** | **-** | **-** | **-** | **-** | **-** | **-** | **-** | **1** |
| **Total NA** |  |  | **3** | **8** | **8** | **8** | **5** | **2** | **3** | **2** | **-** | **1** | **8** | **-** | **-** | **5** | **1** | **-** | **2** | **16** | **10** | **11** | **2** | **5** | **7** | **2** | **-** | **4** | **9** | **1** | **-** | **5** | **1** | **-** |

Abbreviations: ↑: increased risk factor; ↓: decreased risk factor; AMD: age-related macular degeneration; CFP: color fundus photography; FAF: fundus autofluorescence; MR: medical records; NA: no association found; OCT: optical coherence tomography; MR: medical records;

**Supplementary Table 3.** Overview of the top hits in the 34 AMD associated loci, as identified in the case-control GWAS (Fritsche et al. 2016) from the International AMD Genomics Consortium, and their association with disease progression as described in the GWAS of the AREDS study (Yan et al. 2018), and several prospective cohort studies

|  |  | **GWAS** | | **Prediction to early AMD¶** | | | | | | **Prediction to nAMD¶** | | | | | | **Prediction to GA¶** | | | | | | | | | **GA growth¶** | |  |  |  |
| --- | --- | --- | --- | --- | --- | --- | --- | --- | --- | --- | --- | --- | --- | --- | --- | --- | --- | --- | --- | --- | --- | --- | --- | --- | --- | --- | --- | --- | --- |
|  |  | Fritche (2016)‡^166^ | Yan (2018)§^167^ | Dietzel (2014)^168^ | Farwick (2010)^169^ | Joachim (2014)^40^ | Klein (2014)^179^ | Wang (2009)^107^ | Yu (2012)^92^ | Hallak (2019)^27^ | Miyake (2015)^173^ | Seddon (2014)^170^ | Seddon (2016)^171^ | Yan (2018)^167^ | Yu (2012)^92^ | Grunwald (2017)^79^ | Joachim (2013)^23^ | Klein (2014)^179^ | Merle (2016)^86^ | Seddon (2014)^170^ | Seddon (2016)^171^ | Wang (2009)^107^ | Yan (2018)^167^ | Yu (2012)^92^ | Grassmann (2015)^172^ | Keenan (2018)^63^ |  |  |  |
| **Follow-up (years)** |  | **-** | 10.3 | 2.6 | 2.6 | 15 | 20 | 10 | 10.3 | 2 | 10 | 8.8 | 6.6 | 10.3 | 10.3 | 5 | 15 | 20 | 8.7 | 8.8 | 6.6 | 10 | 10.3 | 10.3 | 4.5 | 4.4 |  |  |  |
| **Imaging modality** |  | - | CFP | CFP | CFP | CFP | CFP | CFP | CFP | OCT | CFP | CFP | CFP | CFP | CFP | OCT | CFP | CFP | CFP | CFP | CFP | CFP | CFP | CFP | FAF | FAF |  | **Total** |  |
| **Locus name** | **Index variant** |  |  |  |  |  |  |  |  |  |  |  |  |  |  |  |  |  |  |  |  |  |  |  |  |  | **↑** | **↓** | **NA** |
| ***CFH*** | **rs10922109†** | ↓ | ↓ | - | - | - | - | - | - | NA | - | ↓ | - | - | - | - | - | - | - | NA | - | - | - | - | - | NA | **-** | **2** | **3** |
| ***CFH*** | **rs570618†** | ↑ | NA | ↑ | ↑ | ↑ | - | ↑ | ↑ | - | - | ↑ | ↑ | - | ↑ | NA | ↑ | - | ↑ | NA | ↑ | NA | - | ↑ | NA | NA | **12** | **-** | **6** |
| ***COL4A3*** | **rs11884770** | ↓ | NA | - | - | - | - | - | - | NA | - | - | - | - | - | - | - | - | - | - | - | - | - | - | - | - | **-** | **-** | **2** |
| ***ADAMTS9-AS2*** | **rs62247658†** | ↑ | NA | - | - | - | - | - | - | NA | NA | NA | - | - | - | - | - | - | - | NA | - | - | - | - | - | - | **-** | **-** | **5** |
| ***COL8A1*** | **rs140647181** | ↑ | NA | - | - | - | - | - | - | NA | - | - | - | - | - | - | - | - | - | - | - | - | - | - | - | - | **-** | **-** | **2** |
| ***CFI*** | **rs10033900** | ↑ | NA | - | - | - | - | - | NA | NA | - | NA | - | - | NA | - | - | - | - | NA | - | - | - | ↑ | - | NA | **1** | **-** | **7** |
| ***C9*** | **rs62358361** | ↑ | NA | - | - | - | - | - | - | NA | - | - | - | - | - | - | - | - | - | - | - | - | - | - | - | - | **-** | **-** | **2** |
| ***PRLR/SPEF2*** | **rs114092250** | ↓ | NA | - | - | - | - | - | - | NA | - | - | - | - | - | - | - | - | - | - | - | - | - | - | - | - | **-** | **-** | **2** |
| ***C2/CFB/SKIV2L*** | **rs116503776†** | ↓ | ↓ | - | - | - | - | - | - | NA | ↓ | - | - | - | - | - | - | - | - | - | - | - | - | - | - | NA | **-** | **2** | **2** |
| ***VEGFA*** | **rs943080** | ↓ | NA | - | - | - | - | - | - | NA | NA | NA | - | - | - | - | - | - | - | NA | - | - | - | - | - | - | **-** | **-** | **5** |
| ***KMT2E/SRPK2*** | **rs1142** | ↑ | NA | - | - | - | - | - | - | NA | - | - | - | - | - | - | - | - | - | - | - | - | - | - | - | - | **-** | **-** | **2** |
| ***PILRB/PILRA*** | **rs7803454** | ↑ | NA | - | - | - | - | - | - | NA | - | - | - | - | - | - | - | - | - | - | - | - | - | - | - | - | **-** | **-** | **2** |
| ***TNFRSF10A*** | **rs79037040** | ↓ | NA | - | - | - | - | - | - | NA | - | - | - | - | - | - | - | - | - | - | - | - | - | - | - | - | **-** | **-** | **2** |
| ***MIR6130/RORB*** | **rs10781182** | ↑ | NA | - | - | - | - | - | - | NA | - | - | - | - | - | - | - | - | - | - | - | - | - | - | - | - | **-** | **-** | **2** |
| ***TRPM3*** | **rs71507014** | ↑ | NA | - | - | - | - | - | - | NA | - | - | - | - | - | - | - | - | - | - | - | - | - | - | - | - | **-** | **-** | **2** |
| ***TGFBR1*** | **rs1626340†** | ↓ | NA | - | - | - | - | - | - | NA | NA | NA | - | - | - | - | - | - | - | NA | - | - | - | - | - | - | **-** | **-** | **5** |
| ***ABCA1*** | **rs2740488†** | ↓ | NA | ↓ | - | - | NA | - | ↓ | NA | - | NA | - | - | NA | - | - | NA | - | - | - | - | - | NA | - | NA | **-** | **2** | **8** |
| ***ARHGAP21*** | **rs12357257** | ↑ | NA | - | - | - | - | - | - | NA | - | - | - | - | - | - | - | - | - | - | - | - | - | - | - | - | **-** | **-** | **2** |
| ***ARMS2/HTRA1*** | **rs860846†** | ↑ | ↑ | NA | NA | ↑ | - | - | ↑ | NA | ↑ | ↑ | ↑ | - | ↑ | ↑ | ↑ | - | ↑ | ↑ | ↑ | - | - | ↑ | ↑ | ↑ | **15** | **-** | **3** |
| ***RDH5/CD63*** | **rs3138141** | ↑ | NA | - | - | - | - | - | - | NA | - | - | - | - | - | - | - | - | - | - | - | - | - | - | - | - | **-** | **-** | **2** |
| ***ACAD10*** | **rs61941274** | ↑ | NA | - | - | - | - | - | - | ↑ | - | - | - | - | - | - | - | - | - | - | - | - | - | - | - | - | **1** | **-** | **1** |
| ***B3GALTL*** | **rs9564692** | ↓ | NA | - | - | - | - | - | - | NA | - | - | - | - | - | - | - | - | - | - | - | - | - | - | - | - | **-** | **-** | **2** |
| ***RAD51*** | **rs61985136†** | ↓ | NA | - | - | - | - | - | - | NA | - | ↓ | - | - | - | - | - | - | NA | NA | - | - | - | - | - | NA | **-** | **1** | **5** |
| ***LIPC*** | **rs2043085** | ↓ | NA | - | - | - | - | - | - | NA | - | - | - | ↓ | ↓ | - | - | - | - | - | - | - | ↓ | ↓ | - | NA | **-** | **4** | **3** |
| ***CETP*** | **rs5817082†** | ↓ | NA | - | - | - | ↓ | - | - | - | - | - | - | - | - | - | - | - | - | - | - | - | - | - | - | NA | **-** | **1** | **2** |
| ***CTRB2/CTRB1*** | **rs72802342** | ↓ | NA | - | - | - | - | - | - | NA | - | - | - | ↓ | - | - | - | - | - | - | - | - | ↓ | - | - | - | **-** | **2** | **2** |
| ***TMEM97/VTN*** | **rs11080055** | ↓ | NA | - | - | - | - | - | - | NA | - | - | - | - | - | - | - | - | - | - | - | - | - | - | - | - | **-** | **-** | **2** |
| ***NPLOC4/TSPAN10*** | **rs6565597** | ↑ | NA | - | - | - | - | - | - | NA | - | - | - | - | - | - | - | - | - | - | - | - | - | - | - | - | **-** | **-** | **2** |
| ***C3*** | **rs2230199** | ↑ | ↑ | - | NA | - | - | - | ↑ | NA | - | - | - | - | ↑ | NA | - | - | ↑ | NA | - | - | - | ↑ | ↓ | ↓ | **5** | **2** | **4** |
| ***CNN2*** | **rs67538026** | ↓ | NA | - | - | - | - | - | - | NA | - | - | - | - | - | - | - | - | - | - | - | - | - | - | - | - | **-** | **-** | **2** |
| ***APOE*** | **rs429358** | ↓ | NA | - | - | - | - | - | NA | NA | - | - | - | - | NA | - | - | - | - | - | - | - | - | - | NA | NA | **-** | **-** | **6** |
| ***MMP9*** | **rs142450006** | ↓ | NA | - | - | - | - | - | - | NA | - | - | - | ↓ | - | - | - | - | - | - | - | - | NA | - | - | - | **-** | **1** | **3** |
| ***C20orf85*** | **rs201459901** | ↓ | NA | - | - | - | - | - | - | NA | - | - | - | - | - | - | - | - | - | - | - | - | - | - | - | - | **-** | **-** | **2** |
| ***SYN3/TIMP3*** | **rs5754227** | ↓ | NA | - | - | - | - | - | - | NA | - | - | - | - | - | - | - | - | - | - | - | - | - | - | - | - | **-** | **-** | **2** |
| ***SLC16A8*** | **rs8135665** | ↑ | NA | - | - | - | - | - | - | NA | - | NA | - | - | - | - | - | - | - | NA | - | - | - | - | - | - | **-** | **-** | **4** |

† The following variants were also included since they are in high LD (R^2^>0.80) with the AMD-associated variants of the original discovery GWAS of 2016: *ADAMTS9-AS2:* rs6795735 for rs62247658 (R^2^ 0.984), *ARMS2/HTRA1:* rs10490924 for rs860846 (R^2^ 1.0), *C2/CFB/SKIV2L:* rs429608 for rs116503776 (R^2^ 1.0), *CFH*: rs1410996 for rs10922109 (R^2^ 1.0), *CFH*: rs1061170 for rs570618 (R^2^ 1.0), *RAD51B*: rs8017304 for rs61985136 (R^2^ 1.0), *TGFBR1*: rs334353 for rs1626340 (R^2^ 0.855), *CETP*: rs1864163 for rs5817082 (R^2^ 0.975), *ABCA1*: rs1883025 for rs2740488 (R^2^ 0.941)
‡ Original discovery GWAS, based on case-control study from the International AMD Genomics Consortium using 33,976 participants (no follow-up)
§ GWAS on AMD progression, based on a prospective data from the AREDS study using 2,721 participants (10 years follow-up)
¶ Detailed information of the prospective studies included in this review
Abbreviations: ↑: minor allele is a risk factor; ↓: minor allele is a protective factor; AREDS: Age-Related Eye Disease Study; AMD: age-related macular degeneration; CFP: color fundus photography; FAF: fundus autofluorescence; GA: Geographic atrophy; GWAS: genome wide association
study; NA: no association found; nAMD: neovascular age-related macular degeneration; OCT: optical coherence tomography

**Supplementary Table 4**. Overview of the association between anti-oxidative factors and the prediction of early and late AMD in prospective cohort studies

|  |  |  | **Prediction to early AMD** | | | | | | | | | | | | **Prediction to late AMD** | | | | | | | | | | | |
| --- | --- | --- | --- | --- | --- | --- | --- | --- | --- | --- | --- | --- | --- | --- | --- | --- | --- | --- | --- | --- | --- | --- | --- | --- | --- | --- |
| **Reference** | **Follow-up (years)** | **Imaging modalities** | **Vitamin C** | **Vitamin E** | **Vitamin A** | **Vitamin B** | **Vitamin D** | **α-carotene** | **β-carotene** | **β-cryptoxanthin** | **Lycopene** | **Lutein** | **Zeaxanthin** | **Zinc** | **Vitamin C** | **Vitamin E** | **Vitamin A** | **Vitamin B** | **Vitamin D** | **α-carotene** | **β-carotene** | **β-cryptoxanthin** | **Lycopene** | **Lutein** | **Zeaxanthin** | **Zinc** |
| Flood (2002)^195^ | 5 | CFP | ↑ | - | NA | - | - | NA | NA | NA | NA | NA | NA | - | - | - | - | - | - | - | - | - | - | - | - | - |
| Cho (2008)^109^ | 18 | CFP | NA | NA | - | - | - | - | - | - | - | NA | NA | - | NA | NA | - | - | - | - | - | - | - | NA | NA | - |
| Christen (1999)^193^ | 12 | MR | NA | NA | - | - | - | - | - | - | - | - | - | - | NA | NA | - | - | - | - | - | - | - | - | - | - |
| Van Leeuwen (2005)^192^ | 8 | CFP | NA | ↓ | NA | - | - | NA | NA | NA | NA | NA | NA | ↓ | NA | ↓ | NA | - | - | NA | NA | NA | NA | NA | NA | ↓ |
| Van den Langenberg (1998)^194^ | 10 | CFP | NA | ↓ | NA | - | - | ↓ | NA | NA | NA | NA | NA | ↓ | - | - | - | - | - | - | - | - | - | - | - | - |
| West (1994)^189^ | 2 | CFP | NA | ↓ | NA | - | - | - | NA | - | - | - | - | - | NA | ↓ | NA | - | - | - | NA | - | - | - | - | - |
| Tan (2008)^196^ | 10 | CFP | - | NA | NA | - | - | - | NA | - | - | ↓ | ↓ | ↓ | - | ↑ | NA | - | - | - | ↑ | - | - | ↓ | ↓ | ↓ |
| Wu (2015)^201^ | 26 | MR | - | - | - | - | - | NA | NA | NA | NA | NA | NA | - | - | - | - | - | - | ↓ | ↓ | ↓ | NA | ↓ | ↓ | - |
| Chiu (2009)^203^ | 5.4 | CFP | - | - | - | - | - | - | NA | - | - | - | - | - | - | - | - | - | - | - | ↑ | - | - | - | - | - |
| Ho (2011)^202^ | 8.6 | CFP | - | - | - | - | - | - | ↓ | - | - | ↓ | ↓ | ↓ | - | - | - | - | - | - | - | - | - | - | - | - |
| Robman (2007)^210^ | 7 | CFP | - | - | - | - | - | - | - | - | - | ↑ | ↑ | - | - | - | - | - | - | - | - | - | - | ↑ | ↑ | - |
| Joachim (2014)^40^ | 15 | CFP | - | - | - | - | - | - | - | - | - | NA | NA | - | - | - | - | - | - | - | - | - | - | ↓ | ↓ | - |
| Moeller (2006)^190^ | 7 | CFP | - | - | - | - | - | - | - | - | - | NA | NA | - | - | - | - | - | - | - | - | - | - | - | - | - |
| AREDS Research Group (2001)^198^ | 6.3 | CFP | - | - | - | - | - | - | - | - | - | - | - | - | ↓ | ↓ | - | - | - | - | ↓ | - | - | - | - | ↓ |
| AREDS2 Research Group (2013)^206^ | 4.9 | CFP | - | - | - | - | - | - | - | - | - | - | - | - | ↓ | ↓ | - | - | - | - | - | - | - | ↓ | ↓ | ↓ |
| Merle (2016)^86^ | 8.7 | CFP | - | - | - | - | - | - | - | - | - | - | - | - | - | - | - | NA | - | - | - | - | - | - | - | - |
| Merle (2017)^87^ | 9.4 | CFP/OCT | - | - | - | - | - | - | - | - | - | - | - | - | - | - | - | - | ↓ | - | - | - | - | - | - | - |
| **Total ↑** |  |  | **1** | **-** | **-** | **-** | **-** | **-** | **-** | **-** | **-** | **1** | **1** | **-** | **-** | **1** | **-** | **-** | **-** | **-** | **2** | **-** | **-** | **1** | **1** | **-** |
| **Total ↓** |  |  | **-** | **3** | **-** | **-** | **-** | **1** | **1** | **-** | **-** | **2** | **2** | **4** | **2** | **4** | **-** | **-** | **1** | **1** | **1** | **1** | **-** | **3** | **3** | **4** |
| **Total NA** |  |  | **5** | **3** | **5** | **-** | **-** | **3** | **7** | **4** | **4** | **7** | **7** | **-** | **4** | **2** | **3** | **1** | **-** | **1** | **2** | **1** | **2** | **2** | **2** | **-** |

Abbreviations: ↑: increased risk factor; ↓: decreased risk factor; AMD: age-related macular degeneration; CFP: color fundus photography; MR: medical records; NA: no association found; OCT: optical coherence tomography; MR: medical records;

**Supplementary Table 5**. Overview of the association between immune factors and the prediction of early and late AMD in prospective cohort studies

|  |  |  | **Prediction to early AMD** | | | | | | | | | | **Prediction to late AMD** | | | | | | | | | |
| --- | --- | --- | --- | --- | --- | --- | --- | --- | --- | --- | --- | --- | --- | --- | --- | --- | --- | --- | --- | --- | --- | --- |
| **Reference** | **Follow-up (years)** | **Imaging modalities** | **IL-1β** | **IL-6** | **IL-8** | **IL-10** | **TNF-αR2** | **CRP** | **hsCRP** | **ICAM-1** | **VCAM-1** | **White blood cell count** | **IL-1β** | **IL-6** | **IL-8** | **IL-10** | **TNF-αR2** | **CRP** | **hsCRP** | **ICAM-1** | **VCAM-1** | **White blood cell count** |
| Klein (2014)^218^ | 20 | CFP | - | ↑ | - | - | ↑ | - | ↑ | NA | ↑ | NA | - | - | - | - | - | - | - | - | - | - |
| Ngai (2011)^88^ | 17.9 | CFP | - | - | - | - | - | NA | - | - | - | NA | - | - | - | - | - | NA | - | - | - | NA |
| Yip (2015)^93^ | 17 | CFP | - | - | - | - | - | NA | - | - | - | - | - | - | - | - | - | NA | - | - | - | - |
| Joachim (2014)^40^ | 15 | CFP | - | - | - | - | - | - | - | - | - | NA | - | - | - | - | - | - | - | - | - | - |
| Krogh Nielsen (2019)^220^ | 1 | CFP/OCT | - | - | - | - | - | - | - | - | - | - | NA† | ↑† | NA† | NA† | NA† | NA† | - | - | - | - |
| Seddon (2005)^219^ | 4.6 | CFP | - | - | - | - | - | - | - | - | - | - | - | ↑ | - | - | NA | NA | - | NA | NA | - |
| Jonasson (2014)^94^ | 5 | CFP | - | - | - | - | - | - | - | - | - | - | - | - | - | - | - | NA | - | - | - | - |
| Tomany (2004)^2^ | 5.5 | CFP | - | - | - | - | - | - | - | - | - | - | - | - | - | - | - | - | - | - | - | NA |
| **Total ↑** |  |  | **-** | **1** | **-** | **-** | **1** | **-** | **1** | **-** | **1** | **-** | **-** | **2** | **-** | **-** | **-** | **-** | **-** | **-** | **-** | **-** |
| **Total ↓** |  |  | **-** | **-** | **-** | **-** | **-** | **-** | **-** | **-** | **-** | **-** | **-** | **-** | **-** | **-** | **-** | **-** | **-** | **-** | **-** | **-** |
| **Total NA** |  |  | **-** | **-** | **-** | **-** | **-** | **2** | **-** | **1** | **-** | **3** | **1** | **-** | **1** | **1** | **2** | **5** | **-** | **1** | **1** | **2** |

†progression of GA growth
Abbreviations: ↑: increased risk factor; ↓: decreased risk factor; AMD: age-related macular degeneration; CFP: color fundus photography; CRP: C reactive protein; hsCRP: high-sensitivity C reactive protein; ICAM-1: intercellular adhesion molecule 1; IL-6: interleukin-6; IL-10: interleukin-10; IL-1β: interleukin-1β; NA: no association found; OCT: optical coherence tomography; TNF- αR2: tissue necrosis factor α Receptor 2; VCAM-1: vascular cell adhesion molecule 1

**Supplementary Table 6.** Overview of the association between lipid factors and the prediction of early and late AMD in prospective cohort studies

|  |  |  | **Prediction to early AMD** | | | | | | | | | | | | | **Prediction to late AMD** | | | | | | | | | | | | |
| --- | --- | --- | --- | --- | --- | --- | --- | --- | --- | --- | --- | --- | --- | --- | --- | --- | --- | --- | --- | --- | --- | --- | --- | --- | --- | --- | --- | --- |
| **Reference** | **Follow-up (years)** | **Imaging modalities** | **Cholesterol** | **Triglyceride** | **LDL-C** | **HDL-C** | **ApoA1** | **ApoB** | **SFA** | **MUFA** | **ALA** | **EPA** | **DHA** | **LA** | **AA** | **Cholesterol** | **Triglyceride** | **LDL-C** | **HDL-C** | **ApoA1** | **ApoB** | **SFA** | **MUFA** | **ALA** | **EPA** | **DHA** | **LA** | **AA** |
| Yip (2015)^93^ | 17 | CFP | NA | NA | NA | ↑ | - | - | - | - | - | - | - | - | - | NA | NA | NA | ↑ | - | - | - | - | - | - | - | - | - |
| Saunier (2018)^108^ | 3.8 | CFP/OCT | NA | NA | NA | ↑ | - | - | - | - | - | - | - | - | - | NA | NA | NA | NA | - | - | - | - | - | - | - | - | - |
| Klein (2007)^146^ | 2 | CFP | NA | NA | NA | ↑ | - | - | - | - | - | - | - | - | - | - | - | - | - | - | - | - | - | - | - | - | - | - |
| Tan (2007)^115^ | 10 | CFP | NA | NA | NA | NA | - | - | - | - | - | - | - | - | - | NA | NA | NA | ↓ | - | - | - | - | - | - | - | - | - |
| Buch (2005)^111^ | 14.5 | CFP | NA | NA | - | NA | NA | ↓ | - | - | - | - | - | - | - | ↑ | NA | - | NA | ↑ | NA | - | - | - | - | - | - | - |
| Joachim (2014)^40^ | 15 | CFP | NA | NA | - | - | - | - | - | - | - | - | - | - | - | - | - | - | - | - | - | - | - | - | - | - | - | - |
| Ngai (2011)^88^ | 17.9 | CFP | NA | ↓ | NA | NA | - | - | - | - | - | - | - | - | - | NA | ↓ | NA | NA | - | - | - | - | - | - | - | - | - |
| Klein (2014)^179^ | 20 | CFP | NA | - | - | ↑ | - | - | - | - | - | - | - | - | - | NA | - | - | NA | - | - | - | - | - | - | - | - | - |
| Cho (2001)^123^ | 12 | CFP | - | - | - | - | - | - | NA | NA | ↑ | NA | ↓ | ↑ | NA | - | - | - | - | - | - | NA | NA | ↑ | NA | ↓ | ↑ | NA |
| Chong (2009)^226^ | 13 | CFP | - | - | - | - | - | - | NA | NA | NA | NA | NA | NA | NA | - | - | - | - | - | - | NA | NA | NA | NA | NA | NA | NA |
| Chua (2006)^124^ | 5.1 | CFP | - | - | - | - | - | - | NA | NA | NA | - | - | NA | NA | - | - | - | - | - | - | NA | NA | NA | - | - | NA | NA |
| Tan (2009)^126^ | 10 | CFP | - | - | - | - | - | - | NA | NA | NA | - | - | NA | NA | - | - | - | - | - | - | NA | NA | NA | - | - | NA | NA |
| Parekh (2009)^227^ | 7 | CFP | - | - | - | - | - | - | NA | NA | - | - | - | - | - | - | - | - | - | - | - | - | - | - | - | - | - | - |
| Wu (2017)^224^ | 28 | CFP | - | - | - | - | - | - | - | - | ↑ | - | - | - | - | - | - | - | - | - | - | - | - | - | - | - | - | - |
| Chiu (2009)^203^ | 5.4 | CFP | - | - | - | - | - | - | - | - | - | NA | NA | - | - | - | - | - | - | - | - | - | - | - | ↓ | ↓ | - | - |
| Wu (2017)^127^ | 28 | MR | - | - | - | - | - | - | - | - | - | NA | ↓ | - | - | - | - | - | - | - | - | - | - | - | NA | NA | - | - |
| Ho (2011)^202^ | 8.6 | CFP | - | - | - | - | - | - | - | - | - | ↓ | ↓ | - | - | - | - | - | - | - | - | - | - | - | - | - | - | - |
| Tomany (2004)^2^ | 5.5 | CFP | - | - | - | - | - | - | - | - | - | - | - | - | - | ↑ | - | - | NA | - | - | - | - | - | - | - | - | - |
| Jonasson (2014)^94^ | 5 | CFP | - | - | - | - | - | - | - | - | - | - | - | - | - | NA | - | - | ↑ | - | - | - | - | - | - | - | - | - |
| Klein (2003)^116^ | 10.1 | CFP | - | - | - | - | - | - | - | - | - | - | - | - | - | NA | - | - | ↑ | - | - | - | - | - | - | - | - | - |
| Van Leeuwen (2004)^225^ | 5.2 | CFP | - | - | - | - | - | - | - | - | - | - | - | - | - | NA | - | - | ↑ | - | - | - | - | - | - | - | - | - |
| Shim (2016)^21^ | 4.4 | CFP | - | - | - | - | - | - | - | - | - | - | - | - | - | - | - | - | NA | - | - | - | - | - | - | - | - | - |
| Seddon (2005)^219^ | 4.6 | CFP | - | - | - | - | - | - | - | - | - | - | - | - | - | - | - | - | - | - | NA | - | - | - | - | - | - | - |
| Reynolds (2013)^89^ | 10 | CFP | - | - | - | - | - | - | - | - | - | - | - | - | - | - | - | - | - | - | - | NA | NA | - | NA | ↓ | NA | NA |
| Robman (2007)^210^ | 7 | CFP | - | - | - | - | - | - | - | - | - | - | - | - | - | - | - | - | - | - | - | NA | NA | - | - | - | - | - |
| Seddon (2003)^125^ | 4.6 | CFP | - | - | - | - | - | - | - | - | - | - | - | - | - | - | - | - | - | - | - | NA | NA | - | - | - | - | - |
| Merle (2014)^222^ | 3 | CFP/FAG | - | - | - | - | - | - | - | - | - | - | - | - | - | - | - | - | - | - | - | - | - | - | ↓ | NA | - | - |
| AREDS2 Research Group (2013)^205^ | 4.9 | CFP | - | - | - | - | - | - | - | - | - | - | - | - | - | - | - | - | - | - | - | - | - | - | - | NA | - | - |
| SanGiovanni (2008)^228^ | 6.3 | CFP | - | - | - | - | - | - | - | - | - | - | - | - | - | - | - | - | - | - | - | - | - | - | ↓ | ↓ | - | - |
| Souied (2013)^223^ | 3 | CFP | - | - | - | - | - | - | - | - | - | - | - | - | - | - | - | - | - | - | - | - | - | - | ↓ | ↓ | - | - |
| Merle (2013)^221^ | 2.6 | CFP | - | - | - | - | - | - | - | - | - | - | - | - | - | - | - | - | - | - | - | - | - | ↓ | - | ↓ | - | - |
| **Total ↑** |  |  | **-** | **-** | **-** | **4** | **-** | **-** | **-** | **-** | **2** | **-** | **-** | **1** | **-** | **2** | **-** | **-** | **4** | **1** | **-** | **-** | **-** | **1** | **-** | **-** | **1** | **-** |
| **Total ↓** |  |  | **-** | **1** | **-** | **-** | **-** | **1** | **-** | **-** | **-** | **1** | **3** | **-** | **-** | **-** | **1** | **-** | **1** | **-** | **-** | **-** | **-** | **1** | **4** | **6** | **-** | **-** |
| **Total NA** |  |  | **8** | **6** | **5** | **3** | **1** | **-** | **5** | **5** | **3** | **4** | **2** | **3** | **4** | **8** | **4** | **4** | **6** | **-** | **2** | **7** | **7** | **3** | **4** | **4** | **4** | **5** |

Abbreviations: ↑: increased risk factor; ↓: decreased risk factor; AA: arachidonic acid; ALA: α -linolenic acid; AMD: age-related macular degeneration; ApoA1: apolipoprotein A1, ApoB: Apolipoprotein B, CFP: color fundus photography; DHA: docosahexaenoic acid; EPA: eicosapentaenoic acid; FAG: fluorescein angiography; HDL-C: high-density lipoproteins cholesterol; LA: linoleic acid; LDL-C: low-density lipoproteins cholesterol; MR: medical records; MUFA: mono unsaturated fatty acids; NA: no association found; OCT: optical coherence tomography; SFA: saturated fatty
